# Incidence of nonvalvular atrial fibrillation and oral anticoagulant prescribing in England, 2009 to 2019: A cohort study

**DOI:** 10.1371/journal.pmed.1004003

**Published:** 2022-06-07

**Authors:** Alyaa M. Ajabnoor, Salwa S. Zghebi, Rosa Parisi, Darren M. Ashcroft, Martin K. Rutter, Tim Doran, Matthew J. Carr, Mamas A. Mamas, Evangelos Kontopantelis

**Affiliations:** 1 Department of Pharmacy Practice, Faculty of Pharmacy, King Abdulaziz University, Jeddah, Saudi Arabia; 2 Division of Informatics, Imaging and Data Sciences, School of Health Sciences, Faculty of Biology, Medicine and Health, Manchester Academic Health Science Centre (MAHSC), University of Manchester, Manchester, United Kingdom; 3 Division of Population Health, Health Services Research and Primary Care, School of Health Sciences, Faculty of Biology, Medicine and Health, Manchester Academic Health Science Centre (MAHSC), University of Manchester, Manchester, United Kingdom; 4 Centre for Pharmacoepidemiology and Drug Safety, Division of Pharmacy and Optometry, School of Health Sciences, Faculty of Biology, Medicine and Health, University of Manchester, Manchester, United Kingdom; 5 NIHR Greater Manchester Patient Safety Translational Research Centre (PSTRC), University of Manchester, Manchester, United Kingdom; 6 Division of Diabetes, Endocrinology and Gastroenterology, School of Medical Sciences, Faculty of Biology, Medicine and Health, Manchester Academic Health Science Centre (MAHSC), The University of Manchester, Manchester, United Kingdom; 7 Diabetes, Endocrinology and Metabolism Centre, Manchester University NHS Foundation Trust, Manchester Academic Health Science Centre, Manchester, United Kingdom; 8 Department of Health Sciences, Seebohm Rowntree Building, University of York, York, United Kingdom; 9 Keele Cardiovascular Research Group, Centre for Prognosis Research, Institute for Primary Care and Health Sciences, Keele University, Keele, United Kingdom; Columbia University, UNITED STATES

## Abstract

**Background:**

Atrial fibrillation (AF) is an important risk factor for ischaemic stroke, and AF incidence is expected to increase. Guidelines recommend using oral anticoagulants (OACs) to prevent the development of stroke. However, studies have reported the frequent underuse of OACs in AF patients. The objective of this study is to describe nonvalvular atrial fibrillation (NVAF) incidence in England and assess the clinical and socioeconomic factors associated with the underprescribing of OACs.

**Methods and findings:**

We conducted a population-based retrospective cohort study using the UK Clinical Practice Research Datalink (CPRD) database to identify patients with NVAF aged ≥18 years and registered in English general practices between 2009 and 2019. Annual incidence rate of NVAF by age, deprivation quintile, and region was estimated. OAC prescribing status was explored for patients at risk for stroke and classified into the following: OAC, aspirin only, or no treatment. We used a multivariable multinomial logistic regression model to estimate relative risk ratios (RRRs) and 95% confidence intervals (CIs) of the factors associated with OAC or aspirin-only prescribing compared to no treatment in patients with NVAF who are recommended to take OAC. The multivariable regression was adjusted for age, sex, comorbidities, socioeconomic status, baseline treatment, frailty, bleeding risk factors, and takes into account clustering by general practice. Between 2009 and 2019, 12,517,191 patients met the criteria for being at risk of developing NVAF. After a median follow-up of 4.6 years, 192,265 patients had an incident NVAF contributing a total of 647,876 person-years (PYR) of follow-up. The overall age-adjusted incidence of NVAF per 10,000 PYR increased from 20.8 (95% CI: 20.4; 21.1) in 2009 to 25.5 (25.1; 25.9) in 2019. Higher incidence rates were observed for older ages and males. Among NVAF patients eligible for anticoagulation, OAC prescribing rose from 59.8% (95% CI: 59.0; 60.6) in 2009 to 83.2% (95% CI: 83.0; 83.4) in 2019. Several conditions were associated with lower risk of OAC prescribing: dementia [RRR 0.52 (0.47; 0.59)], liver disease 0.58 (0.50; 0.67), malignancy 0.74 (0.72; 0.77), and history of falls 0.82 (0.78; 0.85). Compared to white ethnicity, patients from black and other ethnic minorities were less likely to receive OAC; 0.78 (0.65; 0.94) and 0.76 (0.64; 0.91), respectively. Patients living in the most deprived areas were less likely to receive OAC 0.85 (0.79; 0.91) than patients living in the least deprived areas. Practices located in the East of England were associated with higher risk of prescribing aspirin only over no treatment than practices in London (RRR 1.22; 95% CI 1.02 to 1.45). The main limitation of this study is that these findings depends on accurate recording of conditions by health professionals and the inevitable residual confounding due to lack of data on certain factors that could be associated with under-prescribing of OACs.

**Conclusions:**

The incidence of NVAF increased between 2009 and 2015, before plateauing. Underprescribing of OACs in NVAF is associated with a range of comorbidities, ethnicity, and socioeconomic factors, demonstrating the need for initiatives to reduce inequalities in the care for AF patients.

## Abstract 2

Alyaa Ajabnoor and co-workers study incidence of non-valvular atrial fibrillation in England and apparent inequalities in care.

## Author summary

**Why was this study done?:**

- Previous research has shown that the incidence of atrial fibrillation (AF) is expected to increase, although limited data exists in the UK.
- AF is one of the most important risk factors for future stroke risk, and the treatment of choice is anticoagulation.
- Many patients with nonvalvular atrial fibrillation (NVAF) are not receiving anticoagulant therapy for stroke prevention, although it remains uncertain whether there is an association with patient ethnicity and socioeconomic status.

**What did the researchers do and find?:**

- We used electronic health records from GP practices in England to estimate the incidence of NVAF between the 2009 and 2019. We have also explored the clinical and socioeconomic factors associated with prescribing oral anticoagulants (OACs).
- We have found that the incidence of NVAF in England has increased during the past decade and plateaued from 2015 onwards.
- There are racial and socioeconomic inequalities in the prescribing of OACs in England, with low socioeconomic status and black or other non-white ethnicities associated with the prescription of aspirin only or no treatment compared to white patients or those with higher socioeconomic status.

**What do these findings mean?:**

- There are many patients at risk for stroke who are not receiving anticoagulants, and clinical and sociodemographic factors play a role in the underprescribing of OACs
- Our findings suggest that, in order to improve AF outcomes, these inequalities need to be addressed through equitable interventions to improve OAC prescribing to prevent stroke events and reduce mortality.

## Introduction

Atrial fibrillation (AF) is the most common sustained cardiac arrhythmia with an estimated global prevalence of 37.574 million cases (0.51% of the world population) [1]. In Europe alone, the current estimated prevalence of AF is between 1% and 4%, along with an expected increase in the incidence of AF worldwide in the next decades due to population ageing [2,3]. AF is associated with increased risk of stroke, cardiovascular comorbidities, and mortality and currently accounts for 1% of the total healthcare expenditure in the United Kingdom (UK) [4]. Effective stroke prevention can be achieved with oral anticoagulant (OAC) treatment. Vitamin K antagonists (VKAs) have long been the only available OACs for stroke prevention in patients with AF, but the use of VKAs is limited by the narrow therapeutic interval, which requires frequent monitoring and dose adjustments [5]. In order to overcome these limitations, non-vitamin K antagonist oral anticoagulants (NOACs), whose use is supported by randomised controlled trials [6–8], were introduced. Previous observational studies from the UK using primary care data have found that the increase in proportion of AF patients receiving anticoagulants has taken place over more than a decade [9,10]. While it is not possible for the changes in OAC prescribing to be underpinned by a single factor, the previously reported increase in OACs uptake may have in part be explained by the licence of the first NOAC agent for stroke prophylaxis in AF in the UK in 2011 [11–13]. Whereas the increase in the rate of OACs prescribing after 2011 corresponds also to a change in European Society of Cardiology guidelines to recommend the treatment of moderate-risk to high-risk AF patients with OAC rather than antiplatelet, as well as a change in the Quality and Outcomes Framework (QOF) to incentivise prescribing of anticoagulants [14–16].

Based on earlier studies, rates of OAC prescribing varies by time and setting [17]. While studies from European countries have reported a very high rates of OAC prescribing (>80%) [18,19], observational studies from the UK showed that the proportion of patients with AF who remained without anticoagulation was around 21% and >40% in older people [20,21]. Since long-term anticoagulant therapy improves clinical outcomes in AF patients, it is necessary to understand and investigate the reasons behind the underuse of OACs in patients at risk for stroke. There may be several reasons for OACs underuse including frailty or prior bleeding [22]; however, more detailed and contemporary understanding of the reasons behind the underprescribing of OACs may enhance guideline-directed anticoagulant prophylaxis in patients with AF. With this in mind, we aim to describe temporal trends of nonvalvular atrial fibrillation (NVAF) incidence in England including OACs and antiplatelet prescribing patterns. We also aim to assess the clinical and socioeconomic factors associated with underprescribing of OACs after NVAF diagnosis in a national primary care population representative of contemporary clinical practice.

## Methods

### Data source

We conducted a population-based retrospective cohort study using the Clinical Practice Research Datalink (CPRD) GOLD and Aurum databases. The CPRD is a longitudinal primary care database of anonymised general practitioner (GP) medical records in the UK and contains consultation records, patient demographic information, diagnoses, drug prescriptions, and referrals to secondary care. CPRD data have been used extensively in pharmacoepidemiology research [23–26]. The CPRD GOLD database collects data from practices using the VISION computer system, and CPRD Aurum includes data from the EMIS computer system. The July 2020 database release for CPRD GOLD from which the study cohort was sampled included data from 374 contributing general practices in England, and the July 2020 release for CPRD Aurum included data on 1,383 general practices. Practices that migrated from GOLD to Aurum were only included in the latter. We also obtained small area-level linkage on practice and patients’ residence postcodes that included measure of area-level deprivation specifically the Index of Multiple Deprivation (2015 IMD for England) [27]. The IMD is a composite score measured as the weighted sum of the individual indices of 7 domains of deprivation including the following: education, finance, health, access to services, and crime [27].

### Participants

**Fig 1:**
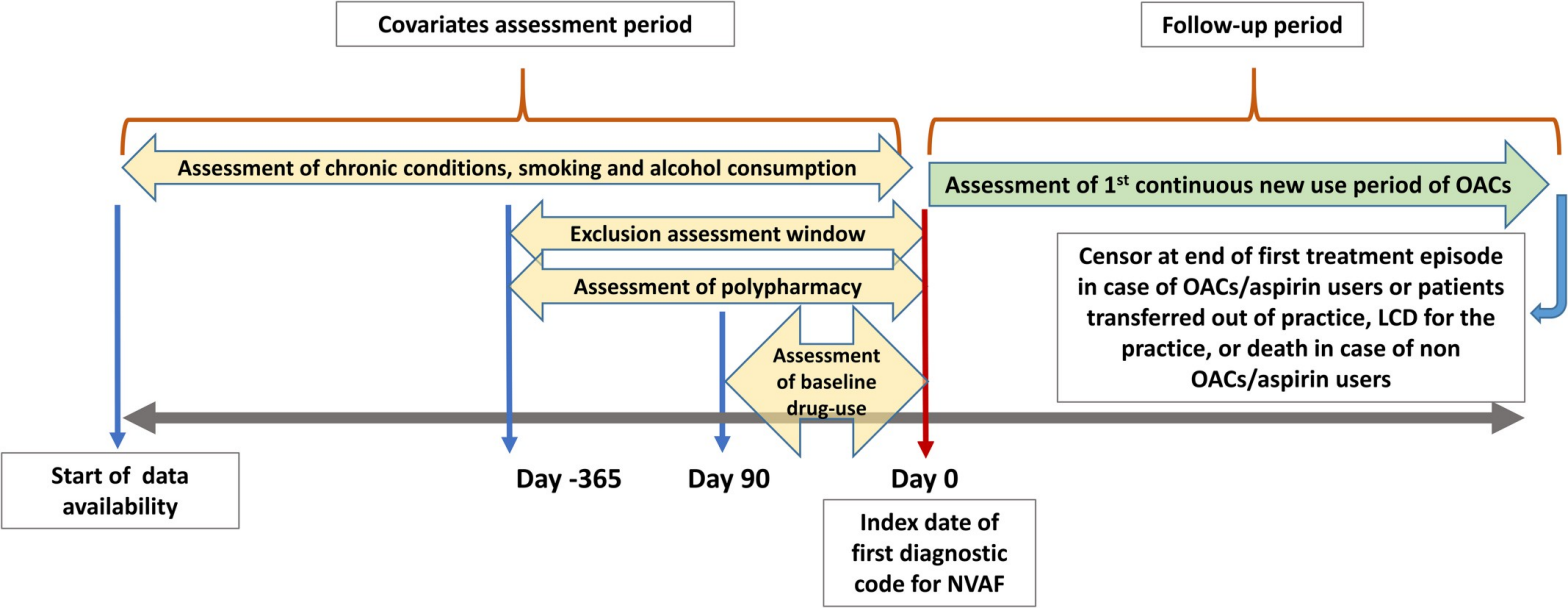
Graphical depiction of patient’s follow-up and assessment of OACs exposure status. LCD, late collection date; NVAF, nonvalvular atrial fibrillation; OAC, oral anticoagulant.

The database was screened to identify a first-ever clinical record of AF such as paroxysmal AF or AF and atrial flutter, occurring from 1 January 2009 until 31 December 2019. Records for NVAF were identified using Read codes in CPRD GOLD or using both SNOMED/EMIS and Read codes in CPRD Aurum. Diagnostic codes were independently reviewed by an expert clinician (MAM), and medication lists were reviewed by the first author (AMA). The codes used to produce the data for this study can be found in https://clinicalcodes.rss.mhs.man.ac.uk/ [28]. Eligible patients were adults aged ≥18 years and registered in a general practice in England for at least 1 year before NVAF diagnosis. Patients with heart valve problems before NVAF diagnosis were excluded. Additional exclusion criteria were applied within a lookback period of 12 months before NVAF diagnosis: records of irregular heartbeats or cardioversion, records of atrial flutter alone with no mention of AF, previous use of oral or parenteral anticoagulants >14 days prior to NVAF diagnosis, and previous use of quinidine, sotalol, amiodarone, flecainide, or propafenone. Both GOLD and Aurum cohorts were combined and analysed together. Patients’ follow-up started from the index date of NVAF diagnosis and continued until the earliest of the following: end of study observation period (31 December 2019), patients transferred out of practice, last collection date for the practice, death, or the end of first treatment episode in case of OACs or aspirin users (Fig 1).

### Incidence rates

When calculating incidence rates, the variation in the number of practices contributing data to the CPRD GOLD database was considered, since the number of practices in England contributing to GOLD has greatly reduced in the last few years, reflecting the drop in the market share of the VISION computer system [29]. Therefore, denominator estimates were restricted to patients registered at the start of the year with a general practice that contributed data throughout the study period (from 2009 to 2019). The numerators were estimated as the number of patients included in the denominator with a first recorded diagnosis of NVAF during the same year. Sex-specific estimates of incidence rates are presented throughout. We report stratified analyses by age-group, practice-IMD from 1 (least) to 5 (most deprived), and region.

### Exposure to anticoagulants

OACs available in the UK were identified from the British National Formulary (BNF). VKAs included warfarin, phenindione, and acenocoumarol, whereas NOACs included dabigatran, rivaroxaban, apixaban, and edoxaban. Drug exposure status was categorised to either OACs (VKAs or NOACs), aspirin only, or no treatment. To ascertain exposure to OACs, the study focused on the first continuous treatment episode of OAC, defined as sequential prescriptions of the same drug within a grace period of 30 days after the expected end of the previous prescription. A gap of 30 days or less between the end of days of supply of 1 prescription and the next was ignored and assumed to be a continuous treatment episode; this was to account for the real-world setting and potential delays in gaining a repeat prescription. In case of NOACs, the quantity issued was estimated by dividing the number of tablets prescribed by the approved number of daily doses (twice daily for dabigatran and apixaban and once a day for rivaroxaban and edoxaban). However, if the quantity of NOACs issued was not available, the quantity was estimated using the mean number of NOAC tablets prescribed for the same drug for that patient, or the overall mean for that drug if patient-specific data was not available. Since precise dosages for VKAs were not available because they vary according to INR measurements and are not consistently recorded in general practice, the median time between all previous sequential prescriptions of VKAs for each patient were used to estimate days of supply, and INR measurements if reported were treated as an indicator for VKA exposure and therefore treated in the same way as prescriptions. Additionally, if a patient was prescribed aspirin with no OAC, an exposure status of aspirin only was assigned to those patients.

### Study covariates

We extracted baseline information on the following demographic and clinical risk factors: age, sex, ethnicity, IMD quintile, calendar index year, and body mass index (BMI) (within 2 years before NVAF diagnosis). Any previous records before NVAF diagnosis on comorbidities, smoking, and alcohol consumption were identified. Comorbidities were also defined according to the Charlson comorbidity index (CCI) [30] and the electronic frailty index (eFI) [31]. Polypharmacy was assessed at baseline using the common definition of the concomitant use of 5 or more medications during 1 year before NVAF diagnosis [32]. Baseline drug use of other medications was assessed before NVAF diagnosis, if a patient received at least 1 prescription of a certain drug within 90 days before diagnosis. Medications were explored if they affect bleeding or stroke risk, or known to enhance or suppress the efficacy of OACs: antiplatelets (P2Y12 inhibitors); non-dihydropyridine calcium channel blockers; proton pump inhibitors; antibiotics (macrolides, fluoroquinolones, and sulfonamides); antidepressants (selective serotonin reuptake inhibitors (SSRIs) and selective norepinephrine reuptake inhibitors (SNRIs)); anticonvulsants (phenytoin, phenobarbital, and carbamazepine); nonsteroidal anti-inflammatory drugs (NSAIDs); corticosteroids; statins; systemic azoles; and ciclosporin. Although it is difficult to predict whether prescriptions for a treatment at baseline are still continuing at any given time, we have assumed that if a prescription was issued in the 90 days prior to diagnosis, then the treatment is still continuing at that time, considering that it is likely to be the case for most medications of interest that are for chronic conditions. However, we acknowledge that this could overestimate exposure to these drugs.

Stroke risk was assessed using the CHA_2_DS_2_-VASc score, which consists of 8 categories, with points given for each of the following: congestive heart failure, hypertension, age ≥75 years (× 2 points), diabetes mellitus, prior stroke or transient ischemic attack (TIA) or thromboembolism (× 2 points), vascular disease, age 65 to 74 years, and sex category [33]. According to the ESC guidelines [34], anticoagulation should be considered with a CHA_2_DS_2_-VASc score of 1 in males, or 2 in females, and anticoagulation is recommended with a CHA_2_DS_2_-VASc score of ≥2 in males, or ≥3 in females. Therefore, we classified the eligibility or OAC into the following: “not eligible for OAC” if CHA_2_DS_2_-VASc score is equal to 0 in males, or 1 in females; and “eligible for OAC” if CHA_2_DS_2_-VASc score ≥1 in males, or ≥2 in females. Baseline bleeding risk was assessed using the HAS-BLED score [35], which consists of 9 points, one for each of the following: hypertension, abnormal kidney or liver function (1 point each), stroke, history of bleeding or predisposition, labile INR, elderly (>65 years), and drugs/alcohol concomitantly (1 point each). Since INR measurements are not consistently reported in CPRD, a modified HAS-BLED score was used, which does not include the INR element.

### Statistical analysis

Baseline characteristics are presented as frequencies (%) for categorical data, medians, and interquartile ranges (IQRs) for nonnormally distributed continuous data, or means and SD for normally distributed continuous data. Data are stratified by sex, IMD quintile, and year of diagnosis. Number and percentage of records with missing data are displayed for variables with missing entries.

Overall and annual standardised incidence rates were calculated by age at diagnosis and by IMD quintile. These were obtained through direct standardisation, by applying category specific rates from each subgroup to the demographic distribution of the whole CPRD population to produce group-specific rates that would have been observed if the subgroups all had the same distribution. A similar approach using data from the CPRD has been reported previously [36]. Calculated incidence rates were expressed per 10,000 person-years (PYR) at risk, with 95% confidence intervals (CIs).

Proportions of patients prescribed OACs, aspirin only, or no treatment were calculated annually for patient’s eligible for OAC (CHA_2_DS_2_-VASc score ≥1 in males, or ≥2 in females). To identify predictors of OAC prescribing, aspirin prescribing only, or no treatment, a multivariable multinomial logistic regression model was fitted with practice characteristics (practice level IMD, list size, and region), patients sociodemographic characteristics, baseline comorbidities, and drug use as independent variables. In this model, missing baseline BMI was imputed by an interpolation algorithm that has been used in previous studies using the CPRD [37]. The model only included patients who were eligible for OAC and were recommended to take anticoagulation as per their baseline CHA_2_DS_2_-VASc score. The model takes account of clustering by general practice and was adjusted for age, sex, comorbidities, socioeconomic status, baseline treatment, frailty, and bleeding risk factors. CHA_2_DS_2_-VASc, HAS-BLED, CCI, and eFI scores were not included in these models, and only the score components were individually included. In 2 additional models, interactions between key variables were included. The first interaction model included the interactions of patient-level IMD with ethnicity, and patient-level IMD with practice region. The second interaction model included the interaction terms of practice-level IMD with ethnicity, and practice-level IMD with practice region. Both models were adjusted for age, sex, comorbidities, and bleeding risk (HAS-BLED). Following these models, the post-estimation command *margins* was used to compute the adjusted probabilities of prescribing OAC, aspirin only, or no treatment, across the strata of interest defined by these interactions. All statistical analyses were performed using Stata v16.

In sensitivity analyses, we estimated the incidence rates of NVAF in England from CPRD GOLD and Aurum databases separately, including patients registered with a general practice that contributed data at any time point during the study period (for 11 years or less). This was done for 2 reasons. First, to investigate any differences in incidence rates in CPRD GOLD and Aurum, which may be attributed to regional variability [29]. Second, to explore the role of practice variability over time on incidence rates, especially for CPRD GOLD with a large drop in practice numbers in later years due to migration to a different computer system and the Aurum database. In response to comments from peer reviewers, we performed another sensitivity analysis to identify predictors of OAC prescribing versus no treatment using multivariable binomial logistic regression. In this analysis, the “no treatment” group included patients who received aspirin only. This was done to compare the performance of the multinomial logistic regression model to that of the binomial logistic regression where aspirin was not considered as a treatment on its own.

## Results

### Patients and practice characteristics

**Fig 2:**
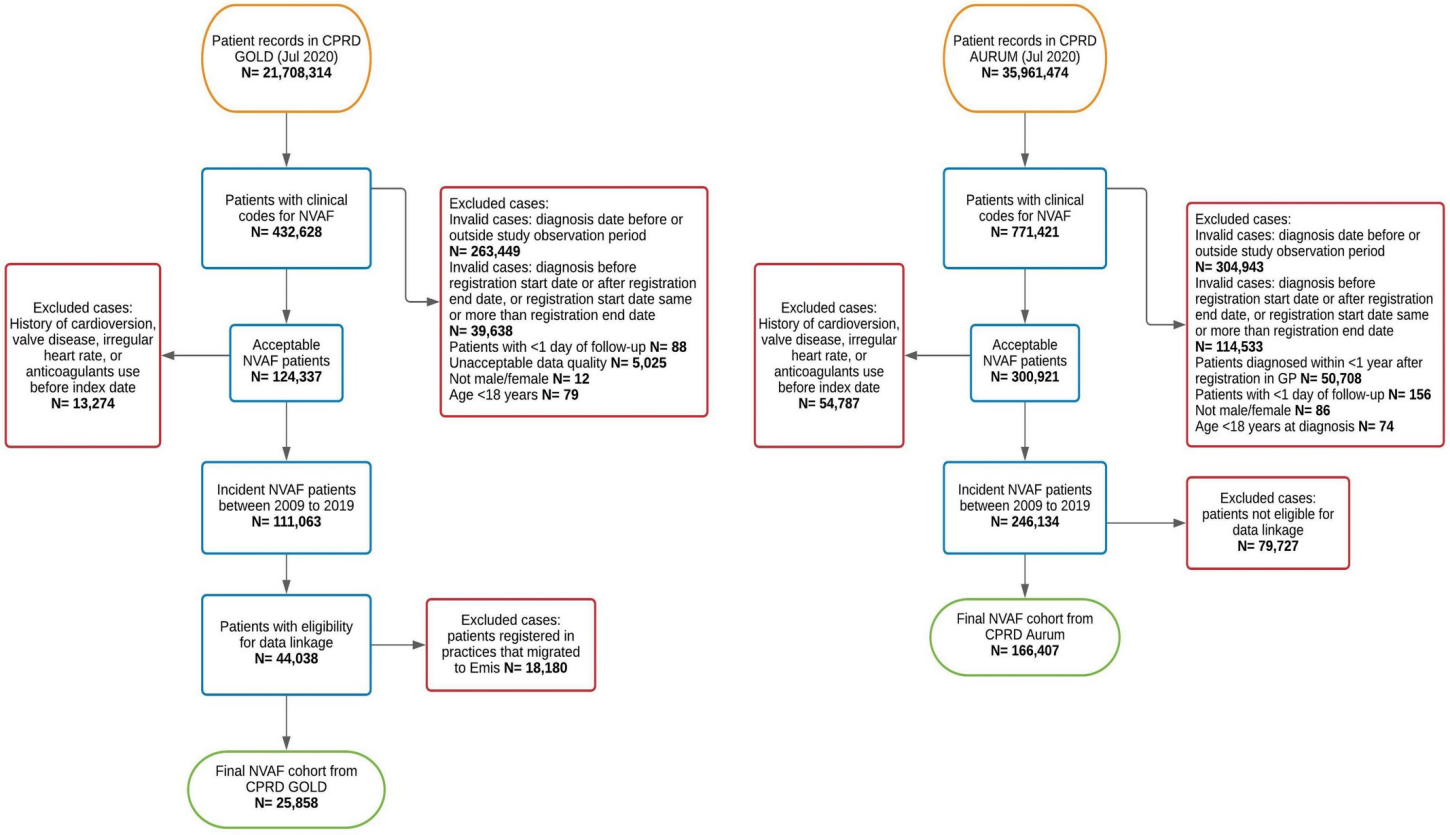
A flow chart of the study inclusion/exclusion criteria in CPRD GOLD and Aurum. CPRD, Clinical Practice Research Datalink; GP, general practice; NVAF, nonvalvular atrial fibrillation.

After applying all selection criteria, 25,858 patients from CPRD GOLD and 166,407 patients from CPRD Aurum were included in the study cohort (Fig 2), contributing a total of 73,950 and 573,926 PYR of follow-up, respectively. This study included patients from a total of 1,126 GP practices across England. Practices in the most deprived areas (IMD 4 and 5) represented 22.3% (*n =* 251) and 24.3% (*n* = 274), respectively, of all included practices (S1 Table). Table 1 provides the baseline characteristics of the study population for both cohorts. Patients’ mean age was 75.1 (12.3), 53.3% (*n* = 102,503) were males, and more than 30% were either overweight 22.5% (*n* = 43,202) or obese 23.2% (*n* = 44,691). The majority of patients were from white ethnicity 92.4% (*n =* 177,663), and 91.1% (*n* = 175,172) were eligible for OAC according to their CHA_2_DS_2_VASc score. Many patients had some degree of frailty, specifically mild frailty 39% (*n* = 74,949), moderate frailty 23.1% (*n* = 44,480), and severe frailty 9.8% (*n* = 18,842) according to their eFI. At baseline, 51.3% (*n* = 98,560) were at high risk of bleeding according to HAS-BLED score of ≥3, and 55.1% (*n* = 105,924) had a CCI of ≥2.

**Table 1:** Baseline characteristics of patients newly diagnosed with NVAF in England in the CPRD, from 2009 to 2019.

	CPRD GOLD cohort	CPRD Aurum cohort	Overall
	*n* = 25,858	n = 166,407	n = 192,265
Mean age (SD)	74.7 (12.2)	75.1 (12.3)	75.05 (12.3)
**Sex, n(%)**			
Male	13,958 (54)	88,545 (53.2)	102,503 (53.3)
Female	11,900 (46)	77,862 (46.8)	89,762 (46.7)
**Ethnicity, n(%)**			
White	24,315 (94)	153,348 (92.1)	177,663 (92.4)
Black	128 (0.6)	1,555	1,683 (0.9)
Asian	249 (1)	2,360 (1.4)	2,609 (1.4)
Other	226 (0.9)	1,416 (0.9)	1,642 (0.8)
Unknown	940 (3.5)	7,728 (4.6)	8,668 (4.5)
**BMI, kg/m** ^**2**^ **, n(%)**			
<18.5	620 (2.4)	2941 (1.8)	3,561 (1.9)
≥18.5 to <25	4,332 (16.8)	28,402 (17.1)	32,734 (17)
≥25 to <30	5695 (22)	37,507 (22.5)	43,202 (22.5)
≥30	5,695 (22.6)	38,834 (23.3)	44,691 (23.2)
Unknown	9,354 (36.2)	58,723 (35.3)	68,077 (35.4)
Patient-level IMD, n(%)			
1 –least deprived	5,916 (22.9)	41,386 (24.9)	47,302 (24.6)
2	5,428 (21)	37,643 (22.6)	43,071 (22.4)
3	6,050 (23.4)	33,710 (20.3)	39,760 (20.7)
4	4,830 (18.7)	29,011 (17.4)	33,841 (17.6)
5 –most deprived	3,634 (14)	24,657 (14.8)	28,291 (14.7)
CHA_2_DS_2_-VASc score, n(%)			
0	1,575 (6.1)	10,231 (6.1)	11,806 (6.1)
1	2,733 (10.6)	16,903 (10.2)	19,636 (10.2)
≥2	21,550 (83.3)	139,273 (83.7)	160,823 (83.7)
Eligibility for OAC, n(%)			
Not eligible for OAC	2,292 (8.8)	14,801 (8.9)	17,093 (8.9)
Eligible–consider OAC	3137 (12.1)	19,598 (11.8)	22,735 (11.8)
Eligible–OAC recommended	20,429 (79)	132,008 (79.3)	152,437 (79.3)
**HAS-BLED Score, n(%)**			
0–1	5,752 (22.2)	37,192 (22.3)	42,944 (22.3)
2	6,722 (26)	44,039 (26.5)	50,761 (26.4)
≥3	13,384 (51.8)	85,176 (51.2)	98,560 (51.3)
CCI, n(%)			
0	8,348 (32.3)	49,159 (29.5)	57,507 (29.9)
1	4,580 (17.7)	24,254 (14.6)	28,834 (15)
≥2	12,930 (50)	92,994 (55.9)	105,924 (55.1)
Frailty Index (eFI), n(%)			
Mostly well	6,588 (25.5)	46,211 (27.8)	52,799 (27.5)
Mild frailty	10,021 (38.7)	65,379 (39.3)	74,400 (39.2)
Moderate frailty	6,425 (24.9)	39,044 (23.4)	45,469 (23.6)
Severe frailty	2,824 (10.9)	15,773 (9.5)	18,597 (9.7)

BMI, body mass index; CCI, Charlson comorbidity index; CPRD, Clinical Practice Research Datalink; eFI, electronic frailty index; IMD, index of multiple deprivation; OAC, oral anticoagulant;.

### Incidence of NVAF

During the period between 2009 to 2019, 12,517,191 patients met the criteria for being at risk of developing NVAF. After a median follow-up of 4.6 years, the overall incidence rate of NVAF per 10,000 PYR (95% CI) in CPRD was 20.8 (20.4; 21.1) in 2009 and steadily increased over the years until reaching a stable rate in 2015 at 25.9 (25.5; 26.3) (S2 Table). By sex, the annual standardised rates were higher in males than in females starting at 24.2 (23.6; 24.8) for males and 17.5 (17.0; 18.0) for females in 2009 and continued to increase for both sexes until plateauing in 2015 at 30.9 (30.2; 31.6) for males and 21.4 (20.8; 21.9) for females. The effect of practice dropout from the CPRD GOLD database was noticeable in the first sensitivity analysis that looked at the incidence rate of NVAF, by including patients from GP practices that contributed for at least 1 year. Incidence rates in Aurum were steadily increasing over the years similar to the main analysis, but in CPRD GOLD, a peak in the incident cases for males was observed in 2012 and for females in 2016 followed by a steady decline afterwards (S3 Table and S1 Fig). However, in the second sensitivity analysis that was exclusive for GP practices that contributed throughout the study period (for 11 years), incidence rates of NVAF in CPRD GOLD were more similar to CPRD Aurum (S4 Table and S2 Fig).

**Fig 3:**
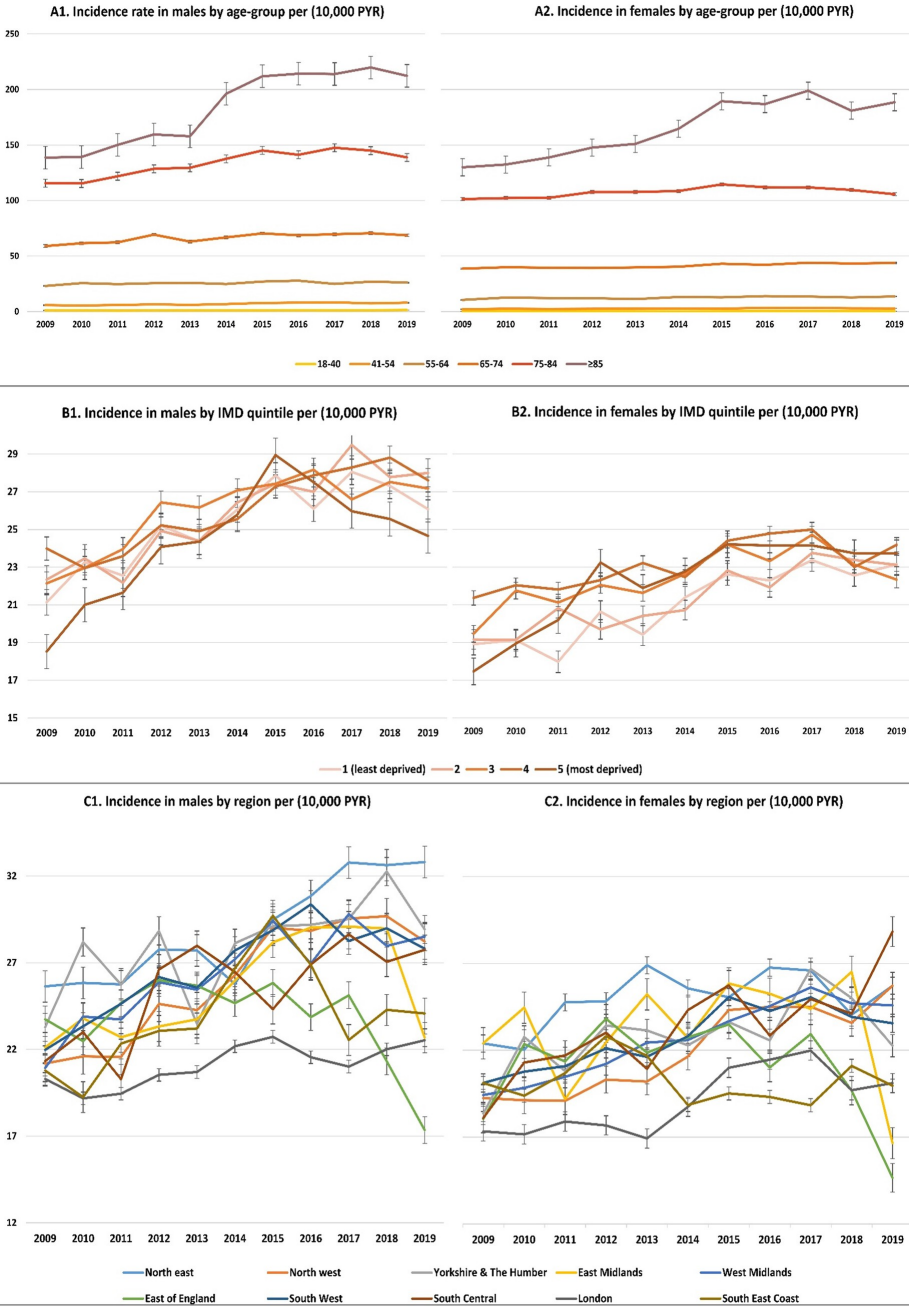
Sex-specific annual standardised incidence rates per 10,000 PYR (95% CI) by (A) age-group; (B) neighbourhood deprivation quintile; and (C) region. CI, confidence interval; IMD, index of multiple deprivation; PYR, person year at risk.

Incidence rates by age-group showed an overall increase in rates over the years in all age categories, with the highest rates observed in older age groups, across both sexes (Fig 3, A1 and A2). Incidence rates by deprivation quintiles showed an overall increase in incidence rates over time across strata (Fig 3, B1 and B2), but patterns were different for males and females. Overall, incidence rates were the highest in the North East, and lowest in London for both sexes (Fig 3, C1 and C2).

### Treatment patterns

**Fig 4:**
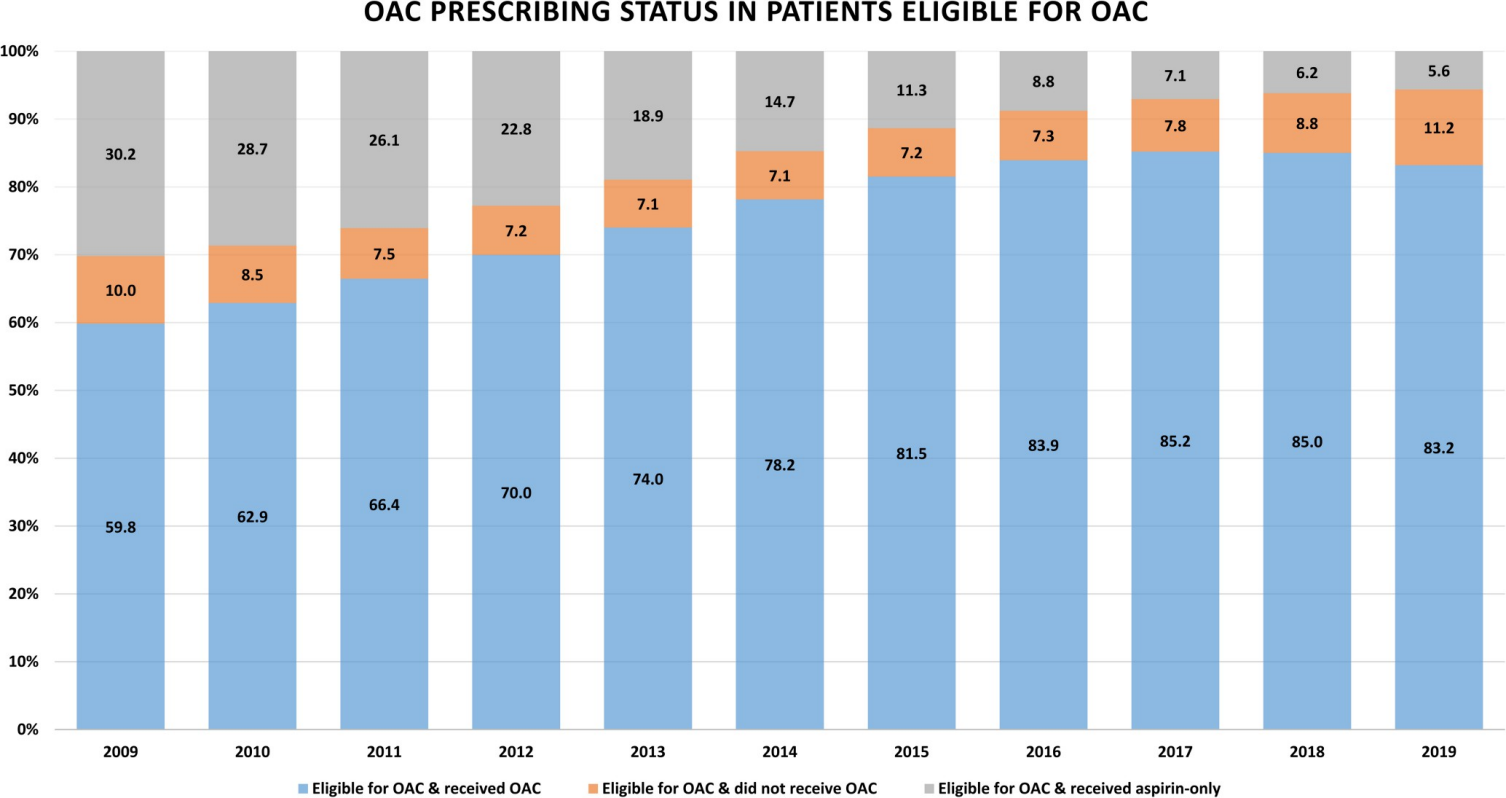
Proportions of patients prescribed OACs (VKA or NOAC), aspirin only, or no treatment for patients eligible for OAC. NOAC, non- vitamin K antagonist oral anticoagulant; OAC, oral anticoagulant; VKA, vitamin K antagonist.

**Fig 5:**
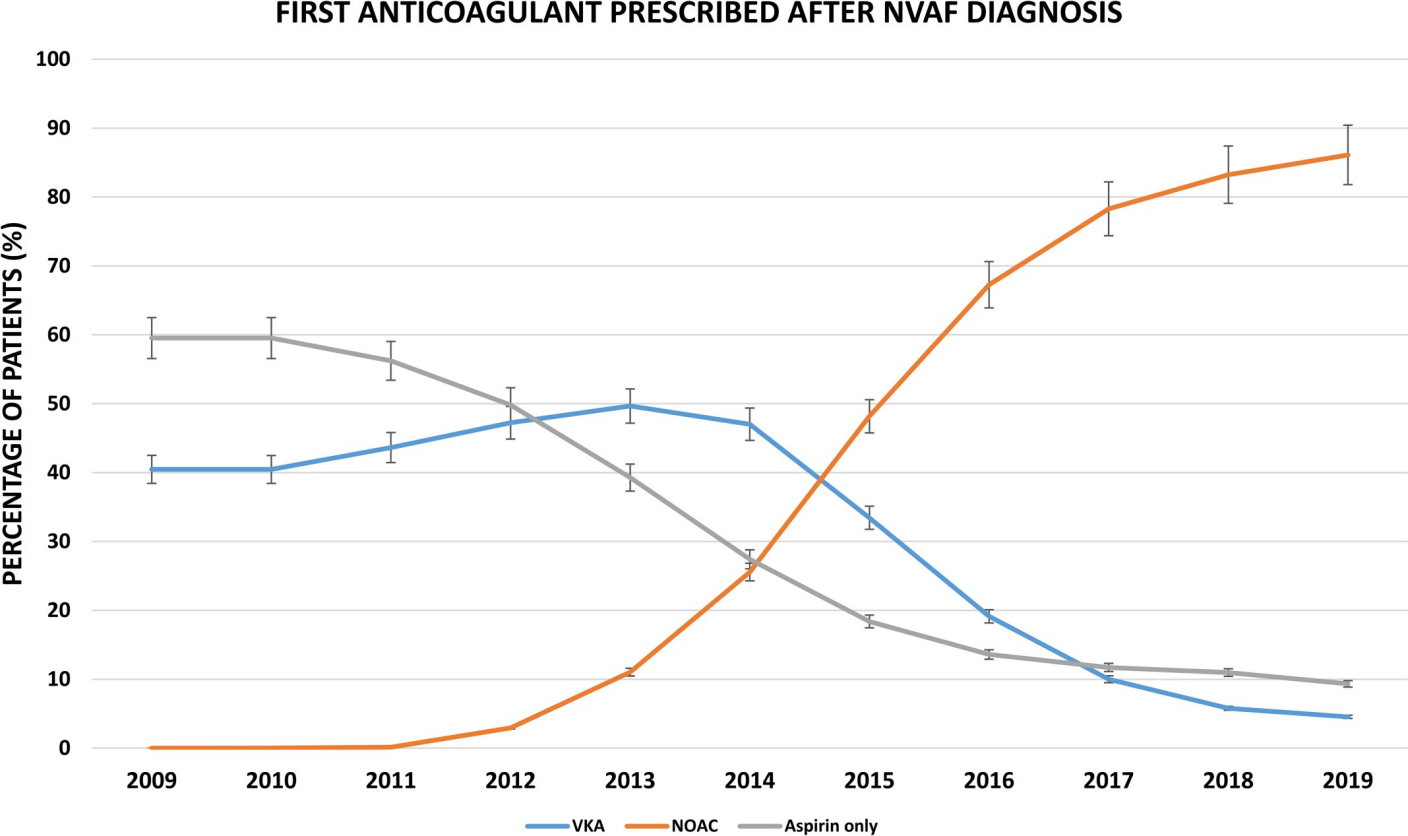
Proportion of patients prescribed first treatment after NVAF diagnosis and 95% CI stratified by the type of drug initiated, from 2009 to 2019. CI, confidence interval; NOAC, non- vitamin K antagonist oral anticoagulant; NVAF, nonvalvular atrial fibrillation; VKA, vitamin K antagonist.

Among patients who are eligible for anticoagulation (both considered and recommended to take OAC), OAC prescribing increased from 59.8% (95% CI: 59.0; 60.6) in 2009 to 83.2% (95% CI: 83.0; 83.4) in 2019 (Fig 4 and S5 Table). In contrast, the prescribing of aspirin only decreased from 30.2% (95% CI: 29.5; 31.0) in 2009 to 5.6% (95% CI: 5.5; 5.8) in 2019. The proportion of patients who did not receive any treatment increased slightly from 7.5% (95% CI: 7.2; 7.7) in 2011 to 11.2% (95% CI: 11.0; 11.4) in 2019. Moreover, the proportion of patients with NVAF who started on aspirin only declined from 59.5% (*n =* 6,632) in 2009 to 9.3% (*n* = 1,112) in 2019 (Fig 5 and S6 Table). Use of VKAs increased from 40.5% (*n* = 4,509) in 2009 to 49.7% (*n =* 6,670) in 2013, then started to decline in 2014 reaching 4.6% (*n* = 542) in 2019. In contrast, the use of NOACs increased over time, from <1% in 2010 to 86.1% (*n* = 10,256) in 2019. As for NVAF patients not eligible for OAC, the proportion of patients not receiving anticoagulation have increased from 22.7% (95% CI: 21; 25) in 2009 to 36.9% (95% CI: 36; 38) in 2019 (S7 Table).

Table 2 shows patient characteristics by type of treatment. The overall proportions of patients receiving treatment was 30.9% (*n =* 59,429) for VKA, 37.2% (*n* = 71,437) for NOAC, 12.3% (*n* = 23,590) for aspirin only, and 19.7% (*n =* 37,809) received no treatment. Of patients who received aspirin only 40.5% (*n* = 9,564) were aged ≥85 years. In the no-treatment group, approximately 80% of patients were eligible for OAC based on their CHA_2_DS_2_-VASc score. Patients receiving no treatment had similar levels of comorbidity (CCI ≥2) 52.6% (*n =* 19,894) to patients treated with NOACs 55.2% (*n* = 39,434). Of patients in the aspirin-only group, 63% (*n* = 14,847) were at high bleeding risk (HAS-BLED score ≥3), and the proportion of high bleeding risk was lowest in the no-treatment group 40.7% (*n* = 15,373). The most common baseline comorbidities observed among all treatment groups were the following: hypertension 63.9% (*n* = 122,789), ischaemic heart disease 29.5% (*n =* 56,713), chronic kidney disease 23.3% (*n* = 44,787), diabetes 21.5% (*n* = 41,236), anaemia 17% (*n* = 32,729), and history of stroke or TIA 15.3% (*n* = 29,369).

**Table 2:** Patients’ characteristics by first continuous treatment episode of OAC.

	VKA	NOAC	Aspirin only	No treatment
	*n* = 59,429	*n* = 71,437	*n* = 23,590	*n* = 37,809
**Sex, n(%)**				
Male	32,790 (55.2)	38,396 (53.7)	11,587 (49.1)	19,730 (52.2)
Female	26,639 (44.8)	33,041 (46.3)	12,003 (50.9)	18,079 (47.8)
**Age group, n(%)**				
18–40 years	315 (0.5)	293 (0.4)	92 (0.4)	1,659 (4.4)
41–54 years	2,487 (4.2)	2,916 (4.1)	808 (3.4)	3,922 (10.4)
55–64 years	6,679 (11.2)	8,274 (11.5)	2,071 (8.8)	4,762 (12.6)
65–74 years	16,949 (28.6)	20,445 (28.6)	3,907 (16.6)	6,549 (17.3)
75–84 years	23,600 (39.7)	25,400 (35.6)	7,148 (30.3)	9,429 (24.9)
≥85 years	9,399 (15.8)	14,109 (19.8)	9,564 (40.5)	11,488 (30.4)
**Ethnicity, n(%)**				
White	56,046 (94.3)	65,698 (91.9)	22,082 (93.6)	33,837 (89.5)
Black	422 (0.7)	558 (0.8)	228 (0.9)	475 (1.3)
Asian	746 (1.3)	985 (1.4)	350 (1.5)	528 (1.4)
Other	410 (0.7)	593 (0.8)	188 (0.8)	451 (1.2)
Unknown	3,603 (3)	742 (5)	742 (3.4)	2,518 (6.7)
**Patient-level IMD, n(%)**				
1 –least deprived	14,604 (24.6)	18,608 (26.1)	5,044 (21.4)	9,046 (24)
2	13,324 (22.4)	16,332 (22.9)	5,100 (21.6)	8,315 (21.9)
3	12,579 (21.2)	14,399 (20.2)	5,021 (21.3)	7,761 (20.5)
4	10,688 (17.9)	11,988 (16.8)	4,446 (18.9)	6,719 (17.8)
5 –most deprived	8,234 (13.9)	10,110 (14.2)	3,979 (16.8)	5,968 (15.8)
Time to first treatment after diagnosis (days)				-
Mean (SD)	114 (229)	292 (653)	60 (183)	
Median (IQR)	21 (4 to 70)	21 (4 to 108)	17 (5 to 39)	
BMI, kg/m^2^, n(%)				
<18.5	746 (1.3)	960 (1.3)	706 (3)	1,149 (3)
≥18.5 to <25	8,951 (15.1)	11,305 (15.8)	5,110 (21.7)	7,368 (19.5)
≥25 to <30	14,351 (24.1)	16,793 (23.5)	5,007 (21.2)	7,051 (18.7)
≥30	16,139 (27.1)	18,436 (25.8)	4,079 (17.3)	6,037 (16)
Unknown	19,242 (32.4)	23,943 (33.6)	8,688 (36.8)	16,204 (42.8)
**Smoking status, n(%)**				
Current smoker	11,580 (19.5)	14,670 (20.6)	4,980 (21.1)	8741 (23.1)
Ex-smoker	30,280 (50.9)	36,943 (51.7)	11,393 (48.3)	17,231 (45.6)
Never smoker	17,355 (29.2)	19,604 (27.4)	7,113 (30.2)	11,503 (30.4)
Unknown	214 (0.4)	220 (0.3)	104 (0.4)	334 (0.9)
**Alcohol consumption status, n(%)**				
Nondrinker	7,048 (11.9)	8,455 (11.8)	3,472 (14.7)	4,896 (12.9)
Former drinker	838 (1.4)	797 (1.1)	405 (1.7)	481 (1.3)
Light drinker	7,021 (11.8)	7,572 (10.6)	2,623 (11.1)	3,842 (10.2)
Moderate drinker	30,371 (51.1)	36,821 (51.6)	10,402 (44.1)	17,350 (45.9)
Heavy drinker	5,496 (9.2)	8,353 (11.7)	1,924 (8.2)	4,137 (10.9)
Unknown	8,655 (14.6)	9,439 (13.2)	4,764 (20.2)	7,103 (18.8)
CHA_2_DS_2_-VASc Score, n(%)				
0	2,832 (4.8)	3,145 (4.4)	827 (3.5)	5,002 (13.2)
1	5,540 (9.3)	7,122 (10)	1,780 (7.6)	5,194 (13.8)
≥2	51,057 (85.9)	61,170 (85.6)	20,983 (88.9)	27,613 (73)
**Eligibility for OAC, n(%)**				
Not eligible for OAC	3,885 (6.5)	4,554 (6.4)	1,306 (5.5)	7,348 (19.4)
Eligible–consider OAC	7,089 (11.9)	9,370 (13.1)	1,927 (8.2)	4,349 (11.5)
Eligible–OAC recommended	48,455 (81.6)	57,513 (80.5)	20,357 (86.3)	26,112 (69.1)
HAS-BLED score^a^, n(%)0–1	11,532 (19.4)	14,869 (20.8)	3,355 (14.2)	13,188 (34.8)
2	15,925 (26.8)	20,200 (28.3)	5,388 (22.8)	9,248 (24.5)
≥3	31,972 (53.8)	36,368 (50.9)	14,847 (63)	15,373 (40.7)
Median (IQR)	2 (1 to 2)	2 (1 to 2)	2 (1 to 2)	1 (0 to 2)
**Charlson Comorbidity Index (CCI), n(%)**				
0	18,113 (30.5)	21,141 (29.6)	5,480 (23.2)	12,773 (33.8)
1	9,334 (15.7)	10,862 (15.2)	3,496 (14.8)	5,142 (13.6)
≥2	31,982 (53.8)	39,434 (55.2)	14,614 (62)	19,894 (52.6)
Median (IQR)	2 (0 to 3)	2 (0 to 3)	2 (1 to 4)	2 (0 to 3)
**Frailty index (eFI), n(%)**				
Mostly well	16,967 (28.5)	18,482 (25.8)	4,759 (20.2)	12,591 (33.3)
Mild frailty	25,339 (42.6)	28,935 (40.5)	8,899 (37.7)	12,227 (32.3)
Moderate frailty	13,121 (22.1)	17,002 (23.8)	6,777 (28.7)	8,569 (22.7)
Severe frailty	4,002 (6.7)	7,018 (9.8)	3,155 (13.4)	4,422 (11.7)
**Baseline comorbidity, n(%)**	8,928 (15)	10,992 (15.4)	4,020 (17)	5,429 (14.4)
Cerebrovascular accident/TIA	5,967 (10)	6,935 (9.7)	3,580 (15.2)	2,358 (6.2)
Myocardial infarction	18,415 (31)	21,255 (29.8)	8,803 (37.3)	8,240 (21.8)
Ischaemic heart disease	6,097 (10.3)	6,940 (9.7)	2,983 (12.7)	3,401 (9)
Heart failure	34,61 (5.8)	3,631 (5.1)	1,782 (7.6)	1,708 (4.5)
**Peripheral vascular disease**				
Hypertension	39,717 (66.8)	47,367 (66.3)	15,401 (65.3)	20,304 (53.7)
Diabetes mellitus	12,718 (21.4)	16,588 (23.2)	5,082 (21.5)	6,848 (18.1)
Chronic kidney disease	14,110 (23.7)	15,217 (21.3)	7,010 (29.7)	8,450 (22.4)
Previous bleeding event	7,669 (12.9)	9,853 (13.8)	3,499 (14.8)	5,567 (14.7)
Anaemia	8,488 (14.3)	11,666 (16.3)	4,911 (20.8)	7,664 (20.3)
Liver disease	477 (0.8)	559 (0.8)	215 (0.9)	536 (1.4)
Peptic ulcer	3,211 (5.4)	3,575 (5)	1,410 (6)	2,319 (6.1)
Malignancy	11,641 (19.6)	15,845 (22.2)	5,651 (24)	9,297 (24.6)
Dementia	658 (1.1)	1,983 (2.8)	1,866 (7.9)	2,551 (6.8)
HIV	19 (0.03)	18 (0.03)	8 (0.03)	32 (0.1)
**Baseline treatment** ^**b**^ **, n(%)**				
Antiarrhythmic	175 (0.3)	144 (0.2)	78 (0.3)	58 (0.2)
Antiplatelets^c^	5,598 (9.4)	7,660 (10.7)	1,824 (7.7)	3,742 (9.9)
NSAIDs	5,105 (8.6)	4,957 (6.9)	1,713 (7.3)	2,099 (5.5)
Statins	27,054 (45.5)	33,058 (46.3)	10,186 (43.2)	10,646 (28.2)
CCB	1,482 (2.5)	1,146 (1.6)	560 (2.4)	476 (1.3)
Antibiotics	3,350 (5.6)	3,500 (4.9)	1,410 (5.9)	2,187 (5.8)
Antiepileptic	351 (0.6)	325 (0.5)	227 (1)	207 (0.5)
SSRI/SNRI	3,571 (6)	5,347 (7.5)	2,177 (9.2)	3,218 (8.5)
Triazoles	188 (0.3)	267 (0.4)	102 (0.4)	203 (0.5)
Corticosteroids	4,949 (8.3)	5,758 (8.1)	1,994 (8.5)	3,301 (8.7)
PPI	16,583 (27.9)	23,115 (32.4)	7,605 (32.2)	10,520 (27.8)
Polypharmacy	25,292 (42.7)	37,526 (52.5)	11,525 (48.9)	16,387 (43.3)

BMI, body mass index; CCB, calcium channel blocker; CCI, Charlson comorbidity index; eFI, electronic frailty index; HIV, human immunodeficiency virus; IQR, interquartile range; IMD, index of multiple deprivation; NOAC, non- VKA oral anticoagulant; NSAID, nonsteroidal anti-inflammatory drug; OAC, oral anticoagulant; PPI, proton pump inhibitor; SD, standard deviation; SSRI/SNRI, selective serotonin reuptake inhibitor/selective norepinephrine reuptake inhibitor; TIA, transient ischaemic attack; VKA, vitamin K antagonist.

^a^Modified HAS-BLED score does not include the INR element, and it ranges from 0 to 8.

^b^At least 1 prescription of a certain drug was prescribed within 90 days before diagnosis.

^c^Antiplatelet (P2Y12 inhibitors); clopidogrel, prasugrel, and ticagrelor.

### Factors associated with OAC prescribing

According to the baseline model without interaction terms, practices located in the East of England were associated with higher risk of prescribing aspirin only over no treatment than practices in London [relative risk ratio (RRR) 1.22; 95% CI 1.02 to 1.45] (Table 3). Practices located in deprived areas of IMD 4 were more likely to prescribe aspirin only to no treatment (RRR 1.12; 95% CI 1.01 to 1.25) compared to practices in the least deprived areas (IMD 1), but this association was not significant for practices located in IMD 5. Similar relationships were observed in patient residence area deprivation, with those living in the most deprived areas (IMD 5) were at lower risk of being prescribed OAC (RRR 0.85; 95% CI 0.79 to 0.91) and were at higher risk of being prescribed aspirin only (RRR 1.11; 95% CI 1.02 to 1.21) than patients living in the least deprived areas (IMD 1). Compared to white patients, patients from black, and other ethnic minorities were more likely to receive no treatment than OAC (RRR 0.78; 95% CI 0.65 to 0.94, and RRR 0.76; 0.64 to 0.91, respectively). S14 Table provides the unadjusted comparison for the baseline model.

The deprivation by ethnicity interactions model (S8 Table) also showed that the probability of prescribing OAC was lower for black patients living in deprived areas of IMD 3, 4, and 5; 65% (95% CI 59% to 71%), 66% (95% CI 62% to 71%), and 62% (95% CI 58% to 67%) (S3 Fig and S9 Table). By comparison, the probabilities for white patients in deprivation quintiles 3, 4, and 5 were 69% (95% CI 68% to 70%), 68% (95% CI 68% to 69%), and 66% (95% CI 65% to 67%), respectively. The probability of receiving no treatment was higher for patents residing in the most-deprived areas (IMD 5) in the North West, London, and the East of England region (S4 Fig and S10 Table). The probability of receiving aspirin only was highest for patients residing in the most-deprived areas in the London, East Midlands, East of England, and South Central. Similarly, there was also regional variability in prescribing OAC, as patients residing in the most most-deprived areas had lower probabilities of being prescribed OAC compared to other patients, in the East Midlands, East of England, London, and South Central.

**Table 3:** Results of multivariable analysis evaluating factors associated prescribing of OAC or aspirin only vs. no treatment (reference group) in patients recommended to take OAC.

	OAC		Aspirin only	
	Adjusted RRR* 95% CI	*P* value	Adjusted RRR*95% CI	*P* value
**Region**				
London	Ref			
North East	1.36 (1.19; 1.55)	<0.001	1.21 (1.05; 1.40)	0.010
North West	1.03 (0.90; 1.18)	0.687	0.91 (0.81;1.02)	0.122
Yorkshire and the Humber	1.02 (0.87; 1.19)	0.829	0.98 (0.82; 1.18)	0.822
East Midlands	1.19 (1.03; 1.38)	0.020	1.24 (1.04; 1.47)	0.016
West Midlands	1.26 (1.14; 1.39)	<0.001	0.97 (0.87; 1.07)	0.516
East of England	1.03 (0.86; 1.22)	0.759	1.22 (1.02; 1.45)	0.029
South West	1.42 (1.26; 1.59)	<0.001	1.12 (1.00; 1.26)	0.057
South Central	1.13 (1.01; 1.27)	0.040	1.04 (0.92; 1.17)	0.539
South East Coast	1.19 (1.04; 1.36)	0.014	0.84 (0.73; 0.96)	0.013
**Practice-level IMD**				
1 (least deprived)	Ref			
2	0.99 (0.88; 1.10)	0.819	1.02 (0.91; 1.14)	0.712
3	1.01 (0.91; 1.11)	0.917	1.08 (0.97; 1.20)	0.155
4	0.94 (0.86; 1.04)	0.252	1.12 (1.01; 1.25)	0.028
5 (most deprived)	0.98 (0.86; 1.12)	0.784	1.09 (0.98; 1.23)	0.121
List size (per 1,000)	0.997 (0.985; 1.008)	0.570	0.995 (0.989; 1.001)	0.110
**Patient-level IMD**				
1 (least deprived)	Ref			
2	0.95 (0.90; 1.00)	0.069	1.07 (1.00; 1.14)	0.053
3	0.91 (0.86; 0.97)	0.004	1.09 (1.01; 1.16)	0.017
4	0.92 (0.86; 0.98)	0.017	1.06 (0.98; 1.14)	0.172
5 (most deprived)	0.85 (0.79; 0.91)	<0.001	1.11 (1.02; 1.21)	0.020
**Ethnicity**				
White	Ref			
Black	0.78 (0.65; 0.94)	0.009	0.96 (0.76; 1.21)	0.720
Asian	0.93 (0.81; 1.08)	0.370	0.90 (0.74; 1.10)	0.302
Other	0.76 (0.64; 0.91)	0.002	0.83 (0.65; 1.07)	0.147
**Sex**				
Male	Ref			
Female	1.04 (1.00; 1.07)	0.060	1.02 (0.98; 1.07)	0.362
**Baseline age and BMI**				
18–40	Ref			
41–54	2.21 (1.20; 4.05)	0.011	3.38 (1.08; 10.50)	0.036
55–64	3.32 (1.84; 5.99)	<0.001	3.54 (1.16; 10.83)	0.027
65–74	3.62 (2.01; 6.51)	<0.001	3.11 (1.02; 9.43)	0.045
75–84	3.70 (2.05; 6.67)	<0.001	3.57 (1.18; 10.86)	0.025
≥85	2.05 (1.14; 3.71)	0.017	4.28 (1.41; 12.99)	0.010
BMI	1.046 (1.042; 1.049)	<0.001	1.004 (0.999; 1.008)	0.118
**Disease state and disability**				
Heart failure	0.99 (0.95; 1.04)	0.824	1.01 (0.95; 1.08)	0.695
Cerebrovascular disease/TIA	1.02 (0.98; 1.07)	0.326	1.07 (1.01; 1.14)	0.025
Hypertension	1.11 (1.06; 1.15)	<0.001	0.97 (0.92; 1.01)	0.163
Diabetes	0.86 (0.82; 0.89)	<0.001	0.88 (0.83; 0.92)	<0.001
Rhuematological disease	1.03 (0.96; 1.09)	0.410	0.96 (0.88; 1.04)	0.308
Peptic ulcer	0.84 (0.78; 0.89)	<0.001	0.85 (0.79; 0.93)	<0.001
HIV/AIDS	0.49 (0.19; 1.27)	0.140	0.55 (0.14; 2.23)	0.404
Aneamia	0.74 (0.71; 0.77)	<0.001	0.84 (0.79; 0.88)	<0.001
Dementia	0.52 (0.47; 0.59)	<0.001	1.34 (1.13; 1.58)	0.001
Malignancy	0.74 (0.72; 0.77)	<0.001	0.78 (0.75; 0.82)	<0.001
History of bleeding	0.92 (0.88; 0.96)	<0.001	0.95 (0.89; 1.00)	0.072
Chronic kidney disease	0.93 (0.89; 0.97)	<0.001	1.05 (1.00; 1.10)	0.060
Peripheral vascular disease	1.07 (1.00; 1.14)	0.055	1.28 (1.17; 1.39)	<0.001
Ischaemic heart disease	1.21 (1.16; 1.26)	<0.001	1.52 (1.44; 1.62)	<0.001
Myocardial infarction	1.16 (1.08; 1.23)	<0.001	1.65 (1.52; 1.79)	<0.001
Liver disease	0.58 (0.50; 0.67)	<0.001	0.79 (0.65; 0.96)	0.017
Respiratory disease	1.01 (0.97; 1.05)	0.566	0.92 (0.88; 0.97)	0.001
Parkinsonism	0.93 (0.85; 1.01)	0.087	1.02 (0.91; 1.14)	0.720
Osteoporosis	1.05 (0.99; 1.10)	0.088	1.00 (0.94; 1.08)	0.899
Arthritis	1.08 (1.04; 1.12)	<0.001	0.97 (0.93; 1.02)	0.218
Skin ulcer	0.83 (0.77; 0.89)	<0.001	1.04 (0.96; 1.13)	0.367
History of falls	0.82 (0.78; 0.85)	<0.001	0.95 (0.89; 1.00)	0.063
Dizziness	1.08 (1.04; 1.12)	<0.001	0.99 (0.94; 1.03)	0.594
Fragility fractures	0.90 (0.84; 0.95)	0.001	0.80 (0.74; 0.86)	<0.001
Mobility problems	0.78 (0.74; 0.83)	<0.001	1.02 (0.95; 1.09)	0.571
Cognitive impairment	0.75 (0.67; 0.82)	<0.001	0.65 (0.55; 0.76)	<0.001
Activity limitation	0.82 (0.75; 0.89)	<0.001	0.92 (0.83; 1.02)	0.094
Visual impairment	0.98 (0.94; 1.02)	0.305	1.04 (0.99; 1.10)	0.112
Require care	0.88 (0.81; 0.96)	0.004	1.02 (0.91; 1.14)	0.779
Socially vulnerable	0.92 (0.87; 0.97)	0.002	0.84 (0.79; 0.90)	<0.001
Housebound	0.72 (0.68; 0.76)	<0.001	1.06 (0.99; 1.12)	0.076
**Baseline drug use**				
Polypharmacy	0.90 (0.86; 0.95)	<0.001	0.77 (0.73; 0.82)	<0.001
Antibiotics	0.89 (0.83; 0.96)	0.002	0.98 (0.89; 1.07)	0.591
Antiepileptics	0.90 (0.73; 1.10)	0.287	1.58 (1.24; 2.00)	<0.001
Calcium channel blockers	1.35 (1.18; 1.56)	<0.001	1.65 (1.41; 1.94)	<0.001
Corticosteroids	0.81 (0.76; 0.86)	<0.001	0.85 (0.79; 0.92)	<0.001
Antiplatelets	0.76 (0.71; 0.80)	<0.001	0.43 (0.40; 0.47)	<0.001
SSRI/SNRI	0.86 (0.81; 0.91)	<0.001	1.10 (1.03; 1.18)	0.008
Statins	1.64 (1.57; 1.70)	<0.001	1.45 (1.38; 1.53)	<0.001
Trizoles	0.68 (0.55; 0.84)	<0.001	0.78 (0.58; 1.05)	0.103
PPI	1.02 (0.98; 1.06)	0.344	1.00 (0.95; 1.05)	0.871
NSAIDs	1.12 (1.05; 1.20)	0.001	1.35 (1.24; 1.47)	<0.001
**Smoking status**				
Nonsmoker/ Ex-smoker	Ref			
Current smoker	0.81 (0.77; 0.84)	<0.001	0.95 (0.90; 1.00)	0.057
**Alcohol consumption status**				
Nondrinker	Ref			
Light drinker	1.14 (1.07; 1.21)	<0.001	1.05 (0.97; 1.14)	0.249
Former drinker	1.03 (0.91; 1.18)	0.628	1.18 (1.02; 1.37)	0.027
Moderate drinker	1.13 (1.08; 1.19)	<0.001	0.98 (0.92; 1.05)	0.626
Heavy drinker	0.95 (0.88; 1.02)	0.158	0.85 (0.78; 0.94)	0.001

*Adjusted for age, sex, comorbidities, socioeconomic status, baseline treatment, frailty, bleeding risk factors, and takes in account clustering by general practice.

BMI, body mass index; CCB, calcium channel blocker; CI, confidence interval; HIV, human immunodeficiency virus; IMD, index of multiple deprivation; NOAC, non- VKA oral anticoagulant; NSAID, nonsteroidal anti-inflammatory drug; OAC, oral anticoagulant; PPI, proton pump inhibitor; SSRI/SNRI, selective serotonin reuptake inhibitor/selective norepinephrine reuptake inhibitor; TIA, transient ischaemic attack.

The interaction effect between ethnicity and practice-level IMD was examined (S11 Table) and showed that black patients or those from other ethnicities had overall higher probabilities of no treatment across all practice-level deprivation quintiles, compared to patients of white ethnicity (S5 Fig and S12 Table). The probability of prescribing OAC was lower for black patients who were registered in practices in deprivation quintiles 3, 4, and 5, 61% (95% CI 53% to 68%), 64% (95% CI 59% to 69%), and 64% (95% CI 59% to 68%), respectively. By comparison, the probabilities for white patients in deprivation quintiles 3, 4, and 5 were 70% (95% CI 69% to 71%), 68% (95% CI 67% to 69%), and 68% (95% CI 67% to 70%), respectively. The probability of receiving aspirin only was higher for patients registered in practices in the most-deprived areas (IMD 4 and 5) in the East of England; 20% (95% CI 15% to 25%) and 26% (95% CI 19% to 33%) (S6 Fig and S13 Table). Additionally, the probability of OAC prescribing was the lowest for patients registered in practices in the most-deprived areas in the East of England; 58% (95% CI 49% to 66%).

According to the baseline model without interaction terms (Table 3), the association between age and aspirin-only prescribing gets stronger with increasing age such as patients with age ≥85 years who have the highest risk of being prescribed aspirin only (RRR 4.28; 95% CI 1.41 to 12.99) compared to patients aged 18 to 40 years. Patients with dementia were less likely to receive OAC and more likely to receive aspirin only. Although cardiovascular conditions such as ischaemic heart disease and myocardial infarction was associated with OAC prescribing, it was also associated with prescribing of aspirin only. Many other clinical conditions were associated with lower risk of prescribing OAC such as diabetes, peptic ulcer, anaemia, malignancy, liver disease, cognitive impairment, bleeding history, history of falls, and also some of the eFI components like polypharmacy, mobility problems, limited activity, fragility fractures, patients who are housebound, and who require care.

In the sensitivity analysis we conducted using binomial logistic regression, the aspirin-only group was combined with the no-treatment groups (S15 Table), and overall, the model showed similar associations between patient factors and OAC prescribing to that observed in the main model (Table 3). Such as practice-level IMD and patient-level IMD, where most deprived quintile (IMD 5) was associated with lower risk of OAC prescribing compared to the least deprived quintile (IMD 1) (S15 Table). Compared to white ethnicity, patients from black and other ethnic minorities were less likely to be prescribed OAC. While clinical conditions overall showed similar associations with OAC prescribing to that observed in the main model, myocardial infarction was associated with not prescribing OAC in the sensitivity analysis, as opposed to that observed in the main model where it was associated with higher risk of prescribing OAC or aspirin only. Moreover, unlike the main model, some of the eFI components in the sensitivity analysis such as cognitive impairment, activity limitation, socially vulnerable patients, patients who require care, or who are housebound were found to be more likely to receive OAC (S15 Table).

### Trends in OAC prescribing by ethnicity and IMD quintile over time

**Fig 6:**
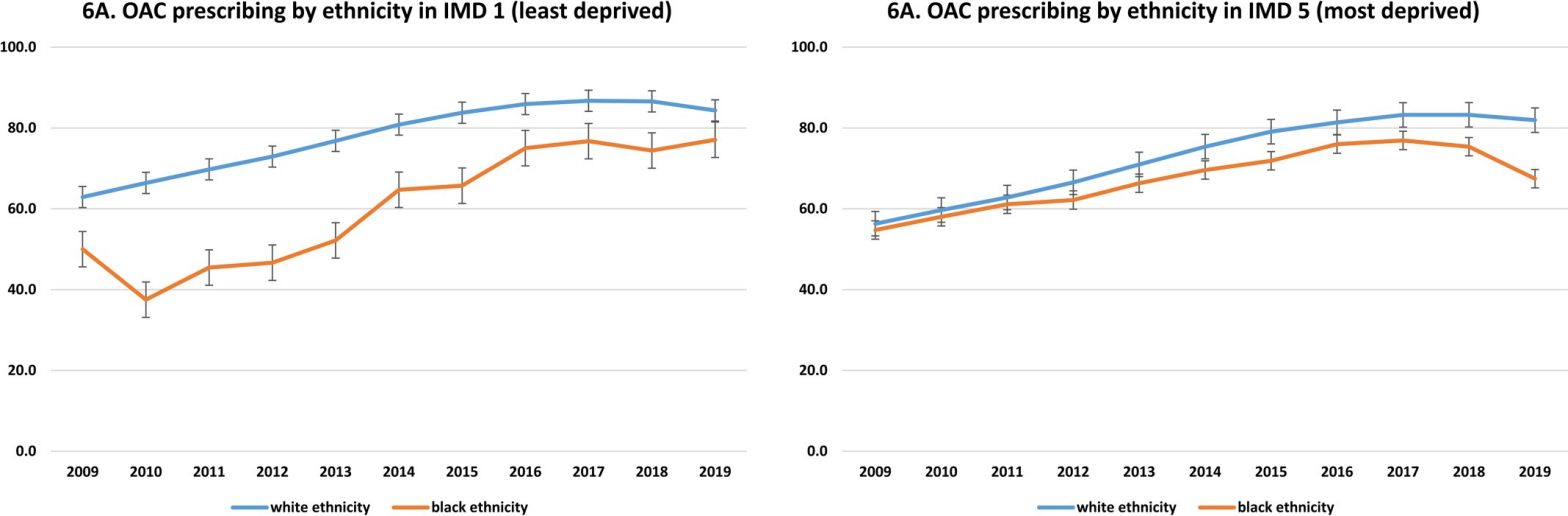
Trend in OAC prescribing over time by ethnicity and deprivation quintile, estimated as proportions and 95% CI. CI, confidence interval; IMD, index of multiple deprivation; OAC, oral anticoagulant.

Fig 6 illustrates the changing trend of OAC prescribing over time by ethnicity and IMD quintile among patients with NVAF who are eligible for OAC. The proportion of patients from white ethnicity living in the least deprived areas (IMD 1) who received OAC have increased from 62.9% (95% CI: 61.3; 64.5) in 2009 to 84.3% (95% CI: 83.9; 84.8) in 2019. While the proportion of patients from black ethnicity living in the least deprived areas (IMD 1) who received OAC have increased from 50% (95% CI: 5.8; 94.2) in 2009 to 77% (95% CI: 63.1; 86.9) in 2019. On the other hand, among patents living in the most deprived areas (IMD 5) the proportion of white patients who received OAC have increased from 56.3% (54.2; 58.4) in 2009 to 81.9% (81.3; 82.6) in 2019, whereas the proportion of black patients who received OAC have increased from 54.8% (95% CI: 39.7; 69.0) in 2009 to 76.9% (71.9%; 81.3%) in 2017, before declining to 67.4% (95% CI: 62.3; 72.2) in 2019.

## Discussion

This large retrospective cohort study shows that NVAF incidence rates rose by almost a quarter in England between 2009 and 2019 and were markedly higher in males than in females. For patients with an indication for anticoagulant therapy, prescribing increased by one-third and underprescribing fell by more than half over the study period. Underprescribing was largely associated with specific practice and patient baseline factors. At the practice level, there were variations in anticoagulation prescribing relating to patient ethnicity and local area socioeconomic deprivation, with black patients in deprived areas more likely to be prescribed aspirin only or no treatment. Regional variation was also apparent, with the lowest rates of anticoagulation in the East of England. Patient-level data showed that patients from black or other ethnic minorities and patients living in more deprived areas were less likely to be prescribed OACs. Additionally, our findings have emphasised the association between different comorbidities and underprescribing of OACs. These findings provide further evidence to suggest that patients at higher risk of stroke due to their baseline comorbidities and who are more likely to benefit from anticoagulation are not receiving appropriate treatment.

The proportion of patients with NVAF who were eligible for OAC and who were prescribed OAC increased substantially between 2011 and 2015. This increase in OACs prescribing may be related to a change in ESC guidelines in 2010, which recommended OACs for all patients with AF at moderate risk to high risk of stroke rather than antiplatelet therapy [38]. This was followed by a 2012 update recommending avoidance of aspirin prescribing in low-stroke risk patients [16]. Increases in OAC prescribing following such guideline changes have previously been reported in studies from the UK and the United States [10,39]. Our results showed increasing rates of NOACs prescribing as first treatment after NVAF diagnosis starting in 2011, while prescribing of VKAs continued to decline from 2013 to 2019. A possible explanation for this shift in prescribing from VKAs to NOACs is reporting from several large randomised trials that investigated the noninferiority of NOACs compared to VKAs [6–8], which were followed by the approval of the first NOACs (Dabigatran) in NVAF by the European Union in 2011.

Despite this increase in OACs prescribing, we report important racial and socioeconomic inequalities in the prescribing of OACs, with low socioeconomic status and black or other non-white ethnicities associated with the prescription of aspirin only or no treatment compared to white patients or those with higher socioeconomic status. Moreover, previous studies showed that even in high-income countries with universal healthcare systems, socioeconomic inequality in OACs prescribing exists. A nationwide cohort study in Denmark reported that patients with low income, low education, and living alone were associated with less chances of being prescribed OACs [40]. Since the National Health System in England provides free publicly funded healthcare, it is less likely that financial limitations are a major factor for this socioeconomic inequality, but substantial nonfinancial barriers in seeking and benefiting from healthcare remain and drive inequalities in diagnosis, treatment, and outcomes [41]. A study from the UK that used data from the CPRD found that individuals living in areas with high socioeconomic deprivation are at greater risk of developing AF and AF fatality, suggesting there is a discrepancy in AF diagnosis and mortality by deprivation [42]. Another study that focused on socioeconomic disparities in first stroke incidence and quality of care found that compared with patients from the least deprived areas, all patients with lower socioeconomic status were less likely to receive anticoagulation for AF at the time of discharge from secondary care [43]. Although our findings showed that this inequality in OAC prescribing between white and black patients is getting narrower over time for patients residing in the least deprived areas, it is not the same case for patients residing in the most deprived areas. As this gap in OAC prescribing between white and black patients is getting wider especially after 2017. According to a recent study that used data from the USA showed that despite that OAC initiation overall increased significantly over time, racial/ethnic disparities in OAC prescribing was persistent over time.

Social and personal factors that are part of the eFI and we have captured in our prediction model may partially explain this, such as patients requiring additional care, being housebound, or being socially vulnerable. Previous work has examined the association between ethnicity and OAC prescribing in the US [44,45], but little is known in the UK. A local cross-sectional study found that there were no clear differences in warfarin prescribing by ethnic group, using data from general practice records from south and east London from 2008 to 2011 [46]. Our findings differed, indicating strong inequalities in OAC prescribing by ethnicity in England, which could be explained by the larger national sample, regional variation, and changes over time.

In this study, several comorbidities were identified to be associated with lower risk of OAC prescribing. Dementia was a strong predictor for lack of anticoagulation or prescribing of aspirin only. Although, an earlier study that used data from the CPRD found that dementia was one of the factors associated with prescribing of warfarin in specific subgroups [47]. Our findings are in line with a more recent data derived from the UK, which found that 64% of patients with NVAF and dementia and who are at risk of stroke do not receive OACs in the year following NVAF diagnosis [48]. Guidelines recommend prescribing OACs for NVAF with dementia or cognitive impairment, unless adherence cannot be ensured by the caregiver [49,50]. Despite these recommendations, OACs are greatly underprescribed for patients with dementia [51]. Although nonadherence can impact the treatment decision in patients with dementia [48], other factors may also influence this such as frailty and comorbidities [20]. We have also found that comorbidities that increase patients risk for bleeding were predictors for OACs underprescribing such as anaemia, history of bleeding, peptic ulcer, and comorbidities that cause thrombocytopenia like liver disease and also malignancy [50].

Our findings revealed that patients with history of ischaemic heart disease are more likely to receive aspirin only, although antiplatelet monotherapy is not sufficient for stroke prevention and could potentially be harmful [52]. Similarly, patients who have been prescribed antiplatelet (P2Y12 inhibitors) are more likely to receive no treatment or aspirin only. These findings reflect the challenges in the management of patients with NVAF and a history of ischaemic heart disease and suggest that this subgroup of patients are not treated with evidence-based therapy such as dual antithrombotic therapy (NOACs or VKAs, and P2Y12 inhibitors), or in some cases at least a short course of triple therapy by adding aspirin (e.g., ≤1 week) would be desirable in some AF patients after a recent acute coronary syndrome or undergoing percutaneous coronary intervention [50].

Our study has several important strengths. First, we used 2 different data sources, the CPRD GOLD and Aurum databases, which enabled inclusion of a large sample that is representative of the population of England [53,54]. Second, the selected study period was contemporary and so relevant to current clinical practice and captured the changes in stroke prevention practice, ranging from the period of NOACs approval. Third, we applied conservative inclusion criteria to only include newly diagnosed NVAF patients to increase the validity of the presented results. Fourth, the inclusion of interaction terms in our analysis enabled the identification of ethnic and regional inequalities in OACs prescribing and their association with socioeconomic deprivation, and these findings demonstrate the variation in NVAF care, which could potentially influence clinical outcomes and indicate the need for initiatives to ensure uniform high-quality care for patients with NVAF.

Nevertheless, our study is subject to a number of limitations. First, when we estimated the incidence rate of NVAF by age group, we observed a declining trend in prevalence in the oldest groups in our cohort, which was contrary to expectations. Upon closer inspection, we could not identify the reason with certainty, but it may be driven by poor recording of exits from the database in the earlier years, which greatly improved over time. Second, although we intended in this study to include newly diagnosed patients with NVAF with no previous history of valve disease, recent anticoagulant therapy, or cardioversion, this might have restricted the generalisability of the study results for patients suffering from a long history of NVAF or those with more complex disease state. Third, our findings are dependent on accurate recording from the health professionals. Lack of event recording would result in a false negative classification of a certain event and therefore could potentially bias our findings. However, considering the clinical significance of the event, we would expect recording for these events to be relatively complete. Fourth, although we included many clinical and socioeconomic factors in the regression model to explore factors associated with underprescribing of OACs, there is inevitable residual confounding in observational studies due to lack of data on certain factors that could be associated with underprescribing of OACs that we did not account for such as physicians’ attitude in prescribing OAC. Although it was not feasible to investigate physicians’ preference or their viewpoints on prescribing OAC, we acknowledge that in some situations, underprescribing of OAC could be justifiable given the contraindications the patient might have and that physicians consider old age and the associated comorbidities and the increased potential for bleeding as potential barriers to optimise anticoagulant therapy [55]. However, in the context of our key findings, it seems unlikely that the inequality in prescribing OAC that is associated with ethnicity or socioeconomic deprivation is driven by contraindications. Fifth, drug data were analysed using primary records only, and in the case if a patient was managed exclusively in secondary care, then we would incorrectly define them as untreated due to lack of prescription data in secondary care. There is also the possibility of over-the-counter usage of aspirin that we could not capture in our analysis; however, the amount of over-the-counter use of aspirin is likely to be low as reported previously in a CPRD study that compared prescription records to patient self-report and found that the majority of chronic aspirin therapy was captured by CPRD prescription records [56]. Sixth, the CPRD includes prescription data alone, and, hence, we have no information on administration or adherence. Seventh, there is a chance that the bleeding risk was underestimated because we adopted a modified HAS-BLED score that did not include labile INR as risk factor, due to the inconsistency of INR recording within the CPRD [35].

The annual incidence of NVAF has increased between 2009 and 2019 in England and was more common among elderly patients and in males. Similarly, the proportion of patients with NVAF receiving evidence-based anticoagulation therapy has increased over time, but there are still many patients at risk for stroke who are not receiving anticoagulants. Clinical and sociodemographic factors play a role in the underprescribing of OACs, including comorbidities such as dementia, and patient’s ethnicity and socioeconomic status. Addressing these inequalities through equitable interventions to improve OAC prescribing could substantially improve AF outcomes, preventing stroke events and reducing mortality. Future studies should continue to investigate potential causes for these inequalities.

## Supporting information

S1 Protocol Epidemiology of anticoagulants prescribing in nonvalvular atrial fibrillation patients in England: a cohort study exploring disease incidence and anticoagulants prescribing trends, comparing clinical outcomes, and risk of recurrent bleeding events. (PDF)

S1 RECORD-PE Checklist The RECORD statement for pharmacoepidemiology (RECORD-PE) checklist of items, extended from the STROBE and RECORD statements, which should be reported in noninterventional pharmacoepidemiological studies using routinely collected health data. (DOCX)

S1 Fig Sex-specific annual standardised incidence rates per 10,000 patients and 95% CI from practices that contributed for 11 years or less. (PDF)

S2 Fig Sex-specific annual standardised incidence rates per 10,000 patients and 95% CI from practices that contributed throughout the study period (for 11 years). (PDF)

S3 Fig Predictive probabilities and 95% CI of prescribing OAC, aspirin only, or no treatment based on ethnicity and patient-level IMD. (PDF)

S4 Fig Predictive probabilities and 95% CI of prescribing OAC, aspirin only, or no treatment based on practice region and patient-level IMD. (PDF)

S5 Fig Predictive probabilities and 95% CI of prescribing OAC, aspirin only, or no treatment based on practice-level IMD and ethnicity. (PDF)

S6 Fig Predictive probabilities and 95% CI of prescribing OAC, aspirin only, or no treatment based on practice-level IMD and practice region. (PDF)

S1 Table Characteristics of included general practices. (PDF)

S2 Table Overall and sex-specific annual standardised NVAF incidence rates per 10,000 patients and 95% CI from practices that contributed data throughout the study period (for 11 years). (PDF)

S3 Table Sex-specific annual standardised incidence rates per 10,000 patients and 95% CI from practices that contributed for 11 years or less. (PDF)

S4 Table Sex-specific annual standardised incidence rates per 10,000 patients and 95% CI from practices that contributed throughout the study period (for 11 years). (PDF)

S5 Table Proportions of patients prescribed OACs (VKA or NOAC), aspirin only, or no treatment for patients eligible for OAC. (PDF)

S6 Table Proportion of patients prescribed first treatment after NVAF diagnosis stratified by the type of drug initiated, from 2009 to 2019. (PDF)

S7 Table Proportions of patients prescribed OACs (VKA or NOAC), aspirin only, or no treatment among patients not eligible for OAC. (PDF)

S8 Table Results of multivariable analysis with interaction terms evaluating factors associated prescribing of OAC or aspirin only vs. no treatment (reference group) in patients recommended to take OAC. (PDF)

S9 Table Marginal analysis for the predictive probability of prescribing OAC, aspirin only, or no treatment based on ethnicity and patient-level IMD. (PDF)

S10 Table Marginal analysis for the predictive probability of prescribing OAC, aspirin only, or no treatment based on practice region and patient-level IMD. (PDF)

S11 Table Results of multivariable analysis with interaction terms evaluating factors associated prescribing of OAC or aspirin only vs. no treatment (reference group) in patients recommended to take OAC. (PDF)

S12 Table Marginal analysis for the predictive probability of prescribing OAC, aspirin only, or no treatment based on ethnicity and practice-level IMD. (PDF)

S13 Table Marginal analysis for the predictive probability of prescribing OAC, aspirin only, or no treatment based on practice region and practice-level IMD. (PDF)

S14 Table Results of the unadjusted univariate analysis evaluating factors associated prescribing of OAC or aspirin only vs. no treatment (reference group) in patients recommended to take OAC. (PDF)

S15 Table Sensitivity analysis using multivariable binomial logistic regression to investigate the association between patient characteristics and prescribing of oral anticoagulants. (PDF)

## Abbreviations

AF: atrial fibrillation
BMI: body mass index
BNF: British National Formulary
CCI: Charlson comorbidity index
CI: confidence interval
CPRD: Clinical Practice Research Datalink
eFI: electronic frailty index
GP: general practitioner
IMD: Index of Multiple Deprivation
IQR: interquartile range
NOAC: non-vitamin K antagonist oral anticoagulant
NSAID: nonsteroidal anti-inflammatory drug
NVAF: nonvalvular atrial fibrillation
OAC: oral anticoagulant
PYR: person-years
QOF: Quality and Outcomes Framework
RRR: relative risk ratio
SNRI: selective norepinephrine reuptake inhibitor
SSRI: selective serotonin reuptake inhibitor
TIA: transient ischemic attack
VKA: vitamin K antagonist
