# Supplementary material for: Incidence of nonvalvular atrial fibrillation and oral anticoagulant prescribing in England, 2009 to 2019: A cohort study
Source: PLoS Med. 2022 Jun 7;19(6):e1004003. doi: 10.1371/journal.pmed.1004003 (PMC9173622; doi:10.1371/journal.pmed.1004003)
Supplement: S1 Protocol — (PDF) [file pmed.1004003.s001.pdf]

1

General information

Protocol reference Id
20_198

Study title
Epidemiology of anticoagulants prescribing in non-valvular atrial fibrillation patients in England: a cohort study exploring disease incidence and anticoagulants prescribing trends, comparing clinical outcomes, and risk of recurrent bleeding events

Research Area

Disease Epidemiology

Pharmacoepidemiology

Does this protocol describe an observational study using purely CPRD data?
No

Does this protocol involve requesting any additional information from GPs, or contact with patients?
No

Research team

Role	Chief Investigator
**Title**	Professor in Data Science & Health Services Research
**Full name**	Evangelos Kontopantelis
**Affiliation/organisation**	University of Manchester
**Email**	e.kontopantelis@manchester.ac.uk
**Will this person be analysing the data?**	Yes
**Status**	Confirmed

Role	Corresponding Applicant
**Title**	postgraduate research student
**Full name**	Alyaa Ajabnoor
**Affiliation/organisation**	University of Manchester
**Email**	alyaa.ajabnoor@postgrad.manchester.ac.uk
**Will this person be analysing the data?**	Yes
**Status**	Confirmed

Role	Collaborator
**Title**	Professor of Pharmacoepidemiology
**Full name**	Darren Ashcroft
**Affiliation/organisation**	University of Manchester
**Email**	darren.ashcroft@manchester.ac.uk
**Will this person be analysing the data?**	No
**Status**	Confirmed

Role	Collaborator
**Title**	Professor of Cardiology
**Full name**	Mamas Mamas
**Affiliation/organisation**	Keele University
**Email**	m.mamas@keele.ac.uk
**Will this person be analysing the data?**	No
**Status**	Confirmed

**Role**	Collaborator
**Title**	Research Associate in Pharmacoepidemiology
**Full name**	Rosa Parisi
**Affiliation/organisation**	University of Manchester
**Email**	rosa.parisi@manchester.ac.uk
**Will this person be analysing the data?**	Yes
**Status**	Confirmed

**Role**	Collaborator
**Title**	Presidential Fellow (Research)
**Full name**	Salwa Zghebi
**Affiliation/organisation**	University of Manchester
**Email**	salwa.zghebi@manchester.ac.uk
**Will this person be analysing the data?**	Yes
**Status**	Confirmed

3 Access to data

Sponsor
University of Manchester

Funding source for the study
Is the funding source for the study the same as Chief Investigator's affiliation?
Yes
Funding source for the study
University of Manchester

Institution conducting the research
Is the institution conducting the research the same as Chief Investigator's affiliation?
Yes
Institution conducting the research
University of Manchester

Method to access the data
Indicate the method that will be used to access the data
Institutional multi-study licence
Is the institution the same as Chief Investigator's affiliation?
Yes
Institution name
University of Manchester

Extraction by CPRD
Will the dataset be extracted by CPRD
No

Multiple data delivery
This study requires multiple data extractions over its lifespan
No

Data processors

4

Information on data

Primary care data

CPRD GOLD

CPRD Aurum

Do you require data linkages
Yes

Patient level data

HES Admitted Patient Care

ONS Death Registration Data

NCRAS data

Covid 19 linkages

Area level data
Do you require area level data?
Yes

Practice level (UK)

Practice Level Index of Multiple Deprivation

Patient level (England only)

Patient Level Index of Multiple Deprivation

Withheld concepts

Are withheld concepts required?
No

Linkage to a dataset not listed

Are you requesting a linkage to a dataset not listed?
No

Patient data privacy

Does any person named in this application already have access to any of these data in a patient identifiable form, or associated with an identifiable patient index?
No

5

Protocol information

Lay Summary

Despite improvements in the assessment of the risks of stroke and bleeding in people with atrial fibrillation – a common type of irregular heartbeat, many people are still not optimally treated with anticoagulants (blood thinners to prevent blood-clotting). It is believed that this may be due to overestimating patient's bleeding risk during anticoagulation therapy. The consequences of lack of anticoagulation could put patients at higher-risk for stroke or venous thromboembolism (blood clot), which are associated with long-term disability and death. Using UK primary-care data and hospitalization records from the Clinical Practice Research Datalink, we will determine how commonly atrial fibrillation is diagnosed in England and how it is treated. We aim to evaluate the factors that influence under-prescribing of anticoagulants in patients with atrial fibrillation in general and in a sub-group of patients with cancer, and explore their stroke and bleeding risk and concomitant therapy. We will use statistical methods to compare the risk of stroke, bleeding, or death among patients who received and did not anticoagulant therapy. We will separately examine patients who experienced severe bleeding due to anticoagulants, and whether they continued on anticoagulants after their bleeding episode. This will allow the identification of factors related to these prescribing decisions and how anticoagulants influence patients' clinical-outcomes. We will also assess and compare their risk for a stroke or another bleeding event using different risk-assessment tools. We anticipate that our findings will help in improving prescribing decisions of anticoagulant therapy in patients with atrial fibrillation at risk for stroke.

Technical Summary

Background and Objective:

Atrial fibrillation (AF) significantly increase the risk of ischemic stroke. The optimal use of oral anticoagulants (OACs) and control of stroke-related risk factors, is proven to substantially reduce stroke risk. However, some clinicians may not prescribe OACs to AF patients at-risk for stroke, as they believe that the risk of bleeding outweighs the benefit of anticoagulation. We aim to assess and compare the impact of prescribing anticoagulants by examining OACs prescribing in a cohort of non-valvular atrial fibrillation (NVAF) patients in England.

Methods and Data analysis:

The study comprises of three phases; first an epidemiological study that aims to delineate incidence of NVAF from the CPRD GOLD and Aurum databases between 2009 and 2019. Prescribing of OACs will be assessed for all patients with NVAF and in a sub-group with cancer diagnosis and relevant data will be extracted to identify potential predictors of OACs prescribing and further assessment using multivariable logistic-regression modelling. The second phase will be a comparative cohort study of incident NVAF patients who were classified as "OAC not prescribed" in phase one, by using a propensity score matching method to match each patient not prescribed OACs to up to 5 comparable patients treated with OACs. Then we aim to compare stroke, bleeding, and all-cause mortality associated with OACs prescribing versus no OACs. The third phase will focus on patients who developed major bleeding events in phase two while taking OACs. We aim to explore the variability of OACs prescribing after bleeding across patient groups and the different predictors for OACs resumption. We will explore the rate of bleeding reoccurrence, stroke, and mortality, and how they relate to OACs prescribing and timing of restarting OACs. We will assess and compare patients' risk for stroke and bleeding reoccurrence after the bleeding event using different risk-assessment scores.

Outcomes to be measured

Throughout the different phases of this project we aim to explore the following outcomes:

- Phase 1 – The primary outcome is the prescription of OAC drugs including; vitamin K antagonists (VKAs) (acenocoumarol, phenindione or warfarin), or non-vitamin K oral anticoagulants (NOACs) (dabigatran, rivaroxaban, apixaban, or edoxaban).
- Phase 2 - The primary outcome is ischaemic stroke and other thromboembolic events, or the occurrence of major bleeding (MB) defined according to the International Society on Thrombosis and Haemostasis (ISTH) criteria as (1) clinically overt bleeding accompanied by a drop-in haemoglobin level of least 2 g/dl and/or (2) transfusion of at least 2 units of packed red cells, occurring at a critical site (i.e., intracranial, intraspinal, intraocular, pericardial, intra-articular, intramuscular, and retroperitoneal) and/or (3) fatal bleeding.1 Secondary outcomes will include clinically relevant non major bleeding (CRNMB) that did not satisfy the ISTH criteria and led to hospitalization, medical or surgical treatment, or led to discontinuation of OACs.
- Phase 3 – The primary outcomes include the reoccurrence of MB events or CRNMB for patients who did or did not continue on OACs, the rate of stroke and other thromboembolic events, cardiovascular related mortality, and all-cause mortality. The secondary outcome is the prescription of any OACs after the occurrence of the MB events. We aim to explore the timing of reinitiating OACs from the indexed MB event and weather patients have continued the same OAC before the index haemorrhage event, were switched to another OACs or received lone antiplatelet therapy.

All outcomes will be identified over the course of follow-up through the identification of corresponding Read/SNOWMED codes in patients' electronic health records. Hospitalization secondary to stroke or bleeding are to be identified through linkage to HES APC using the 10th revision of the International Classification of Diseases (ICD-10). ONS mortality data will be used to ascertain specific causes of death (i.e. bleeding related, cardiovascular, or all-cause death) according to ICD-10 codes.

Objectives, specific aims & rationale

Objectives:
To longitudinally examine the prescribing patterns and predictors of OACs prescribing and to evaluate the clinical outcomes of the under prescription of OACs in NVAF patients.

Specific aims:
1. To determine the incidence of NVAF diagnosis in England from the year 2009 to 2019.
2. To determine the proportions of patients with NVAF and those with NVAF and cancer who were prescribed OACs, antiplatelet alone or no treatment.
3. To examine the temporal trends of prescribing different types of OACs (i.e. VKAs and NOACs), and assess the factors associated with not prescribing OACs including patient demographics, socioeconomic status, history of cancer and other comorbidities, stroke and bleeding risk.
4. To assess the effect of OACs prescribing vs. no OACs on the clinical outcomes (stroke, bleeding, and death) in a comparative cohort of NVAF patients eligible for anticoagulation after controlling for patients' comorbid conditions, frailty, age. and antiplatelets use.
5. To assess the rate of clinical outcomes (i.e. bleeding reoccurrence, stroke, and mortality) after MB events and how they relate to OACs resumption and the timing of restarting OACs after the MB event.
6. To explore the variability across patient groups regarding OACs resumption after MB events and weather patients have continued on the same OAC before the MB event, switched to another OACs or received lone antiplatelet therapy.
7. To assess and compare patients' risk for stroke and bleeding reoccurrence using HAS-BLED, CHA2DS2-VASC, HEMORR?HAGES and GARFIELD-AF risk assessment scores. at the time of the indexed major bleeding event.

Rationale:
Achieving these specific aims will allow the identification of predictors for the under prescription of OACs in NVAF who are eligible for anticoagulation therapy. By further comparing the clinical outcomes between OACs treated and not treated individuals we will be able to determine the characteristics of patients who are still more likely to benefit from receiving anticoagulation therapy. The outcomes of restarting OACs after major bleeding events and their associated benefits or risks are still not well known and the proper timing of restarting OACs after major bleeding events is still not certain and further studies are needed to evaluate the effect of different time intervals to resume OACs. Moreover, available risk assessment scores such as the HAS-BLED, CHA2DS2-VASC, and HEMORR?HAGES are used to assess patients' risk for stroke and bleeding when initiating OACs, but it is unclear if these scores or the new GARFIELD-AF score could be used to inform decision-making for restarting OACs after major bleeding events.

Study background

In 2017, it was estimated that 1.4 million people in England have atrial fibrillation (AF), which is equal to 2.5% of the population.2 One of the main therapeutic goals in AF is to reduce the risk of thrombo-embolism and stroke, which can be achieved by using oral anticoagulants (OACs).3 Previous observational studies in the UK that used primary care data such as the CPRD, have found a steady increase in the prescribing of OACs throughout the past decade.4–6 This observed increase in OACs prescribing was further explained by the introduction and adoption of non-vitamin K oral anticoagulants (NOACs). This increasing uptake of OACs also corresponds to a change in the European Society of Cardiology (ESC) guidelines that recommended the treatment of moderate-risk to high risk AF patients with OACs rather than antiplatelets,7 as well as a change in the Quality and Outcome Framework (QOF) to incentivize prescribing of anticoagulants.4 However, despite this encouraging increase in OACs initiation in AF patients, evidence from an observational study using CPRD have showed that the proportion of patients not receiving any anticoagulation therapy from the year 2012 to 2016 remained the same, at around 15% of patients with non-valvular atrial fibrillation (NVAF).6

The decision to start OAC therapy in AF patients mainly depends on balancing patient's risk for ischemic strokes against their risk for bleeding.7 Current guidelines recommend estimating stroke

risk in AF patients using the CHA2DS2-VASc score [congestive heart failure, hypertension, age 75 years, diabetes mellitus, prior stroke or transient ischemic attack or thromboembolism, vascular disease, age 65 to 74 years, and sex category].7–9 OACs should be considered for patients with 1 or more non-sex CHA2DS2-VASc stroke risk factors. Patients with high-risk for stroke are those with CHA2DS2-VASc 2 in males or 3 in females. While bleeding risk assessment can be achieved by using HAS-BLED score [hypertension, abnormal kidney or liver function, stroke, history of bleeding, labile INR, elderly (> 65 years), and drugs/alcohol concomitantly].3 However, a high bleeding risk score (HAS-BLED 3) should generally not lead to holding or not prescribing OACs. Rather, bleeding risk factors if modifiable should be identified and corrected such as hypertension, interacting medications, or labile international normalized ratio (INR) for patients on warfarin.7

According to previous studies, elderly patients (>85 years) are less likely to be prescribed anticoagulation therapy, this observation is well documented in several studies,10–16 and is mainly attributed to the overestimation of bleeding risk.17–19 Moreover, elderly patients with high risk for stroke and those with coronary artery disease are more likely to be prescribed only one antiplatelet therapy,6 despite the 2016 ESC guidance which state that antiplatelet monotherapy should not be used for stroke prevention in AF patients.6 Observational studies showed that under prescribing of OACs in high-risk AF patients is associated with higher rates of stroke rates and death.20,21 OACs prescribing is largely based on physician’s assessment of benefit (reducing stroke risk) versus risk (potential for bleeding),22 while considering many patients related factors. An example to this, is patients with AF and cancer since their risks of venous thromboembolism (VTE) and of non-cardio embolic stroke are higher than in the general population and may have to factor into the decision for some cancer subtypes,23,24 and also the bleeding risk is often much higher in patients with cancer due to coagulopathies, thrombocytopenia, anaemia that increase the propensity towards bleeding events. This illustrate the risk–treatment paradox previously reported in AF management that patients at higher risk of stroke and who are more likely to benefit from OACs are not receiving appropriate treatment, due to their bleeding risk.11 A comparative evaluation of physicians’ decisions to prescribe OACs or not in high risk AF patients and their associated clinical outcomes could yield more informative, actionable conclusions if it provides a focused evaluation of patients characteristics and co-morbidities to identify the type of high-risk AF patients who would benefit from OACs without experiencing bleeding events.

Earlier studies have shown that anticoagulation therapy based on guideline risk stratifications is associated with a 60%–70% reduction in thromboembolic events and mortality.25 However, there is still room to improve risk stratification of patients with AF and anticoagulation. Recently, the Global Anticoagulant Registry in the FIELD-Atrial Fibrillation (GARFIELD-AF) model was developed and it allows the simultaneous prediction of stroke, bleeding, and mortality.26 This model is not a risk score that categories patients into risk groups, but instead it provides a risk prediction on a continuous scale. After external validation of the model in a Danish cohort, the GARFIELD-AF model was superior to CHA2DS2VASc in predicting stroke/SE and comparable with HAS-BLED for predicting major bleeding.27 Due to the novelty of this risk prediction tool, further studies are needed to demonstrate its ability to accurately stratify AF patients based on their risk of stroke, bleeding, and mortality to promote informed decision-making to prescribe OACs.

Another contributing factor to the under-prescription of OACs in AF patients is history of major bleeding events during anticoagulation therapy.6,28,29. A meta-analysis of observational studies that evaluated the effect of reinitiating OACs on bleeding reoccurrence and thromboembolic events found a reduction in the risk of thromboembolism and all-cause mortality, without increasing reoccurrence of bleeding.30 Since randomized controlled trials (RCTs) that tested OACs, have excluded patients with previous intracranial haemorrhage (ICH), the timing to reinitiate OACs after ICH has not been systemically studied, and varies widely in observational studies from (72 hr to 30 weeks) reflecting lack of consensus.31 Moreover, of the available bleeding risk scores, the HAS-BLED score has been validated to predict ICH and recurrent ICH after first spontaneous ICH.32 However, it remains unclear if other risk assessment scores for stroke and bleeding hold similarly in these high-risk individuals and if they could be used to inform

decision-making for reinitiating OACs in these patients.

The available evidence presents several key aspects of AF management and the challenges associated with OACs prescribing in this population. There are still limitations in studies addressing the under-prescription of OACs in AF patients including patients with history of major bleeding events. In this proposed study we first aim to explore the incidence of NVAF in England to assess patients' characteristics and predictors of OACs prescribing in patients with NVAF in general and in those who have cancer as well. By examining the characteristics of NVAF patients who are less likely to receive a prescription for OACs, we will be able to further evaluate and compare the clinical outcomes of this sub-group of patients with a similar cohort of AF patients treated with OACs. Results could help determine in which AF patients at risk for stroke, OACs can be safely prescribed, taking in account patients' comorbidities, stroke and bleeding risk profiles. In addition to that, by looking separately at patients with history of major bleeding due to OACs, we

aim to evaluate the timing of reinitiating OACs and if stroke and bleeding risk assessment using the available risk prediction scores could be used to inform decision-making for reinitiating OACs in these patients, and their clinical outcomes.

Study type

Phase one will be a hypothesis generating study that concerns with determining the incidence of NVAF and the epidemiology of the under-prescription of anticoagulants in NVAF patients in England. Phase two and three are hypothesis testing studies, that involves the use of data obtained from phase one to make statistical decisions about the associations of prescribing OACs or restarting OACs after MB with developing the predefined clinical outcomes.

Study design

The first phase of this study will be a large population-based cohort study to identify incident cases of NVAF between January 1st, 2009 and 30th of June 2019; to determine the proportions of NVAF patients who have received a prescription for OACs or no treatment; to examine predictors of OACs prescribing. Given the described study aims and objectives, this study design will allow for a longitudinal analysis of incident NVAF cases and the subsequent timing of OACs prescribing with no comparator groups.

The second phase will be a comparative cohort study that aims to compare the clinical outcomes of NVAF based on their exposure status to anticoagulation therapy. Both patient groups will be identified from phase one and further divided into comparative groups of patients who have been prescribed OACs and patients with no anticoagulation therapy. The comparison will be undertaken after controlling for factors that could influence a patient’s exposure to OACs and the outcomes to be explored, more details provided in section M.

Finally, the third phase of this study will be a separate analysis of the treatment group from phase two who have experienced major bleeding events. This study design is suitable for this phase as it will allow for a longitudinal analysis of NVAF patients from the indexed bleeding events and onward to evaluate the clinical outcomes (i.e. stroke, bleeding reoccurrence and mortality) related to OACs resumption or not and the timing of restarting OACs after the bleeding event, it will also allow for post-haemorrhage assessment of stroke and bleeding risk using different risk scores, and the identification of predictors for not resuming anticoagulation therapy.

Feasibility counts

Earlier reports on AF prevalence in England estimated that 2.5% of the population have AF for both valvular and non-valvular AF (NVAF), with increasing prevalence in older age-groups.2 A previous study, that used CPRD GOLD to identify incident cases of NVAF, identified around 91,000 cases from 2001 to 2013. We conducted a simple feasibility count for incident cases of NVAF patients in CPRD GOLD, and we identified at least 120,000 incident cases are expected for the period (2009-2019) with an annual average of around 11,000 new cases. According to a previous study in the UK that estimated the proportion of AF patients at risk for stroke but did not receive either OACs or antiplatelets to be around 12% of total AF cases.4 Therefore, we expect to identify around 14,000 NVAF patients not treated with OACs across the duration of the study

Sample size considerations

Due to the comparative nature of the second phase of this study, a sample size calculation was undertaken to provide an estimate of the number of cases needed for the second phase of this project (the comparative cohort), and to detect a difference in the major outcomes of interest (ischemic stroke, mortality, or bleeding) between OACs exposed and not exposed NVAF patients. Power calculations were based on stroke within a year of NVAF diagnosis since it is our primary outcome of interest. An Earlier study showed a yearly stroke incidence ranging of 8% with no OACs and 2.8% in patients treated with warfarin.33 We have assumed an alpha level (two-tailed) of 0.05, a beta of 0.2 (80% power), and a ratio of 5:1 for the OACs exposed-to-unexposed ratios in the matched cohort to detect a relative risk of 2.8 for the dichotomous endpoint (i.e. stroke). This would require a minimum sample number of 970 of patients in OACs treated patients and 194 patients in OACs unexposed patients, giving a total of 1164 patients. Thus, we estimate to have many times the needed numbers in the two databases.

Planned use of linked data and benefit to patients in England and Wales

For the current study, data linkages to the Hospital Episode Statistic (HES), Office for National Statistics (ONS) death registration data and index of multiple deprivation (IMD) will be required. As the included patients for particular phases will be those eligible for data linkage, then that population will be based on English practices consented to the CPRD linkage scheme. Access to the HES and ONS Mortality data are important aims as we plan to assess the association between patients' outcomes (bleeding events, stroke, and mortality) and OACs prescribing status in NVAF patients. All outcomes will be identified over the course of follow-up through relevant Read/SNOMED codes in patients' electronic records. Stroke or bleeding events will be identified through Read/SNOMED codes in patients primary care records and through linkage to inpatient HES ICD-10 codes in case of hospitalization secondary to stroke or bleeding. ONS data will be used to ascertain specific causes of death (i.e. bleeding related, cardiovascular, or all-cause death), according to ICD-10 codes. While both practice and patient level IMD data will be used to describe OACs prescribing status in terms of level of social deprivation (IMD quintiles), and as covariates in the regression models to be tested across all three phases. By using linked database that include access to longitudinal information on diagnoses, symptoms, laboratory tests, and mortality records, we will be able to thoroughly explore and compare the different outcomes associated with prescribing OACs in NVAF patients. Such information would help to inform guidelines and optimise treatment decisions of OACs for patient with NVAF.

Definition of the study population

Patients will be identified from CPRD, both GOLD and Aurum databases will be used as the primary care data source

Selection of comparison groups/controls

For the second phase of this study, we aim to compare the clinical outcomes of patients with NVAF prescribed OACs and who were not prescribed OACs. This will be done by including incident cases of NVAF identified from phase one cohort from 2009 to 2019 and further divide them into two groups; cases are patients treated with OACs within one year of NVAF diagnosis and controls are those who did not receive OACs for ?1 year after NVAF diagnosis. Many confounders will be controlled for when evaluating the effect of treatment (OACs) on the clinical outcomes of interest (i.e. stroke, bleeding, or all-cause mortality). Potential confounders are likely to include patient age, sex, and presence of hypertension, diabetes, coronary artery disease, heart failure, kidney disease, liver disease, peripheral artery disease, fall risk, history of stroke or bleeding, malignancy, anaemia, alcohol abuse, antiplatelet use or non-steroidal anti-inflammatory drugs. At first, we aim to perform propensity score matching method using a multivariable logistic regression model that contains the confounders mentioned above to estimate patient's probability to receive OACs. Matching will operate by taking each case and finds the closest propensity score match among controls, within a predetermined caliper bandwidth. However, in case if the matching method failed to achieve balance among the two patient groups or caused substantial exclusion of individuals from the main cohort, then we will consider overlap propensity score weighting. By applying overlap propensity score weighting each patient's will be weight by the probability of that patient being assigned to the opposite treatment group.

Exposures, outcomes and covariates

1. Phase 1 – NVAF Cohort

Exposures: At least one diagnostic code of AF which will be defined specifically as a diagnosis of non-valvular AF (as listed in Appendix 1), up to 30th June 2019. Primary care patient clinical, and referral records will be used to identify exposures.

Outcomes:

The primary outcomes will be the prescription of OACs either VKA or NOACs, product codes for OACs are described in (Appendix 2). Status of anticoagulation therapy will be classified as either OAC prescribed or OAC not prescribed. OAC prescription will be defined as the presence of any OACs prescription during the study period. Treatment patterns for OACs users will be evaluated for (1) continuous exposure to OACs: no treatment change until end of follow-up period, defined starting from the date of first prescription to the intended duration of that prescription, plus the duration of any overlapping and successive prescriptions of the same drug within 30 days after the expected end of a prescription.34 For VKAs, INR measurements if available will be treated as indicator for VKA exposure and therefore treated in the same way as prescriptions; (2) OACs discontinuation: no OACs prescription after a period of therapy without resumption of OACs; (3) OACs switch: this will include four switching categories: from antiplatelet only to OACs, from VKA to NOAC, from NOACs to VKA, and between NOACs switches. OACs not prescribed will be defined as no prescription record of an OACs at any time after NVAF diagnosis, this will also include patients prescribed lone antiplatelet therapy. Proportions of patients prescribed antiplatelets such as aspirin or other antiplatelets (i.e., clopidogrel, ticagrelor, or prasugrel) within the study period will be described for both OACs exposed and not exposed patients. Products codes of antiplatelet agents can be found in (Appendix 2).

Covariates:

• Patients' demographic information (age, gender, ethnicity, body mass index (BMI), geographic location (rural vs. urban), smoking status, alcohol consumption, Charlson comorbidity index for the severity of comorbidity (Appendix 4), electronic frailty index (eFI), and the index of multiple deprivation (IMD).

• For the sub-group of patients with NVAF and history of cancer, cancer diagnosis will be assessed at the index date of NVAF diagnosis and classified by cancer type into breast, prostate, colorectal, haematological, lung , and other types, and by cancer metastasis status.

• Drugs that affect either stroke or bleeding, or commonly interact with OACs, such as antihypertensive drugs, statins, antiarrhythmics, antidiabetic drugs, anticonvulsants, non-steroidal

anti-inflammatory drugs, corticosteroids, systemic azoles, HIV protease inhibitors and ciclosporin. (Appendix 2).
• The CHA2DS2-VASc stroke risk score that consists of 9 points, one for each of: congestive heart failure (CHF), hypertension (HTN), age ?75 years (x 2 points), diabetes mellitus (DM), prior stroke or transient ischemic attack (TIA) or thromboembolism (x 2 points), vascular disease, age 65 to 74 years, and sex category. Information included in the CHA2DS2-VASc score will be identified using Read codes (Appendix 3 and 4), except for age and sex, to build the score and assess the risk of stroke for all included patients in the cohort. Further categorization of eligibility for OACs will be carried according to individual CHA2DS2-VASc scores; “not eligible for OAC” if CHA2DS2-VASc score= 0 in males, or 1 in females; and “OAC eligible” if CHA2DS2-VASc score ?1 in males, or ?2 in females.
• Bleeding risk assessment using the HAS-BLED score which consists of 9 points, one for each of: hypertension, abnormal kidney or liver function (1 point each), stroke, history of bleeding or predisposition, labile INR, elderly (> 65 years), and drugs/alcohol concomitantly (1 point each). All information included in the HAS-BLED score will be identified using Read codes (Appendix 3 and 4), except for age, to build the score and assess patients’ risk for bleeding. Labile INR will not be included since it is not recorded consistently in the CPRD and because we aim is to identify the presence of prescriptions for VKA rather than checking for patient’s adherence to VKA. Bleeding tendency or predisposition will be identified through Read codes for anaemia and gastric, duodenal, and peptic ulcers (Appendix 4). Also, since alcohol consumption in not well documented in primary care, whenever possible patients will score a point for this element if there is a Read code record of heavy or problematic drinking. The bleeding risk will be categorized according to HAS-BLED score; low risk (0-1), intermediate risk (2), and high risk (?3).
• Bleeding risk assessment using the HEMORR2HAGES score which consists of the following variables: Hepatic/renal disease (1 point each); ethanol abuse (1 point); malignancy; age > 75 y (1 point); low platelets count (1); re-bleeding risk (2 points); high blood pressure (1); anaemia (1); genetic factors (1 point); falls risk (1 point); stroke (1). All these variables will be identified using Read codes (Appendix 3 and 4), except for age, to build the score and assess patients’ risk for bleeding except for genetic factors which are not accurately captured in the CPRD.
• Combined assessment of stroke, bleeding, and mortality risk using the GARFIELD-AF model relies on the following variables: age (in years), weight, heartrate, diastolic blood pressure, ethnicity, sex, history of bleeding, heart failure, history of stroke, diabetes, smoking status, dementia, use of antiplatelet, carotid occlusive disease, chronic kidney disease (CKD) (stages III–V) and vascular disease, in which the latter is defined as history of myocardial infarction (MI) or unstable angina, aortic or peripheral artery disease. These conditions will be identified using Read codes (Appendix 3 and 4).
All risk assessment scores will be measured at each patient’s index date.

2. Phase 2 - Comparative study

Exposures: In this cohort, prescriptions for OAC will determine patient’s exposure status. NVAF patients identified from phase one and who have received a continuous prescription of either VKAs or NOACs within one year of NVAF diagnosis will be in the OACs “exposed group”. While “OACs unexposed” patients are individuals in phase one who haven’t received a prescription for anticoagulation therapy and remained OACs free for ?1 year from NVAF diagnosis.

Outcomes:
The primary outcome is the composite of ischaemic stroke and other thromboembolic events, or the occurrence of MB events defined according to the ISTH criteria (described in detail in Section D). Secondary outcomes will include CRNMB events that did not satisfy the ISTH criteria and led to hospitalization, medical or surgical treatment, or led to discontinuation of OACs. All outcomes will be identified over the course of follow-up through the identification of corresponding Read codes in patients’ records. For bleeding events sense CPRD does not state the number of transfused blood units, any need for blood transfusion along with a Read code for bleeding will be classified as a MB event. Stroke or bleeding events will be identified through Read codes in patients primary care records and through linkage to inpatient HES ICD-10 codes in case of hospitalization secondary to stroke or bleeding. ONS mortality data will be used to ascertain specific causes of death (i.e. bleeding related, cardiovascular, or all-cause death) according

ICD-10 codes. All codes relevant to these outcomes are listed in Appendix 3.

Covariates:
Covariates believed to potentially confound the relationship between anticoagulation therapy and the outcomes of interest were selected based on previously established associations. These includes age and sex (will be largely accounted for through the matched study design). Stroke risk assessment using CHA2DS2-VASc score and the GARFIELD-AF model, bleeding risk using HAS-BLED and HEMORR2HAGES scores, Charlson comorbidity index, eFI, and IMD. Concomitant use of medications will also be described in the context of drugs that affect either stroke or bleeding, or commonly interacts with OACs. Such medications would include drug from therapeutic classes of aspirin and other antiplatelets, antihypertensive, statins, antiarrhythmics, diabetic drugs, anticonvulsants, non-steroidal anti-inflammatory drugs, corticosteroids, systemic azoles, HIV protease inhibitors and ciclosporin.

3. Phase 3 – Major bleeding events study cohort

Exposures:
The exposure of interest will be the occurrence of major bleeding events in the OACs exposed group from the phase two cohort. The exposure will be identified by at least one diagnostic code of a major bleeding event defined specifically according to the ISTH criteria (described in detail in Section D). Read/SNOWMED codes will be used to identify this exposure from patient's primary care electronic records, and hospitalization secondary to bleeding are to be identified through linkage to HES using ICD-10 codes (as listed in Appendix 3).

Outcomes:
The primary outcomes include the reoccurrence of MB events or CRNMB for patients who did or did not continue OACs, the composite of stroke and other thromboembolic events, bleeding or cardiovascular related mortality, and all-cause mortality. Secondary outcome is the prescription of any OACs after the occurrence of the major bleeding events (VKAs or NOACs). We also aim to explore the timing of reinitiating OACs from the indexed bleeding event until the date of OACs prescription, and weather patients have continued on the same OAC before the index haemorrhage event, were switched to another agent after bleeding have occurred (i.e. from warfarin to any NOACs or from any NOACs to warfarin) or received lone antiplatelet therapy.

Covariates:
• Patients' demographic information (age, gender, ethnicity, body mass index (BMI), geographic location (rural vs. urban), smoking status, alcohol consumption, Charlson comorbidity index for the severity of comorbidity (Appendix 3 and 4), electronic frailty index (eFI), and the index of multiple deprivation (IMD).
• Clinical covariates such as CHA2DS2-VASc, GARFIELD-AF, HAS-BLED, and HEMORR2HAGES risk assessment scores all at the time of the index haemorrhage event.

• Anatomic location of the index MB event will be identified and classified according to the critical site of bleeding (i.e., intraspinal, intracranial, intraocular, intra-articular, pericardial or other bleeding sites).

Data/statistical analysis

1. Phase 1 - NVAF cohort: Annual and crude incidence rates of NVAF cases will be calculated and expressed as person-time at risk with 95% confidence intervals (95% CI). Incidence rates will be calculated by dividing the number of new diagnosed NVAF cases each year by the total population at risk during the same year. NVAF incidence in each calendar year of the study period (2009-2018) will be stratified by age and further standardized by age and deprivation quantiles. Status of anticoagulation therapy will be classified as either OAC prescribed or OAC not prescribed. OAC prescribed patients are those who received prescriptions for OACs during the study period. Treatment patterns for OAC users will be evaluated for OACs continuation, discontinuation or switched). OACs not prescribed will be defined as no prescription record of an OACs at any time after NVAF diagnosis, this will also include patients prescribed lone antiplatelet therapy. Proportions of patients prescribed antiplatelets will be described for both OACs prescribed and not prescribed patients. Baseline demographic, and clinical characteristics, and status of cancer diagnosis across OACs prescribed and not prescribed groups will be presented as number and percentage, mean± (SD), or median (interquartile range), as appropriate. For both groups' eligibility for OACs will be described and categorized based on stroke risk according to CHA2DS2-VASc score. Trends in OACs initiation, switching from antiplatelet only to OACs, and from VKA to NOAC will be explored for each year and further stratified by eligibility for OACs and deprivation quantiles. To identify variables independently associated with OACs prescribing multivariate logistic regression models will be used and fitted with the covariates described in section N. Similarly, in a separate analysis for patients with NVAF and previously diagnosed cancer multivariate logistic regression models will be fitted to explore variables independently associated with OACs prescribing (i.e, cancer types and metastasis status). The level of statistical significance was set at P <0.05.

2. Phase 2 – Comparative study: For comparison of demographic variables and comorbidities among OACs treated and not treated individuals, independent sample t tests will be used for numeric variables, while Chi squared ?2 tests or Fisher exact tests will be used for categorical variables. To control for imbalances in patient characteristics between cohorts, we will calculate an exposure-specific propensity score as the predicted probability of receiving OACs conditional upon individuals baseline covariate (as described in section M) using logistic regression models. Then we will consider applying propensity score matching methods to match each OACs unexposed controls to up to 5 NVAF OACs exposed cases using a calliper of 0.2 standard deviation (SD) of the propensity score logit. After matching, Cox regression will be used to compute hazard ratios (HRs) of the outcomes of interest, comparing exposure to OACs versus no OACs. Initially crude HRs will be obtained, then stratified by type of OACs (i.e., VKAs and NOACs) and adjusted for age, and other important covariates described in section N. In this phase immortal time bias would occur due to the definition of exposure, where in the time from AF diagnosis till receipt of OACs those in the "OACs prescribed group" cannot have the outcome by design, otherwise they would have been classified as non OAC users. To avoid the problem of immortal time bias, we will consider using time-varying exposures, where in a survival analysis, the time period previous to the index date of OACs initiation is reclassified as nonuser for those in the "OACs prescribed group".

3. Phase 3 – Major bleeding events cohort: At first, we will use descriptive statistics to present the overall characteristics of this cohort who have experienced major bleeding events while taking OACS. After that patients will be divided to 2 groups one for the patients who resumed anticoagulation after the index major bleeding event and the other for the group who remained OACs free and will compare the patient characteristics between the two groups using Chi squared ?2 tests, Fisher's exact tests, and analysis of variance, as appropriate. Survival (time-to-event) models will be constructed to compare the time between the indexed major bleeding to the earliest event of stroke, bleeding, or all-cause mortality considering if patients were restarted on OACs or not. In Cox models we will control for age, time to OACs resumption, CHA2DS2-VASc

score and HAS-BLED score, Charlson comorbidity index, and eFI. In a secondary analysis, we will group patients who resumed OACs according to the type of anticoagulation drug used after the indexed bleeding events and compare the outcomes using Cox models in a similar manner. We will assess the proportional hazards assumption by examining Schoenfeld residuals and confirming that it is not violated. To identify variables independently associated with resuming

OACs after the indexed bleeding event multivariate logistic regression models will be used and fitted with the covariates described in section N. All the statistical analyses will be carried out using STATA (version 16).

Plan for addressing confounding

In phase 2, we aim to conduct a comparison between NVAF patients who were exposed to OACs (cases) and patients who were not exposed to OACs (controls). We will calculate an exposure-specific propensity score as the predicted probability of receiving OACs conditional upon the effect of potential confounders (described in section M). Then we will use a propensity score matching method to achieve similarity of measured confounders across cases and control groups. When estimating HRs using Cox regression models for the outcomes of interest among patient groups, we will also adjust for age, sex, IMD, stroke risk, bleeding risk, frailty, Charlson comorbidity index, and prescription of antiplatelets.

.

In phase 3, when looking at the predictors of restarting OACs after the indexed bleeding events we will take in account many confounders that could influence this decision, this will be done by using a multinomial logistic regression to adjust for the covariates described in section N. These covariates will also be considered when conducting survival analysis to compare the time between the indexed major bleeding to the earliest event of stroke, bleeding, or death among patients who restarted OACs and those who did not. Also, when evaluating the outcomes of restarting OACs we will consider the timing of restarting anticoagulation after bleeding, and stratify patients based on different time periods of OACs reinitiating.

In both phase 2 and 3, an additional analysis will be considered to assess the robustness of results to unmeasured confounding using the E-value methodology. This estimates what the relative risk would have to be for any unmeasured confounder to overcome the observed association of OACs prescribing with the clinical outcomes of stroke, bleeding, or mortality.

Plans for addressing missing data

It is expected that there will be a substantial amount of missing data for BMI, smoking status and alcohol consumption as these variables may not be regularly updated by GP practices. We have considerable expertise within the team in data management of CPRD data and two members (EK and RP) have developed algorithms to impute and model longitudinal trends of BMI, alcohol consumption, and smoking data. We will use logistic regression to ascertain whether other variables (age, gender or comorbidities) predict whether these data are missing. If these variables do not predict the missing data, then we will assume that the data are missing at random. If the data are missing at random, we will impute the missing values using multiple imputation and conduct a sensitivity analysis to investigate the effect of the missing data on the results of the analyses.

Patient or user group involvement

The University of Manchester is home to several established PPIE groups working in areas highly relevant to this proposal including e-health research, primary care, and patient safety. With the help of PRIMER (Primary Care Research in Manchester Engagement Resource) group based at the Centre for Primary Care and Health Services Research, we plan to have at least two PPIE partners with NVAF involved in this study throughout, with links to wider patient groups. They will provide feedback on all aspects, focusing on communication of findings and implications

Plans for disseminating & communicating

Results will be disseminated through the publication of academic papers, conference posters or presentations and a PhD thesis.

Conflict of interest statement

AA: declares no competing interests.
SZ: declares no competing interests.
RP: declares no competing interests.
DMA: DMA reports research grants from AbbVie, Almirall, Celgene, Eli Lilly, Janssen, Novartis, UCB, and the Leo Foundation

Limitations of study design

Data on patient characteristics and other covariates included in this study may not be fully complete in CPRD. One of the foremost limitations of this analysis was that we will not be able to calculate a full HAS-BLED and HEMORR2HAGES scores for each patient, and therefore fully ascertain bleeding risk, (as a high risk of bleeding may have led to an anticoagulant not being prescribed). Imputation methods will be explored and applied to minimize the impact of the missing data. Sensitivity analysis will also be carried to investigate the effect of the missing data on the results of the analysis, these includes alcohol consumption and labile INR for the calculation of HAS-BLED, and genetic factors included in the HEMORR2HAGES score. Due to the retrospective nature of the study, data on adherence to OACs may not be well recorded and difficult to assess. Also, we might not be able to capture the exact timing of reinitiating OACs after MB events, since some patients might resume anticoagulation in hospital settings and such information will not be available through HES APC linkage.

References

1. Schulman S, Kearon C, Haemostasis. on behalf of the S on C of A of the S and SC of the IS on T and. Definition of major bleeding in clinical investigations of antihemostatic medicinal products in non-surgical patients. Scientific and Standardization Committee Communication. J Thromb Haemost. 2005;3:692-694. doi:10.1111/j.1538-7836.2009.03678.x
2. Atrial fibrillation prevalence estimates in England. Published 2017. Accessed February 7, 2020. https://assets.publishing.service.gov.uk/government/uploads/system/uploads/attachment_data/file/6
Pisters R, Lane DA, Nieuwlaat R, et al. A novel user-friendly score (HAS-BLED) to assess 1-year risk of major bleeding in patients with atrial fibrillation: The euro heart survey. Chest. 2010;138:1093-1100. doi:10.1378/chest.10-0134
4. Adderley NJ, Ryan R, Nirantharakumar K, Marshall T. Prevalence and treatment of atrial fibrillation in UK general practice from 2000 to 2016. Heart. 2019;105:27-33. doi:10.1136/heartjnl-2018-312977
5. Loo SY, Dell'Aniello S, Huiart L, Renoux C. Trends in the prescription of novel oral anticoagulants in UK primary care. Br J Clin Pharmacol. 2017;83:2096-2106. doi:10.1111/bcp.13299
6. Lacoin L, Lumley M, Ridha E, et al. Evolving landscape of stroke prevention in atrial fibrillation within the UK between 2012 and 2016: A cross-sectional analysis study using CPRD. BMJ Open. 2017;7(e015363):1-15. doi:10.1136/bmjopen-2016-015363
7. Kirchhof P, Benussi S, Kotecha D, et al. 2016 ESC Guidelines for the management of atrial fibrillation developed in collaboration with EACTS. Eur Heart J. 2016;37:2893-2962. doi:10.1093/eurheartj/ehw210
8. NICE. National Institute for Health and Care Excellence. Atrial fibrillation: management (CG180). Published 2014. Accessed February 1, 2020. https://www.nice.org.uk/guidance/cg180
9. Lip GYH, Banerjee A, Boriani G, et al. Antithrombotic Therapy for Atrial Fibrillation: CHEST Guideline and Expert Panel Report. Chest. 2018;154(5):1121-1201. doi:10.1016/j.chest.2018.07.040
10. DeWilde S, Carey IM, Emmas C, Richards N, Cook DG. Trends in the prevalence of diagnosed atrial fibrillation, its treatment with anticoagulation and predictors of such treatment in UK primary care. Heart. 2006;92:1064-1070. doi:10.1136/hrt.2005.069492
11. Sandhu RK, Bakal JA, Ezekowitz JA, McAlister FA. Risk stratification schemes, anticoagulation use and outcomes: The risk - Treatment paradox in patients with newly diagnosed non-valvular atrial fibrillation. Heart. 2011;97:2046-2050. doi:10.1136/heartjnl-2011-300901
12. Cowan C, Healicon R, Robson I, et al. The use of anticoagulants in the management of atrial

fibrillation among general practices in England. Heart. 2013;99:1166-1172. doi:10.1136/heartjnl-2012-303472
13. Friberg L, Hammar N, Ringh M, Pettersson H, Rosenqvist M. Stroke prophylaxis in atrial fibrillation: Who gets it and who does not? Report from the Stockholm Cohort-study on Atrial Fibrillation (SCAF-study). Eur Heart J. 2006;27:1954-1964. doi:10.1093/eurheartj/ehl146
14. Waldo AL, Becker RC, Tapson VF, Colgan KJ. Hospitalized patients with atrial fibrillation and a high risk of stroke are not being provided with adequate anticoagulation. J Am Coll Cardiol. 2005;46:1729-1736. doi:10.1016/j.jacc.2005.06.077
15. Gallagher AM, Rietbrock S, Plumb J, Van Staa TP. Initiation and persistence of warfarin or aspirin in patients with chronic atrial fibrillation in general practice: Do the appropriate patients receive stroke prophylaxis? J Thromb Haemost. 2008;6:1500-1506. doi:10.1111/j.1538-7836.2008.03059.x
16. Leizorovicz A, Boneu B, Cohen A, et al. Influence of age on the prescription of vitamin K antagonists in outpatients with permanent atrial fibrillation in France. Pharmacoepidemiol Drug Saf. 2007;16:32-38. doi:10.1002/pds.1329
17. Rash A, Downes T, Portner R, Yeo WW, Morgan N, Channer KS. A randomised controlled trial of warfarin versus aspirin for stroke prevention in octogenarians with atrial fibrillation (WASPO). Age Ageing. 2007;36:151-156. doi:10.1093/ageing/afl129
18. Van Walraven C, Hart RG, Connolly S, et al. Effect of age on stroke prevention therapy in patients with atrial fibrillation: The atrial fibrillation investigators. Stroke. 2009;40:1410-1416. doi:10.1161/STROKEAHA.108.526988
19. Chao TF, Liu CJ, Lin YJ, et al. Oral anticoagulation in very elderly patients with atrial fibrillation: A nationwide cohort study. Circulation. 2018;138(1):37-47. doi:10.1161/CIRCULATIONAHA.117.031658
20. Krittayaphong R, Winijkul A, Kunjara-Na-Ayudhya R, et al. Adherence to Anticoagulant Guideline for Atrial Fibrillation Improves Outcomes in Asian Population. Stroke. 2020;51:1772-1780. doi:10.1161/strokeaha.120.029295
21. Mazurek M, Shantsila E, Lane DA, Wolff A, Proietti M, Lip GYH. Guideline-Adherent Antithrombotic Treatment Improves Outcomes in Patients With Atrial Fibrillation: Insights From the Community-Based Darlington Atrial Fibrillation Registry. Mayo Clin Proc. 2017;92(8):1203-1213. doi:10.1016/j.mayocp.2017.05.023
22. January CT, Wann LS, Alpert JS, et al. 2014 AHA/ACC/HRS guideline for the management of patients with atrial fibrillation: A report of the American college of Cardiology/American heart association task force on practice guidelines and the heart rhythm society. J Am Coll Cardiol. 2014;130:e199-e267. doi:10.1016/j.jacc.2014.03.022
23. Navi BB, Reiner AS, Kamel H, et al. Risk of Arterial Thromboembolism in Patients With Cancer. J Am Coll Cardiol. 2017;70(8). doi:10.1016/j.jacc.2017.06.047
24. Hindricks G, Potpara T, Dagres N, et al. 2020 ESC Guidelines for the diagnosis and management of atrial fibrillation developed in collaboration with the European Association for Cardio-Thoracic Surgery (EACTS). Eur Heart J. 2021;42(5). doi:10.1093/eurheartj/ehaa612
25. Lip GYH, Laroche C, Popescu MI, et al. Improved outcomes with European Society of Cardiology guideline-adherent antithrombotic treatment in high-risk patients with atrial fibrillation: A report from the EORP-AF General Pilot Registry. Europace. 2015;17:1777-1786. doi:10.1093/europace/euv269
26. Fox KAA, Lucas JE, Pieper KS, et al. Improved risk stratification of patients with atrial fibrillation: An integrated GARFIELD-AF tool for the prediction of mortality, stroke and bleed in patients with and without anticoagulation. BMJ Open. 2017;7(e017157):1-11. doi:10.1136/bmjopen-2017-017157
27. Dalgaard F, Pieper K, Verheugt F, et al. GARFIELD-AF model for prediction of stroke and major bleeding in atrial fibrillation: A Danish nationwide validation study. BMJ Open. Published online 2019. doi:10.1136/bmjopen-2019-033283
28. Bahri O, Roca F, Lechani T, et al. Underuse of oral anticoagulation for individuals with atrial fibrillation in a nursing home setting in france: Comparisons of resident characteristics and physician attitude. J Am Geriatr Soc. 2015;63(1):71-76. doi:10.1111/jgs.13200
29. Palomäki A, Mustonen P, Hartikainen JEK, et al. Underuse of anticoagulation in stroke patients with atrial fibrillation - the FibStroke Study. Eur J Neurol. 2016;23:133-139. doi:10.1111/ene.12820
30. Zhou Y, Guo Y, Liu D, Feng H, Liu J. Restarting of anticoagulation in patients with atrial

fibrillation after major bleeding: A meta-analysis. J Clin Pharm Ther. 2020;00:1-11.
31. Tomaselli GF, Mahaffey KW, Cuker A, et al. 2017 ACC Expert Consensus Decision Pathway on Management of Bleeding in Patients on Oral Anticoagulants. J Am Coll Cardiol. 2017;70(24):3042-3067. doi:10.1016/j.jacc.2017.09.1085
32. Chan KH, Ka-Kit Leung G, Lau KK, et al. Predictive Value of the HAS-BLED Score for the Risk of Recurrent Intracranial Hemorrhage after First Spontaneous Intracranial Hemorrhage. World Neurosurg. 2014;82:e219-e223. doi:10.1016/j.wneu.2013.02.070
33. McBride R. Stroke prevention in atrial fibrillation study: Final results. Circulation. 1991;84(2):527-539. doi:10.1161/01.CIR.84.2.527

34. Gallagher AM, Van Staa TP, Murray-Thomas T, et al. Population-based cohort study of warfarin-treated patients with atrial fibrillation: Incidence of cardiovascular and bleeding outcomes. BMJ Open. 2014;4(e003839):1-10. doi:10.1136/bmjopen-2013-003839

Appendices

Grant ID
