# Supplementary material for: Incidence of nonvalvular atrial fibrillation and oral anticoagulant prescribing in England, 2009 to 2019: A cohort study
Source: PLoS Med. 2022 Jun 7;19(6):e1004003. doi: 10.1371/journal.pmed.1004003 (PMC9173622; doi:10.1371/journal.pmed.1004003)

S1 Fig: Sex-specific annual standardized incidence rates per 10,000 patients and 95% CI from practices that contributed for 11 years or less; A) CPRD GOLD, B) CPRD Aurum

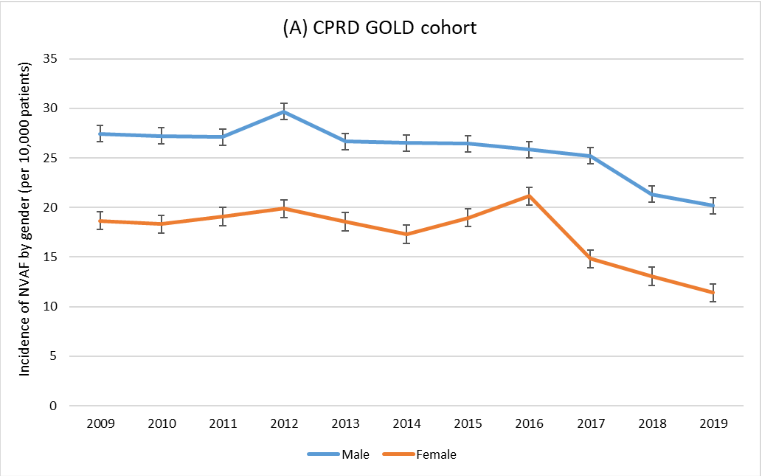

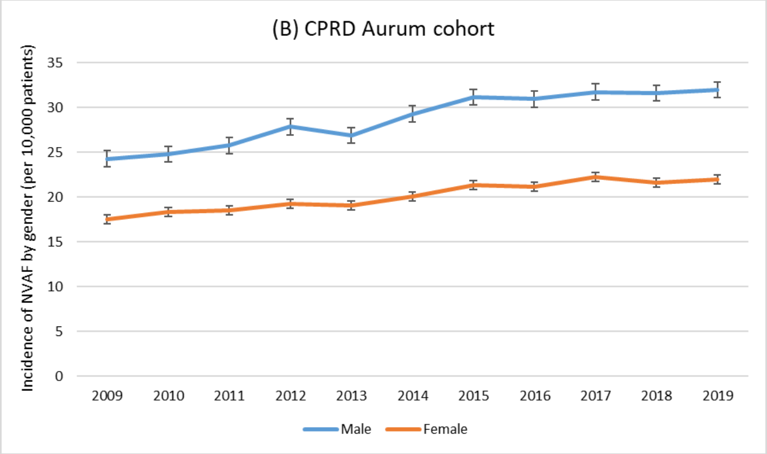

Supplement: S1 Fig — (PDF) [file pmed.1004003.s003.pdf]
