# Supplementary material for: Incidence of nonvalvular atrial fibrillation and oral anticoagulant prescribing in England, 2009 to 2019: A cohort study
Source: PLoS Med. 2022 Jun 7;19(6):e1004003. doi: 10.1371/journal.pmed.1004003 (PMC9173622; doi:10.1371/journal.pmed.1004003)

Figure S5: Predictive probabilities and 95% CI of prescribing OAC, aspirin-only, or no treatment based on practice-level IMD and ethnicity

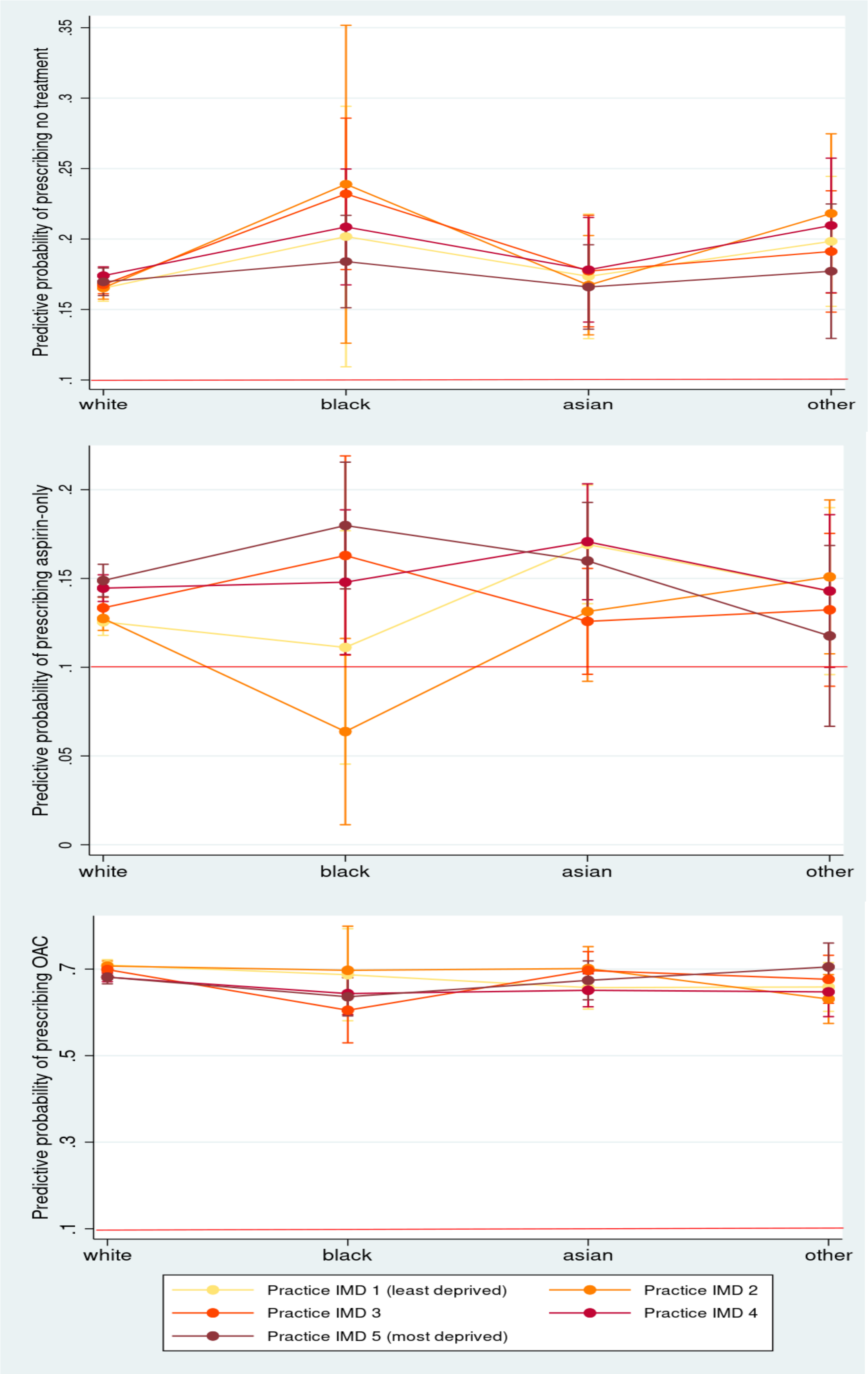

Supplement: S5 Fig — (PDF) [file pmed.1004003.s007.pdf]
