# Supplementary material for: Incidence of nonvalvular atrial fibrillation and oral anticoagulant prescribing in England, 2009 to 2019: A cohort study
Source: PLoS Med. 2022 Jun 7;19(6):e1004003. doi: 10.1371/journal.pmed.1004003 (PMC9173622; doi:10.1371/journal.pmed.1004003)
Supplement: S1 Table — (PDF) [file pmed.1004003.s009.pdf]

S1 Table: Characteristics of included general practices

	CPRD GOLD n= 238	CPRD Aurum n= 888	Overall n= 1126
**List size***			
**Mean (SD)**	9187.4 (4752.2)	9265.1 (6005.4)	9248.7 (5761.4)
**Median(IQR)**	8340.5 (5577 to 11 948)	8248.5 (4970 to 11 890)	8276 (5085 to 11 924)
**Region**			
**North east**	9 (3.8)	47 (5.3)	56 (4.9)
**North west**	27 (11.3)	161 (18.1)	188 (16.7)
**Yorkshire & the Humber**	14 (5.9)	33 (3.7)	47 (4.2)
**East Midland**	10 (4.2)	26 (2.9)	36 (3.2)
**West Midland**	17 (7.1)	191 (21.5)	208 (18.5)
**East of England**	32 (13.4)	39 (4.4)	71 (6.3)
**South West**	44 (18.5)	97 (10.9)	141 (12.5)
**South Central**	23 (9.7)	79 (9)	102 (9.1)
**London**	39 (16.4)	149 (16.8)	188 (16.7)
**South East Cost**	23 (9.7)	66 (7.4)	89 (7.9)
**Practice-level IMD**			
**1 –least deprived**	32 (13.4)	149 (16.8)	181 (16.1)
**2**	37 (15.6)	164 (18.5)	201 (17.8)
**3**	51 (21.4)	168 (18.9)	219 (19.5)
**4**	58 (24.4)	193 (21.7)	251 (22.3)
**5 – most deprived**	60 (25.2)	214 (24.1)	274 (24.3)

*Measured based on active registration of patients in each practice in the year 2009.
