# Supplementary material for: Incidence of nonvalvular atrial fibrillation and oral anticoagulant prescribing in England, 2009 to 2019: A cohort study
Source: PLoS Med. 2022 Jun 7;19(6):e1004003. doi: 10.1371/journal.pmed.1004003 (PMC9173622; doi:10.1371/journal.pmed.1004003)
Supplement: S2 Table — (PDF) [file pmed.1004003.s010.pdf]

S2 Table: Overall and sex-specific annual standardised NVAF incidence rates per 10,000 patients and 95% CI from practices that contributed data throughout the study period (for 11 years)

Year	Incidence rates (CPRD Aurum and GOLD)		
	Males	Females	Overall
**2009**	24.2 (23.6; 24.8)	17.5 (17.0; 18.0)	20.8 (20.4; 21.1)
**2010**	24.9 (24.3; 25.5)	18.3 (17.7; 18.8)	21.5 (21.1; 21.9)
**2011**	25.9 (24.9; 26.2)	18.3 (17.8; 18.8)	21.8 (21.4; 22.2)
**2012**	27.6 (26.9; 28.2)	18.9 (18.4; 19.5)	23.1 (22.7; 23.5)
**2013**	26.8 (26.2; 27.4)	19.1 (18.5; 19.6)	22.8 (22.4; 23.2)
**2014**	28.9 (28.3; 29.6)	19.9 (19.4; 20.4)	24.1 (23.7; 24.5)
**2015**	30.9 (30.2; 31.6)	21.3 (20.8; 21.9)	25.9 (25.5; 26.3)
**2016**	30.7 (30.1; 31.4)	21.3 (20.8; 21.8)	25.8 (25.4; 26.2)
**2017**	31.2 (30.5; 31.8)	21.8 (21.2; 22.3)	26.2 (25.8; 26.6)
**2018**	31.2 (30.6; 31.9)	20.9 (20.3; 21.4)	25.7 (25.3; 26.1)
**2019**	30.4 (29.8; 31.1)	21.0 (20.5; 21.6)	25.5 (25.1; 25.9)
