# Supplementary material for: Incidence of nonvalvular atrial fibrillation and oral anticoagulant prescribing in England, 2009 to 2019: A cohort study
Source: PLoS Med. 2022 Jun 7;19(6):e1004003. doi: 10.1371/journal.pmed.1004003 (PMC9173622; doi:10.1371/journal.pmed.1004003)
Supplement: S3 Table — (PDF) [file pmed.1004003.s011.pdf]

S3 Table: Sex-specific annual standardized incidence rates per 10,000 patients and 95% CI from practices that contributed for 11 years or less

Year	Incidence rate (CPRD GOLD)		Incidence rate (CPRD Aurum)	
	Males	Females	Males	Females
**2009**	27.4 (26.2; 28.6)	18.7 (17.7; 19.6)	24.3 (23.7; 24.9)	17.6 (17.1; 18.0)
**2010**	27.2 (26.0; 28.4)	18.3 (17.4; 19.3)	24.8 (24.2; 25.4)	18.3 (17.9; 18.8)
**2011**	27.1 (25.9; 28.3)	19.1 (18.1; 20.1)	25.8 (25.2; 26.3)	18.5 (18.0; 19.9)
**2012**	29.7 (28.3; 30.9)	19.9 (18.8; 20.9)	27.9 (27.3; 28.5)	19.3 (18.8; 19.8)
**2013**	26.7 (25.4; 27.9)	18.6 (17.5; 19.6)	26.9 (26.3; 27.5)	19.1 (18.6; 19.6)
**2014**	26.5 (25.1; 27.9)	17.3 (16.2; 18.4)	29.3 (28.7; 29.9)	20.1 (19.6; 20.6)
**2015**	26.4 (24.9; 27.9)	18.9 (17.7; 20.3)	31.2 (30.5; 31.8)	21.4 (20.9; 21.9)
**2016**	25.8 (24.2; 27.5)	21.1 (19.6; 22.7)	30.9 (30.3; 31.6)	21.2 (20.7; 21.7)
**2017**	25.2 (23.4; 27.1)	14.8 (13.4; 16.2)	31.8 (31.1; 32.4)	22.2 (21.7; 22.7)
**2018**	21.3 (19.6; 23.1)	13.1 (11.7; 14.4)	31.6 (31.0; 32.2)	21.6 (21.1; 22.1)
**2019**	20.2 (18.4; 21.9)	11.4 (10.1; 12.7)	31.9 (31.4; 32.6)	22.0 (21.5; 21.5)
