# Supplementary material for: Incidence of nonvalvular atrial fibrillation and oral anticoagulant prescribing in England, 2009 to 2019: A cohort study
Source: PLoS Med. 2022 Jun 7;19(6):e1004003. doi: 10.1371/journal.pmed.1004003 (PMC9173622; doi:10.1371/journal.pmed.1004003)
Supplement: S4 Table — (PDF) [file pmed.1004003.s012.pdf]

S4 Table: Sex-specific annual standardized incidence rates per 10,000 patients and 95% CI from practices that contributed throughout the study period (for 11 years)

Year	Incidence rate (CPRD GOLD)		Incidence rate (CPRD Aurum)	
	Males	Females	Males	Females
**2009**	25.3 (23.2; 27.6)	17.2 (15.4; 19.1)	24.1 (23.5; 24.8)	17.6 (17.1; 18.1)
**2010**	26.8 (24.6; 29.1)	17.9 (16.1; 19.8)	24.7 (24.1; 25.3)	18.3 (17.7; 18.8)
**2011**	25.8 (23.5; 28.0)	19.2 (17.3; 21.1)	25.5 (24.9; 26.1)	18.3 (17.7; 18.8)
**2012**	28.5 (26.1; 30.8)	18.6 (16.8; 20.5)	27.5 (26.8; 28.1)	18.9 (18.4; 19.5)
**2013**	28.6 (26.2; 30.9)	20.1 (18.2; 22.1)	26.6 (25.9; 27.2)	18.9 (18.4; 19.7)
**2014**	28.5 (26.1; 30.8)	18.3 (16.6; 20.1)	28.9 (28.3; 29.6)	20.0 (19.5; 20.6)
**2015**	33.3 (30.8; 35.8)	21.2 (19.2; 23.2)	30.7 (30.0; 31.4)	21.3 (20.8; 21.9)
**2016**	33.3 (30.8; 35.8)	25.6 (23.4; 27.7)	30.5 (29.8; 31.2)	20.9 (20.4; 21.5)
**2017**	31.9 (29.6; 34.4)	18.5 (16.7; 20.3)	31.1 (30.4; 31.7)	22.1 (21.5; 22.6)
**2018**	29.3 (26.9; 31.6)	18.7 (16.9; 20.5)	31.5 (30.8; 32.2)	21.1 (20.5; 21.6)
**2019**	29.8 (27.5; 32.2)	17.3 (15.6; 19.1)	31.4 (30.7; 32.0)	21.9 (21.4; 22.5)
