# Supplementary material for: Incidence of nonvalvular atrial fibrillation and oral anticoagulant prescribing in England, 2009 to 2019: A cohort study
Source: PLoS Med. 2022 Jun 7;19(6):e1004003. doi: 10.1371/journal.pmed.1004003 (PMC9173622; doi:10.1371/journal.pmed.1004003)
Supplement: S5 Table — (PDF) [file pmed.1004003.s013.pdf]

S5 Table: Proportions of patients prescribed OACs (VKA or NOAC), Aspirin only, or no treatment for patients eligible for OAC as illustrated in figure 4 in the manuscript.

Year	Eligible for OAC & received OAC	Eligible for OAC & did not receive OAC	Eligible for OAC & received aspirin-only
	% (95% CI)	% (95% CI)	% (95% CI)
**2009**	59.8% (59.0%; 60.6%)	10.0% (9.5%; 10.5%)	30.2% (29.5%; 31.0%)
**2010**	62.9% (62.3%; 63.5%)	8.5% (8.1%; 8.8%)	28.7% (28.1%; 29.2%)
**2011**	66.4% (66.0%; 66.9%)	7.5% (7.2%; 7.7%)	26.1% (25.7%; 26.5%)
**2012**	70.0% (69.6%; 70.4%)	7.2% (7.0%; 7.5%)	22.8% (22.4%; 23.2%)
**2013**	74.0% (73.7%; 74.4%)	7.1% (6.9%; 7.3%)	18.9% (18.6%; 19.2%)
**2014**	78.2% (77.8%; 78.5%)	7.1% (6.9%; 7.3%)	14.7% (14.5%; 15.0%)
**2015**	81.5% (81.3%; 81.8%)	7.2% (7.0%; 7.3%)	11.3% (11.1%; 11.6%)
**2016**	83.9% (83.7%; 84.2%)	7.3% (7.1%; 7.5%)	8.8% (8.6%; 9.0%)
**2017**	85.2% (85.0%; 85.4%)	7.8% (7.6%; 7.9%)	7.1% (6.9%; 7.2%)
**2018**	85.0% (84.8%; 85.2%)	8.8% (8.6%; 9.0%)	6.2% (6.0%; 6.3%)
**2019**	83.2% (83.0%; 83.4%)	11.2% (11.0%; 11.4%)	5.6% (5.5%; 5.8%)
