# Supplementary material for: Incidence of nonvalvular atrial fibrillation and oral anticoagulant prescribing in England, 2009 to 2019: A cohort study
Source: PLoS Med. 2022 Jun 7;19(6):e1004003. doi: 10.1371/journal.pmed.1004003 (PMC9173622; doi:10.1371/journal.pmed.1004003)
Supplement: S6 Table — (PDF) [file pmed.1004003.s014.pdf]

S6 Table: Proportion of patients prescribed first treatment after NVAF diagnosis stratified by the type of drug initiated, from 2009 to 2019 as illustrated in figure 5 in the manuscript.

Year	VKA		NOAC		Aspirin	
	n	% (95% CI)	n	% (95% CI)	n	% (95% CI)
**2009**	4,509	40.5% (40%; 42%)	0	0	6,632	59.5% (58%; 60%)
**2010**	5,073	40.5% (40%; 42%)	1	0	7,464	59.5% (58%; 60%)
**2011**	5,673	43.6% (43%; 45%)	19	0.2% (0.1%; 0.2%)	7,311	56.2% (55%; 57%)
**2012**	6,572	47.3% (47%; 48%)	409	2.9% (2.7%; 3.2%)	6,934	49.8% (49%; 50%)
**2013**	6,670	49.7% (49%; 51%)	1,483	11% (10%; 11%)	5,275	39.3% (38%; 40%)
**2014**	6,242	47.0% (46%; 48%)	3,394	25.6% (25%; 26%)	3,638	27.4% (27%; 28%)
**2015**	4,795	33.4% (33%; 34%)	6,909	48.2% (48%; 49%)	2,637	18.4% (18%; 19%)
**2016**	2,650	19.1% (18%; 20%)	9,320	67.3% (67%; 68%)	1,884	13.6% (13%; 14%)
**2017**	1,393	10% (9%; 10%)	10,908	78.3% (78%; 79%)	1,632	11.7% (11%; 12%)
**2018**	775	5.8% (5%; 6%)	11,132	83.2 (83%; 84%)	1,467	11% (10%; 11%)
**2019**	542	4.6% (4%; 5%)	10,256	86.1% (86%; 87%)	1,112	9.3% (9%; 10%)
