# Supplementary material for: Incidence of nonvalvular atrial fibrillation and oral anticoagulant prescribing in England, 2009 to 2019: A cohort study
Source: PLoS Med. 2022 Jun 7;19(6):e1004003. doi: 10.1371/journal.pmed.1004003 (PMC9173622; doi:10.1371/journal.pmed.1004003)
Supplement: S7 Table — (PDF) [file pmed.1004003.s015.pdf]

S7 Table: Proportions of patients prescribed OACs (VKA or NOAC), Aspirin only, or no treatment among patients not eligible for OAC

Year	Not eligible for OAC & received OAC	Not eligible for OAC & did not receive OAC	Not eligible for OAC & received aspirin-only
	**% (95% CI)**	**% (95% CI)**	**% (95% CI)**
**2009**	58.3% (56%; 61%)	22.7% (21%; 25%)	18.9% (17%; 21%)
**2010**	58.1% (56%; 60%)	21.9% (20%; 23%)	20.0% (19%; 21%)
**2011**	58.6% (57%; 60%)	22.5% (21%; 24%)	19.0% (18%; 20%)
**2012**	58.3% (57%; 60%)	23.5% (22%; 25%)	18.3% (17%; 19%)
**2013**	58.3% (57%; 59%)	25.2% (24%; 26%)	16.5% (16%; 17%)
**2014**	59.0% (58%; 60%)	26.1% (25%; 27%)	14.9% (14%; 16%)
**2015**	59.2% (58%; 60%)	28.0% (27%; 29%)	12.8% (12%; 14%)
**2016**	58.9% (58%; 60%)	30.0% (29%; 31%)	11.1% (11%; 12%)
**2017**	58.1% (57%; 59%)	32.0% (31%; 33%)	9.9% (9%; 10%)
**2018**	57.3% (56%; 58%)	33.8% (33%; 35%)	8.9% (8%; 9%)
**2019**	54.9% (54%; 56%)	36.9% (36%; 38%)	8.2% (8%; 9%)
