# Supplementary material for: Incidence of nonvalvular atrial fibrillation and oral anticoagulant prescribing in England, 2009 to 2019: A cohort study
Source: PLoS Med. 2022 Jun 7;19(6):e1004003. doi: 10.1371/journal.pmed.1004003 (PMC9173622; doi:10.1371/journal.pmed.1004003)
Supplement: S8 Table — (PDF) [file pmed.1004003.s016.pdf]

S8 Table: Results of multivariable analysis with interaction terms evaluating factors associated prescribing of OAC or aspirin-only vs no treatment (reference group) in patients recommended to take OAC

	OAC		Aspirin only	
	Adjusted RRR* 95%CI	P value	Adjusted RRR* 95%CI	P value
**Region**				
**London**	Ref			
**North east**	0.99 (0.74; 1.34)	0.970	1.17 (0.83; 1.63)	0.376
**North west**	0.87 (0.68; 1.09)	0.228	0.96 (0.73; 1.27)	0.784
**Yorkshire and the Humber**	0.83 (0.65; 1.05)	0.122	1.04 (0.74; 1.45)	0.825
**East midlands**	1.16 (0.89; 1.50)	0.266	1.18 (0.87; 1.60)	0.292
**West midlands**	1.14 (0.93; 1.40)	0.216	1.14 (0.89; 1.47)	0.307
**East of England**	1.09 (0.88; 1.34)	0.445	1.43 (1.08; 1.88)	0.011
**South west**	1.25 (1.04; 1.51)	0.020	1.29 (1.01; 1.65)	0.042
**South central**	1.09 (0.91; 1.31)	0.331	1.17 (0.92; 1.49)	0.192
**South east coast**	0.99 (0.80; 1.23)	0.934	0.93 (0.71; 1.22)	0.605
**Patient level IMD**				
**1 (least deprived)**	Ref			
**2**	0.89 (0.75; 1.05)	0.162	1.24 (0.97; 1.59)	0.086
**3**	0.87 (0.73; 1.04)	0.116	1.43 (1.13; 1.81)	0.002
**4**	0.78 (0.64; 0.94)	0.011	1.23 (0.97; 1.57)	0.088
**5 (most deprived)**	0.67 (0.56; 0.81)	<0.001	1.37 (1.06; 1.76)	0.017
**Patient level IMD x region**				
**IMD 1 and London**	Ref			
**IMD 2 in North east**	1.40 (1.01; 1.94)	0.046	0.95 (0.62; 1.45)	0.808
**IMD 2 in North west**	1.03 (0.82; 1.30)	0.809	0.85 (0.63; 1.16)	0.308
**IMD 2 in Yorkshire and the Humber**	1.18 (0.87; 1.60)	0.283	0.96 (0.67; 1.37)	0.818
**IMD 2 in East midlands**	1.11 (0.84; 1.47)	0.452	1.05 (0.74; 1.50)	0.777
**IMD 2 in West midlands**	1.07 (0.86; 1.33)	0.531	0.85 (0.63; 1.14)	0.266
**IMD 2 in East of England**	0.87 (0.67; 1.13)	0.302	0.71 (0.50; 1.01)	0.058
**IMD 2 in South west**	1.07 (0.86; 1.34)	0.525	0.79 (0.59; 1.07)	0.135
**IMD 2 in South central**	0.98 (0.81; 1.20)	0.870	0.84 (0.63; 1.11)	0.226
**IMD 2 in South east coast**	1.21 (0.96; 1.53)	0.112	0.97 (0.71; 1.33)	0.861
**IMD 3 in North east**	1.34 (0.96; 1.86)	0.086	0.91 (0.65; 1.30)	0.617
**IMD 3 in North west**	1.10 (0.86; 1.42)	0.436	0.82 (0.61; 1.11)	0.197
**IMD 3 in Yorkshire and the Humber**	1.13 (0.86; 1.49)	0.373	0.78 (0.54; 1.14)	0.196
**IMD 3 in East midlands**	1.01 (0.74; 1.36)	0.963	0.90 (0.60; 1.36)	0.624
**IMD 3 in West midlands**	0.95 (0.76; 1.20)	0.693	0.67 (0.51; 0.88)	0.004
**IMD 3 in East of England**	0.96 (0.74; 1.26)	0.777	0.73 (0.52; 1.03)	0.077

**IMD 3 in South west**	1.02 (0.82; 1.28)	0.834	0.72 (0.55; 0.95)	0.020
**IMD 3 in South central**	0.97 (0.78; 1.21)	0.797	0.73 (0.54; 0.99)	0.046
**IMD 3 in South east coast**	1.20 (0.91; 1.58)	0.187	0.89 (0.64; 1.23)	0.476
**IMD 4 in North east**	1.33 (0.91; 1.93)	0.143	1.00 (0.70; 1.42)	0.981
**IMD 4 in North west**	1.14 (0.85; 1.51)	0.389	0.92 (0.67; 1.25)	0.585
**IMD 4 in Yorkshire and the Humber**	1.36 (0.91; 2.02)	0.129	0.75 (0.50; 1.13)	0.17
**IMD 4 in East midlands**	0.97 (0.67; 1.41)	0.890	1.24 (0.78; 1.94)	0.361
**IMD 4 in West midlands**	1.09 (0.85; 1.41)	0.487	0.80 (0.59; 1.08)	0.144
**IMD 4 in East of England**	0.91 (0.67; 1.25)	0.565	0.80 (0.56; 1.15)	0.231
**IMD 4 in South west**	1.09 (0.86; 1.39)	0.466	0.80 (0.59; 1.07)	0.134
**IMD 4 in South central**	0.98 (0.76; 1.25)	0.845	0.88 (0.63; 1.23)	0.462
**IMD 4 in South east coast**	1.48 (1.08; 2.03)	0.015	1.15 (0.80; 1.64)	0.452
**IMD 5 in North east**	1.45 (1.04; 2.02)	0.028	0.98 (0.66; 1.46)	0.925
**IMD 5 in North west**	1.22 (0.92; 1.62)	0.173	0.83 (0.60; 1.15)	0.267
**IMD 5 in Yorkshire and the Humber**	1.49 (1.07; 2.06)	0.018	0.92 (0.62; 1.37)	0.685
**IMD 5 in East midlands**	1.12 (0.80; 1.56)	0.502	1.06 (0.65; 1.71)	0.827
**IMD 5 in West midlands**	1.11 (0.87; 1.42)	0.397	0.68 (0.50; 0.91)	0.01
**IMD 5 in East of England**	0.84 (0.63; 1.13)	0.260	0.80 (0.51; 1.25)	0.329
**IMD 5 in South west**	1.20 (0.94; 1.54)	0.147	0.73 (0.53; 1.01)	0.059
**IMD 5 in South central**	1.05 (0.81; 1.37)	0.697	1.01 (0.75; 1.36)	0.965
**IMD 5 in South east coast**	1.48 (1.07; 2.04)	0.017	0.70 (0.42; 1.16)	0.167
**Ethnicity**				
**White**	Ref			
**Black**	0.70 (0.34; 1.46)	0.344	1.10 (0.42; 2.90)	0.845
**Asian**	0.73 (0.54; 0.99)	0.044	0.87 (0.55; 1.38)	0.549
**Other**	0.84 (0.62; 1.14)	0.266	1.02 (0.64; 1.63)	0.922
**Patient level IMD x ethnicity**				
**IMD 2 and black**	0.96 (0.39; 2.36)	0.928	0.96 (0.39; 2.36)	0.928
**IMD 2 and Asian**	1.22 (0.82; 1.80)	0.332	1.22 (0.82; 1.80)	0.332
**IMD 2 and other**	0.79 (0.48; 1.30)	0.355	0.79 (0.48; 1.30)	0.355
**IMD 3 and black**	1.19 (0.51; 2.74)	0.691	1.19 (0.51; 2.74)	0.691
**IMD 3 and Asian**	1.30 (0.87; 1.96)	0.204	1.30 (0.87; 1.96)	0.204
**IMD 3 and other**	0.71 (0.45; 1.12)	0.144	0.71 (0.45; 1.12)	0.144
**IMD 4 and black**	1.21 (0.55; 2.68)	0.638	1.21 (0.55; 2.68)	0.638
**IMD 4 and Asian**	1.57 (1.03; 2.38)	0.036	1.57 (1.03; 2.38)	0.036
**IMD 4 and other**	1.12 (0.70; 1.78)	0.644	1.12 (0.70; 1.78)	0.644
**IMD 5 and black**	1.14 (0.53; 2.47)	0.734	1.14 (0.53; 2.47)	0.734
**IMD 5 and Asian**	1.38 (0.94; 2.05)	0.102	1.38 (0.94; 2.05)	0.102
**IMD 5 and other**	1.32 (0.81; 2.13)	0.263	1.32 (0.81; 2.13)	0.263
**Sex**				
**Male**	Ref			
**Female**	1.00 (0.97; 1.03)	0.967	1.14 (1.09; 1.19)	<0.001

**Baseline age and BMI**				
**18-40**	Ref			
**41-54**	2.65 (1.56; 4.49)	<0.001	4.40 (1.47; 13.18)	0.008
**55-64**	3.71 (2.22; 6.19)	<0.001	4.76 (1.60; 14.13)	0.005
**65-74**	2.25 (1.35; 3.77)	0.002	1.44 (0.49; 4.26)	0.506
**75-84**	2.11 (1.26; 3.52)	0.004	1.77 (0.60; 5.23)	0.298
**≥85**	0.97 (0.58; 1.62)	0.909	2.14 (0.73; 6.29)	0.167
**Disease state and disability**				
**Heart failure**	0.97 (0.93; 1.01)	0.191	0.96 (0.91; 1.02)	0.192
**Cerebrovascular disease/TIA**	0.49 (0.46; 0.51)	<0.001	0.22 (0.21; 0.24)	<0.001
**Hypertension**	0.72 (0.69; 0.76)	<0.001	0.31 (0.29; 0.33)	<0.001
**Diabetes**	1.02 (0.98; 1.06)	0.311	0.92 (0.87; 0.96)	<0.001
**Rhuematological disease**	0.93 (0.89; 0.99)	0.012	0.89 (0.83; 0.96)	0.002
**Peptic ulcer**	0.80 (0.75; 0.85)	<0.001	0.83 (0.77; 0.90)	<0.001
**Aneamia**	0.72 (0.69; 0.75)	<0.001	0.83 (0.79; 0.87)	<0.001
**Dementia**	0.44 (0.39; 0.49)	<0.001	1.29 (1.10; 1.50)	0.001
**Malignancy**	0.75 (0.72; 0.77)	<0.001	0.79 (0.76; 0.83)	<0.001
**History of bleeding**	0.53 (0.50; 0.56)	<0.001	0.31 (0.29; 0.33)	<0.001
**Chronic kidney disease**	0.56 (0.53; 0.59)	<0.001	0.34 (0.32; 0.37)	<0.001
**Peripheral vascular disease**	0.92 (0.86; 0.98)	0.007	1.05 (0.98; 1.13)	0.184
**Ischaemic heart disease**	1.21 (1.16; 1.26)	<0.001	1.21 (1.15; 1.27)	<0.001
**Myocardial infarction**	1.03 (0.97; 1.09)	0.354	1.31 (1.22; 1.41)	<0.000
**Liver disease**	0.29 (0.25; 0.33)	<0.001	0.19 (0.16; 0.23)	<0.001
**Osteoporosis**	0.90 (0.87; 0.94)	<0.001	0.89 (0.84; 0.94)	<0.001
**Arthritis**	1.15 (1.11; 1.18)	<0.001	0.95 (0.91; 0.99)	0.02
**History of falls**	0.75 (0.71; 0.78)	<0.001	0.90 (0.85; 0.95)	<0.001
**Mobility problems**	0.75 (0.71; 0.79)	<0.001	0.95 (0.90; 1.01)	0.128
**Cognitive impairment**	0.75 (0.68; 0.83)	<0.001	0.69 (0.59; 0.80)	<0.001
**Visual impairment**	0.96 (0.92; 0.99)	0.014	0.99 (0.95; 1.04)	0.682
**HASBLED score**	1.72 (1.66; 1.78)	<0.001	3.04 (2.90; 3.19)	<0.001

* Adjusted for age, sex, comorbidities, socioeconomic status, HASBLED score, and takes in account clustering by general practice.
