# Supplementary material for: Incidence of nonvalvular atrial fibrillation and oral anticoagulant prescribing in England, 2009 to 2019: A cohort study
Source: PLoS Med. 2022 Jun 7;19(6):e1004003. doi: 10.1371/journal.pmed.1004003 (PMC9173622; doi:10.1371/journal.pmed.1004003)
Supplement: S9 Table — (PDF) [file pmed.1004003.s017.pdf]

S9 Table: Marginal analysis for the predictive probability of prescribing OAC, aspirin-only, or no treatment based on ethnicity and patient-level IMD

Treatment	Interaction (Patient IMD and Ethnicity)	Margins	95% CI	
No treatment	IMD 1 and White	16%	15%	17%
	IMD 1 and Black	20%	9%	30%
	IMD 1 and Asian	20%	16%	24%
	IMD 1 and other	18%	14%	22%
	IMD 2 and White	17%	16%	17%
	IMD 2 and Black	22%	13%	31%
	IMD 2 and Asian	17%	14%	21%
	IMD 2 and other	22%	16%	27%
	IMD 3 and White	17%	16%	17%
	IMD 3 and Black	19%	14%	24%
	IMD 3 and Asian	18%	14%	21%
	IMD 3 and other	25%	20%	30%
	IMD 4 and White	17%	17%	18%
	IMD 4 and Black	20%	15%	24%
	IMD 4 and Asian	15%	12%	19%
	IMD 4 and other	19%	14%	24%
	IMD 5 and White	18%	18%	19%
	IMD 5 and Black	21%	18%	24%
	IMD 5 and Asian	18%	15%	21%
	IMD 5 and other	17%	12%	21%
Aspirin-only	IMD 1 and White	12%	11%	12%
	IMD 1 and Black	16%	5%	27%
	IMD 1 and Asian	13%	9%	17%
	IMD 1 and other	14%	9%	18%
	IMD 2 and White	13%	13%	14%
	IMD 2 and Black	14%	6%	23%
	IMD 2 and Asian	17%	13%	21%
	IMD 2 and other	15%	10%	19%
	IMD 3 and White	14%	13%	14%
	IMD 3 and Black	16%	11%	21%
	IMD 3 and Asian	13%	10%	16%
	IMD 3 and other	12%	8%	16%
	IMD 4 and White	14%	14%	15%
	IMD 4 and Black	14%	11%	18%
	IMD 4 and Asian	17%	14%	20%
	IMD 4 and other	12%	8%	16%
	IMD 5 and White	15%	15%	16%

	IMD 5 and Black	16%	13%	20%
	IMD 5 and Asian	16%	13%	19%
	IMD 5 and other	17%	12%	23%
OAC prescribing	IMD 1 and White	72%	71%	73%
	IMD 1 and Black	64%	51%	78%
	IMD 1 and Asian	67%	62%	72%
	IMD 1 and other	68%	63%	74%
	IMD 2 and White	70%	70%	71%
	IMD 2 and Black	64%	55%	73%
	IMD 2 and Asian	66%	61%	71%
	IMD 2 and other	64%	57%	70%
	IMD 3 and White	69%	68%	70%
	IMD 3 and Black	65%	59%	71%
	IMD 3 and Asian	69%	65%	73%
	IMD 3 and other	63%	57%	69%
	IMD 4 and White	68%	68%	69%
	IMD 4 and Black	66%	62%	71%
	IMD 4 and Asian	68%	63%	72%
	IMD 4 and other	69%	63%	75%
	IMD 5 and White	66%	65%	67%
	IMD 5 and Black	62%	58%	67%
	IMD 5 and Asian	66%	62%	70%
	IMD 5 and other	66%	60%	72%
