# Supplementary material for: Incidence of nonvalvular atrial fibrillation and oral anticoagulant prescribing in England, 2009 to 2019: A cohort study
Source: PLoS Med. 2022 Jun 7;19(6):e1004003. doi: 10.1371/journal.pmed.1004003 (PMC9173622; doi:10.1371/journal.pmed.1004003)
Supplement: S10 Table — (PDF) [file pmed.1004003.s018.pdf]

S10 Table: Marginal analysis for the predictive probability of prescribing OAC, aspirin-only, or no treatment based on practice region and patient-level IMD

Treatment	Interaction	Margins	95% CI	
No treatment	London and IMD 1	17%	15%	19%
	London and IMD 2	18%	16%	19%
	London and IMD 3	18%	16%	19%
	London and IMD 4	19%	17%	21%
	London and IMD 5	20%	19%	22%
	North east and IMD 1	17%	14%	19%
	North east and IMD 2	14%	12%	16%
	North east and IMD 3	15%	13%	17%
	North east and IMD 4	16%	14%	18%
	North east and IMD 5	16%	14%	18%
	North west and IMD 1	19%	16%	21%
	North west and IMD2	20%	18%	21%
	North west and IMD 3	19%	17%	20%
	North west and IMD 4	20%	18%	22%
	North west and IMD 5	21%	18%	23%
	Yorkshire and the Humber and IMD 1	19%	17%	21%
	Yorkshire and the Humber and IMD 2	18%	15%	21%
	Yorkshire and the Humber and IMD 3	19%	16%	21%
	Yorkshire and the Humber and IMD 4	18%	15%	22%
	Yorkshire and the Humber and IMD 5	18%	16%	21%
	East midlands and IMD 1	15%	13%	17%
	East midlands and IMD 2	15%	13%	16%
	East midlands and IMD 3	16%	13%	18%
	East midlands and IMD 4	17%	14%	20%
	East midlands and IMD 5	17%	14%	20%
	West midlands and IMD 1	15%	14%	17%
	West midlands and IMD 2	16%	15%	17%
	West midlands and IMD 3	17%	16%	18%
	West midlands and IMD 4	17%	16%	18%
	West midlands and IMD 5	18%	17%	20%
	East of England and IMD 1	15%	14%	17%
	East of England and IMD 2	18%	15%	21%
	East of England and IMD 3	17%	15%	19%
	East of England and IMD 4	19%	16%	22%
	East of England and IMD 5	21%	18%	24%
	South west and IMD 1	14%	13%	15%
	South west and IMD 2	15%	13%	16%

	South west and IMD 3	15%	14%	16%
	South west and IMD 4	16%	14%	17%
	South west and IMD 5	16%	14%	18%
	South central and IMD 1	16%	15%	17%
	South central and IMD 2	17%	16%	18%
	South central and IMD 3	17%	16%	19%
	South central and IMD 4	18%	16%	20%
	South central and IMD 5	18%	16%	20%
	South east coast and IMD 1	17%	15%	19%
	South east coast and IMD 2	16%	15%	17%
	South east coast and IMD 3	16%	14%	18%
	South east coast and IMD 4	15%	13%	17%
	South east coast and IMD 5	17%	14%	21%
Aspirin-only	Londond and IMD 1	11%	10%	13%
	Londond and IMD 2	15%	13%	16%
	Londond and IMD 3	16%	15%	18%
	Londond and IMD 4	16%	14%	17%
	Londond and IMD 5	18%	16%	20%
	North east and IMD 1	13%	9%	16%
	North east and IMD 2	13%	10%	15%
	North east and IMD 3	14%	12%	17%
	North east and IMD 4	15%	13%	17%
	North east and IMD 5	16%	15%	18%
	North west and IMD 1	12%	11%	13%
	North west and IMD2	13%	12%	14%
	North west and IMD 3	14%	13%	15%
	North west and IMD 4	14%	13%	16%
	North west and IMD 5	15%	14%	16%
	Yorkshire and the Humber and IMD 1	13%	10%	16%
	Yorkshire and the Humber and IMD 2	15%	12%	18%
	Yorkshire and the Humber and IMD 3	14%	12%	17%
	Yorkshire and the Humber and IMD 4	12%	10%	14%
	Yorkshire and the Humber and IMD 5	16%	13%	18%
	East midlands and IMD 1	12%	9%	14%
	East midlands and IMD 2	15%	12%	18%
	East midlands and IMD 3	16%	13%	19%
	East midlands and IMD 4	19%	15%	24%
	East midlands and IMD 5	19%	14%	23%
	West midlands and IMD 1	11%	11%	12%
	West midlands and IMD 2	12%	11%	13%
	West midlands and IMD 3	12%	12%	13%
	West midlands and IMD 4	13%	12%	14%
	West midlands and IMD 5	13%	12%	14%

	East of England and IMD 1	14%	13%	16%
	East of England and IMD 2	15%	13%	17%
	East of England and IMD 3	16%	14%	19%
	East of England and IMD 4	17%	15%	20%
	East of England and IMD 5	21%	16%	26%
	South west and IMD 1	12%	11%	13%
	South west and IMD 2	12%	11%	13%
	South west and IMD 3	13%	12%	14%
	South west and IMD 4	13%	12%	15%
	South west and IMD 5	14%	12%	15%
	South central and IMD 1	12%	11%	13%
	South central and IMD 2	14%	12%	15%
	South central and IMD 3	14%	12%	16%
	South central and IMD 4	15%	13%	17%
	South central and IMD 5	19%	17%	22%
	South east coast and IMD 1	11%	9%	12%
	South east coast and IMD 2	12%	11%	13%
	South east coast and IMD 3	13%	11%	14%
	South east coast and IMD 4	13%	10%	15%
	South east coast and IMD 5	10%	8%	13%
OAC prescribing	Londond and IMD 1	72%	70%	74%
	Londond and IMD 2	68%	66%	70%
	Londond and IMD 3	66%	64%	68%
	Londond and IMD 4	65%	63%	68%
	Londond and IMD 5	61%	59%	64%
	North east and IMD 1	71%	65%	76%
	North east and IMD 2	73%	70%	76%
	North east and IMD 3	71%	68%	74%
	North east and IMD 4	69%	67%	72%
	North east and IMD 5	68%	65%	70%
	North west and IMD 1	69%	67%	72%
	North west and IMD2	67%	65%	69%
	North west and IMD 3	67%	65%	69%
	North west and IMD 4	66%	63%	69%
	North west and IMD 5	65%	62%	67%
	Yorkshire and the Humber and IMD 1	68%	64%	72%
	Yorkshire and the Humber and IMD 2	67%	63%	71%
	Yorkshire and the Humber and IMD 3	67%	64%	69%
	Yorkshire and the Humber and IMD 4	70%	66%	74%
	Yorkshire and the Humber and IMD 5	66%	62%	70%
	East midlands and IMD 1	73%	70%	77%
	East midlands and IMD 2	71%	67%	74%
	East midlands and IMD 3	69%	65%	72%

	East midlands and IMD 4	64%	59%	69%
	East midlands and IMD 5	65%	60%	69%
	West midlands and IMD 1	73%	71%	75%
	West midlands and IMD 2	72%	71%	73%
	West midlands and IMD 3	70%	69%	72%
	West midlands and IMD 4	70%	69%	72%
	West midlands and IMD 5	69%	67%	71%
	East of England and IMD 1	70%	68%	73%
	East of England and IMD 2	66%	64%	69%
	East of England and IMD 3	67%	64%	69%
	East of England and IMD 4	64%	60%	68%
	East of England and IMD 5	58%	53%	63%
	South west and IMD 1	74%	72%	76%
	South west and IMD 2	73%	71%	75%
	South west and IMD 3	72%	70%	73%
	South west and IMD 4	71%	69%	73%
	South west and IMD 5	70%	68%	72%
	South central and IMD 1	72%	71%	74%
	South central and IMD 2	69%	68%	71%
	South central and IMD 3	69%	66%	71%
	South central and IMD 4	66%	64%	69%
	South central and IMD 5	63%	59%	66%
	South east coast and IMD 1	72%	70%	75%
	South east coast and IMD 2	72%	70%	74%
	South east coast and IMD 3	71%	68%	74%
	South east coast and IMD 4	72%	69%	76%
	South east coast and IMD 5	73%	69%	76%
