# Supplementary material for: Incidence of nonvalvular atrial fibrillation and oral anticoagulant prescribing in England, 2009 to 2019: A cohort study
Source: PLoS Med. 2022 Jun 7;19(6):e1004003. doi: 10.1371/journal.pmed.1004003 (PMC9173622; doi:10.1371/journal.pmed.1004003)
Supplement: S11 Table — (PDF) [file pmed.1004003.s019.pdf]

S11 Table: Results of multivariable analysis with interaction terms evaluating factors associated prescribing of OAC or aspirin-only vs no treatment (reference group) in patients recommended to take OAC

	OAC		Aspirin only	
	Adjusted RRR* 95%CI	P value	Adjusted RRR* 95%CI	P value
**Region**				
**London**	Ref			
**North east**	0.85 (0.52; 1.37)	0.497	0.78 (0.50; 1.23)	0.292
**North west**	0.87 (0.58; 1.31)	0.507	0.71 (0.45; 1.12)	0.137
**Yorkshire and the Humber**	0.82 (0.58; 1.14)	0.236	0.72 (0.42; 1.25)	0.248
**East midlands**	1.12 (0.85; 1.47)	0.433	0.82 (0.54; 1.25)	0.362
**West midlands**	1.12 (0.87; 1.46)	0.381	0.85 (0.57; 1.28)	0.446
**East of England**	1.09 (0.82; 1.44)	0.560	0.93 (0.62; 1.41)	0.745
**South west**	1.34 (1.03; 1.73)	0.029	0.99 (0.66; 1.46)	0.945
**South central**	1.16 (0.89; 1.50)	0.267	0.83 (0.56; 1.24)	0.370
**South east coast**	1.00 (0.75; 1.33)	0.996	0.71 (0.47; 1.08)	0.109
**Practice level IMD**				
**1 (least deprived)**	Ref			
**2**	0.95 (0.72; 1.25)	0.709	0.81 (0.55; 1.21)	0.305
**3**	0.91 (0.68; 1.23)	0.553	0.93 (0.60; 1.44)	0.747
**4**	0.82 (0.61; 1.09)	0.172	0.96 (0.65; 1.42)	0.848
**5 (most deprived)**	0.77 (0.58; 1.01)	0.059	0.96 (0.65; 1.43)	0.846
**Practice level IMD x region**				
**IMD 1 and London**	Ref			
**IMD 2 in North east**	1.59 (0.90; 2.18)	0.110	1.38 (0.80; 2.39)	0.251
**IMD 2 in North west**	1.08 (0.69; 2.18)	0.726	1.42 (0.86; 2.34)	0.168
**IMD 2 in Yorkshire and the Humber**	1.30 (0.84; 2.18)	0.237	2.28 (1.11; 4.67)	0.025
**IMD 2 in East midlands**	1.47 (0.95; 2.18)	0.083	1.46 (0.92; 2.33)	0.107
**IMD 2 in West midlands**	0.99 (0.71; 2.18)	0.940	1.16 (0.73; 1.84)	0.538
**IMD 2 in East of England**	0.76 (0.47; 2.18)	0.258	1.00 (0.59; 1.70)	0.993
**IMD 2 in South west**	1.03 (0.72; 2.18)	0.871	1.04 (0.67; 1.63)	0.847
**IMD 2 in South central**	0.93 (0.67; 2.18)	0.697	1.27 (0.82; 1.96)	0.278
**IMD 2 in South east coast**	1.17 (0.78; 2.18)	0.445	1.46 (0.92; 2.34)	0.111
**IMD 3 in North east**	1.45 (0.82; 3.18)	0.198	1.24 (0.69; 2.22)	0.468
**IMD 3 in North west**	1.05 (0.66; 3.18)	0.830	1.10 (0.64; 1.88)	0.725
**IMD 3 in Yorkshire and the Humber**	0.99 (0.62; 3.18)	0.960	0.74 (0.38; 1.43)	0.364
**IMD 3 in East midlands**	0.97 (0.62; 3.18)	0.882	1.81 (0.97; 3.37)	0.064
**IMD 3 in West midlands**	1.04 (0.75; 3.18)	0.804	1.03 (0.63; 1.67)	0.915
**IMD 3 in East of England**	0.91 (0.62; 3.18)	0.617	1.26 (0.74; 2.15)	0.387
**IMD 3 in South west**	1.04 (0.75; 3.18)	0.811	1.13 (0.71; 1.82)	0.598

**IMD 3 in South central**	1.02 (0.72; 3.18)	0.906	1.23 (0.75; 2.02)	0.403
**IMD 3 in South east coast**	1.26 (0.83; 3.18)	0.276	1.26 (0.75; 2.11)	0.382
**IMD 4 in North east**	1.43 (0.84; 4.18)	0.190	1.42 (0.81; 2.49)	0.223
**IMD 4 in North west**	1.22 (0.77; 4.18)	0.396	1.17 (0.71; 1.91)	0.537
**IMD 4 in Yorkshire and the Humber**	1.51 (0.95; 4.18)	0.081	1.24 (0.69; 2.24)	0.476
**IMD 4 in East midlands**	1.02 (0.68; 4.18)	0.925	1.71 (0.70; 4.18)	0.237
**IMD 4 in West midlands**	1.08 (0.78; 4.18)	0.655	1.01 (0.65; 1.57)	0.963
**IMD 4 in East of England**	1.04 (0.65; 4.18)	0.872	1.49 (0.83; 2.66)	0.182
**IMD 4 in South west**	0.94 (0.67; 4.18)	0.738	0.96 (0.62; 1.48)	0.854
**IMD 4 in South central**	0.89 (0.64; 4.18)	0.494	1.18 (0.77; 1.82)	0.441
**IMD 4 in South east coast**	1.42 (0.98; 4.18)	0.063	1.14 (0.68; 1.92)	0.623
**IMD 5 in North east**	1.80 (1.08; 5.18)	0.023	1.56 (0.94; 2.59)	0.087
**IMD 5 in North west**	1.13 (0.68; 5.18)	0.640	1.14 (0.70; 1.87)	0.600
**IMD 5 in Yorkshire and the Humber**	1.41 (0.95; 5.18)	0.089	1.28 (0.72; 2.29)	0.404
**IMD 5 in East midlands**	1.04 (0.71; 5.18)	0.856	1.51 (0.90; 2.52)	0.116
**IMD 5 in West midlands**	1.20 (0.87; 5.18)	0.258	0.97 (0.62; 1.51)	0.887
**IMD 5 in East of England**	0.99 (0.61; 5.18)	0.969	1.99 (1.11; 3.57)	0.021
**IMD 5 in South west**	1.06 (0.76; 5.18)	0.719	0.92 (0.59; 1.44)	0.715
**IMD 5 in South central**	1.21 (0.78; 5.18)	0.402	1.30 (0.76; 2.22)	0.345
**IMD 5 in South east coast**	1.91 (1.21; 5.18)	0.005	1.79 (1.14; 2.81)	0.011
**Ethnicity**				
**White**	Ref			
**Black**	0.78 (0.41; 1.49)	0.452	0.71 (0.31; 1.65)	0.430
**Asian**	0.87 (0.61; 1.23)	0.418	1.30 (0.88; 1.91)	0.189
**Other**	0.75 (0.56; 1.02)	0.070	0.94 (0.59; 1.51)	0.807
**Practice level IMD x ethnicity**				
**IMD 2 and black**	0.86 (0.34; 2.17)	0.743	0.47 (0.11; 1.92)	0.291
**IMD 2 and Asian**	1.13 (0.72; 1.77)	0.594	0.79 (0.45; 1.36)	0.393
**IMD 2 and other**	0.86 (0.54; 1.38)	0.538	0.94 (0.49; 1.84)	0.866
**IMD 3 and black**	0.77 (0.37; 1.61)	0.483	1.22 (0.47; 3.16)	0.676
**IMD 3 and Asian**	1.08 (0.69; 1.72)	0.728	0.68 (0.40; 1.18)	0.169
**IMD 3 and other**	1.11 (0.72; 1.71)	0.638	0.92 (0.48; 1.75)	0.788
**IMD 4 and black**	0.99 (0.49; 2.00)	0.979	1.19 (0.47; 3.00)	0.719
**IMD 4 and Asian**	1.07 (0.69; 1.65)	0.772	0.90 (0.53; 1.51)	0.679
**IMD 4 and other**	1.03 (0.66; 1.60)	0.912	0.86 (0.46; 1.63)	0.647
**IMD 5 and black**	1.08 (0.54; 2.17)	0.819	1.57 (0.64; 3.82)	0.321
**IMD 5 and Asian**	1.17 (0.76; 1.78)	0.477	0.85 (0.53; 1.37)	0.506
**IMD 5 and other**	1.32 (0.84; 2.09)	0.234	0.79 (0.36; 1.72)	0.556
**Sex**				
**Male**	Ref			
**Female**	1.00 (0.96; 1.03)	0.845	1.14 (1.09; 1.19)	<0.001

**Baseline age and BMI**				
**18-40**	Ref			
**41-54**	2.65 (1.56; 4.49)	<0.001	4.27 (1.43; 12.81)	0.010
**55-64**	3.74 (2.24; 6.24)	<0.001	4.64 (1.56; 13.80)	0.006
**65-74**	2.28 (1.37; 3.82)	0.002	1.39 (0.47; 4.12)	0.547
**75-84**	2.14 (1.28; 3.58)	0.004	1.71 (0.58; 5.05)	0.330
**≥85**	0.99 (0.59; 1.65)	0.966	2.06 (0.70; 6.08)	0.188
**Disease state and disability**				
**Heart failure**	0.97 (0.93; 1.02)	0.208	0.97 (0.91; 1.02)	0.233
**Cerebrovascular disease/TIA**	0.49 (0.46; 0.51)	<0.001	0.22 (0.21; 0.24)	0.000
**Hypertension**	0.72 (0.69; 0.76)	<0.001	0.31 (0.29; 0.33)	<0.001
**Diabetes**	1.02 (0.98; 1.06)	0.423	0.92 (0.87; 0.96)	0.001
**Rhuematological disease**	0.94 (0.89; 0.99)	0.017	0.89 (0.83; 0.96)	0.002
**Peptic ulcer**	0.80 (0.75; 0.85)	<0.001	0.83 (0.77; 0.90)	<0.001
**Aneamia**	0.71 (0.69; 0.74)	<0.001	0.83 (0.79; 0.87)	<0.001
**Dementia**	0.44 (0.39; 0.49)	<0.001	1.29 (1.11; 1.51)	0.001
**Malignancy**	0.75 (0.73; 0.78)	<0.001	0.80 (0.76; 0.83)	<0.001
**History of bleeding**	0.53 (0.50; 0.56)	<0.001	0.31 (0.29; 0.33)	<0.001
**Chronic kidney disease**	0.56 (0.53; 0.58)	<0.001	0.34 (0.32; 0.37)	<0.001
**Peripheral vascular disease**	0.92 (0.86; 0.97)	0.005	1.05 (0.97; 1.13)	0.193
**Ischaemic heart disease**	1.20 (1.16; 1.25)	<0.001	1.20 (1.14; 1.27)	<0.001
**Myocardial infarction**	1.03 (0.97; 1.09)	0.326	1.32 (1.23; 1.41)	<0.001
**Liver disease**	0.29 (0.25; 0.33)	<0.001	0.19 (0.16; 0.23)	<0.001
**Osteoporosis**	0.90 (0.87; 0.94)	<0.001	0.89 (0.84; 0.94)	<0.001
**Arthritis**	1.15 (1.11; 1.18)	<0.001	0.95 (0.91; 0.99)	0.027
**History of falls**	0.74 (0.71; 0.78)	<0.001	0.90 (0.85; 0.94)	<0.001
**Mobility problems**	0.74 (0.71; 0.78)	<0.001	0.95 (0.90; 1.01)	0.138
**Cognitive impairment**	0.75 (0.68; 0.82)	<0.001	0.68 (0.59; 0.79)	<0.001
**Visual impairment**	0.96 (0.92; 0.99)	0.014	0.99 (0.95; 1.04)	0.702
**HASBLED score**	1.72 (1.66; 1.78)	<0.001	3.05 (2.91; 3.20)	<0.001

* Adjusted for age, sex, comorbidities, socioeconomic status, HASBLED score, and takes in account clustering by general practice.
