# Supplementary material for: Incidence of nonvalvular atrial fibrillation and oral anticoagulant prescribing in England, 2009 to 2019: A cohort study
Source: PLoS Med. 2022 Jun 7;19(6):e1004003. doi: 10.1371/journal.pmed.1004003 (PMC9173622; doi:10.1371/journal.pmed.1004003)
Supplement: S12 Table — (PDF) [file pmed.1004003.s020.pdf]

S12 Table: Marginal analysis for the predictive probability of prescribing OAC, aspirin-only, or no treatment based on ethnicity and practice-level IMD

Treatment	Interaction (Practice IMD and Ethnicity)	Margins	95% CI	
No treatment	IMD 1 and White	17%	16%	17%
	IMD 1 and Black	20%	11%	29%
	IMD 1 and Asian	17%	13%	22%
	IMD 1 and other	20%	15%	24%
	IMD 2 and White	17%	16%	17%
	IMD 2 and Black	24%	13%	35%
	IMD 2 and Asian	17%	13%	20%
	IMD 2 and other	22%	16%	27%
	IMD 3 and White	17%	16%	17%
	IMD 3 and Black	23%	18%	29%
	IMD 3 and Asian	18%	14%	22%
	IMD 3 and other	19%	15%	23%
	IMD 4 and White	17%	17%	18%
	IMD 4 and Black	21%	17%	25%
	IMD 4 and Asian	18%	14%	22%
	IMD 4 and other	21%	16%	26%
	IMD 5 and White	17%	16%	18%
	IMD 5 and Black	18%	15%	22%
	IMD 5 and Asian	17%	14%	20%
	IMD 5 and other	18%	13%	22%
Aspirin-only	IMD 1 and White	13%	12%	13%
	IMD 1 and Black	11%	5%	18%
	IMD 1 and Asian	17%	14%	20%
	IMD 1 and other	14%	10%	19%
	IMD 2 and White	13%	12%	13%
	IMD 2 and Black	6%	1%	12%
	IMD 2 and Asian	13%	9%	17%
	IMD 2 and other	15%	11%	19%
	IMD 3 and White	13%	13%	14%
	IMD 3 and Black	16%	11%	22%
	IMD 3 and Asian	13%	10%	16%
	IMD 3 and other	13%	9%	18%
	IMD 4 and White	14%	14%	15%
	IMD 4 and Black	15%	11%	19%
	IMD 4 and Asian	17%	14%	20%
	IMD 4 and other	14%	10%	19%
	IMD 5 and White	15%	14%	16%
	IMD 5 and Black	18%	14%	22%

	IMD 5 and Asian	16%	13%	19%
	IMD 5 and other	12%	7%	17%
OAC prescribing	IMD 1 and White	71%	70%	72%
	IMD 1 and Black	69%	58%	79%
	IMD 1 and Asian	66%	61%	71%
	IMD 1 and other	66%	60%	72%
	IMD 2 and White	71%	70%	72%
	IMD 2 and Black	70%	60%	80%
	IMD 2 and Asian	70%	65%	75%
	IMD 2 and other	63%	57%	69%
	IMD 3 and White	70%	69%	71%
	IMD 3 and Black	61%	53%	68%
	IMD 3 and Asian	70%	65%	74%
	IMD 3 and other	68%	62%	73%
	IMD 4 and White	68%	67%	69%
	IMD 4 and Black	64%	59%	69%
	IMD 4 and Asian	65%	61%	69%
	IMD 4 and other	65%	59%	70%
	IMD 5 and White	68%	67%	70%
	IMD 5 and Black	64%	59%	68%
	IMD 5 and Asian	67%	63%	72%
	IMD 5 and other	71%	65%	76%
