# Supplementary material for: Incidence of nonvalvular atrial fibrillation and oral anticoagulant prescribing in England, 2009 to 2019: A cohort study
Source: PLoS Med. 2022 Jun 7;19(6):e1004003. doi: 10.1371/journal.pmed.1004003 (PMC9173622; doi:10.1371/journal.pmed.1004003)
Supplement: S13 Table — (PDF) [file pmed.1004003.s021.pdf]

**S13 Table: Marginal analysis for the predictive probability of prescribing OAC, aspirin-only, or no treatment based on practice region and practice-level IMD**

| Treatment | Interaction | Margins | 95% CI | |
|---|---|---|---|---|
| No treatment | London and IMD 1 | 17% | 13% | 20% |
| | London and IMD 2 | 18% | 16% | 19% |
| | London and IMD 3 | 18% | 15% | 20% |
| | London and IMD 4 | 19% | 17% | 21% |
| | London and IMD 4 | 20% | 18% | 21% |
| | North east and IMD 1 | 19% | 15% | 24% |
| | North east and IMD 2 | 14% | 12% | 17% |
| | North east and IMD 3 | 16% | 13% | 19% |
| | North east and IMD 4 | 17% | 14% | 19% |
| | North east and IMD 5 | 15% | 14% | 16% |
| | North west and IMD 1 | 19% | 15% | 23% |
| | North west and IMD2 | 18% | 17% | 20% |
| | North west and IMD 3 | 20% | 18% | 21% |
| | North west and IMD 4 | 19% | 17% | 21% |
| | North west and IMD 5 | 21% | 17% | 24% |
| | Yorkshire and the Humber and IMD 1 | 20% | 16% | 23% |
| | Yorkshire and the Humber and IMD 2 | 16% | 13% | 18% |
| | Yorkshire and the Humber and IMD 3 | 22% | 18% | 26% |
| | Yorkshire and the Humber and IMD 4 | 17% | 14% | 20% |
| | Yorkshire and the Humber and IMD 5 | 18% | 16% | 20% |
| | East midlands and IMD 1 | 16% | 15% | 17% |
| | East midlands and IMD 2 | 12% | 10% | 15% |
| | East midlands and IMD 3 | 16% | 13% | 19% |
| | East midlands and IMD 4 | 16% | 13% | 20% |
| | East midlands and IMD 5 | 17% | 15% | 20% |
| | West midlands and IMD 1 | 16% | 15% | 17% |
| | West midlands and IMD 2 | 17% | 15% | 18% |
| | West midlands and IMD 3 | 16% | 15% | 18% |
| | West midlands and IMD 4 | 17% | 16% | 18% |
| | West midlands and IMD 5 | 17% | 15% | 18% |
| | East of England and IMD 1 | 16% | 14% | 18% |
| | East of England and IMD 2 | 20% | 15% | 25% |
| | East of England and IMD 3 | 17% | 15% | 20% |
| | East of England and IMD 4 | 17% | 12% | 21% |
| | East of England and IMD 5 | 16% | 12% | 21% |
| | South west and IMD 1 | 14% | 13% | 15% |
| | South west and IMD 2 | 14% | 12% | 17% |
| | South west and IMD 3 | 14% | 13% | 15% |
| | South west and IMD 4 | 17% | 15% | 18% |
| | South west and IMD 5 | 16% | 14% | 18% |

| | | | | |
|---|---|---|---|---|
| | South central and IMD 1 | 16% | 14% | 17% |
| | South central and IMD 2 | 17% | 15% | 19% |
| | South central and IMD 3 | 16% | 14% | 18% |
| | South central and IMD 4 | 19% | 17% | 20% |
| | South central and IMD 5 | 16% | 12% | 19% |
| | South east coast and IMD 1 | 17% | 15% | 20% |
| | South east coast and IMD 2 | 16% | 13% | 19% |
| | South east coast and IMD 3 | 16% | 13% | 18% |
| | South east coast and IMD 4 | 16% | 14% | 18% |
| | South east coast and IMD 5 | 13% | 10% | 15% |
| Aspirin-only | Londond and IMD 1 | 15% | 12% | 18% |
| | Londond and IMD 2 | 13% | 12% | 15% |
| | Londond and IMD 3 | 15% | 13% | 17% |
| | Londond and IMD 4 | 16% | 14% | 19% |
| | Londond and IMD 4 | 17% | 15% | 19% |
| | North east and IMD 1 | 14% | 8% | 19% |
| | North east and IMD 2 | 11% | 7% | 15% |
| | North east and IMD 3 | 13% | 11% | 15% |
| | North east and IMD 4 | 16% | 13% | 19% |
| | North east and IMD 5 | 15% | 13% | 18% |
| | North west and IMD 1 | 12% | 10% | 15% |
| | North west and IMD2 | 14% | 12% | 15% |
| | North west and IMD 3 | 13% | 11% | 14% |
| | North west and IMD 4 | 14% | 12% | 15% |
| | North west and IMD 5 | 15% | 13% | 16% |
| | Yorkshire and the Humber and IMD 1 | 13% | 10% | 17% |
| | Yorkshire and the Humber and IMD 2 | 19% | 12% | 26% |
| | Yorkshire and the Humber and IMD 3 | 10% | 9% | 11% |
| | Yorkshire and the Humber and IMD 4 | 13% | 12% | 14% |
| | Yorkshire and the Humber and IMD 5 | 15% | 13% | 16% |
| | East midlands and IMD 1 | 12% | 9% | 15% |
| | East midlands and IMD 2 | 11% | 9% | 13% |
| | East midlands and IMD 3 | 19% | 13% | 26% |
| | East midlands and IMD 4 | 20% | 8% | 32% |
| | East midlands and IMD 5 | 19% | 15% | 22% |
| | West midlands and IMD 1 | 12% | 10% | 14% |
| | West midlands and IMD 2 | 12% | 11% | 14% |
| | West midlands and IMD 3 | 12% | 11% | 13% |
| | West midlands and IMD 4 | 13% | 12% | 14% |
| | West midlands and IMD 5 | 12% | 11% | 13% |
| | East of England and IMD 1 | 14% | 12% | 15% |
| | East of England and IMD 2 | 14% | 11% | 16% |
| | East of England and IMD 3 | 17% | 15% | 20% |

| | | | | |
|---|---|---|---|---|
| | East of England and IMD 4 | 20% | 15% | 25% |
| | East of England and IMD 5 | 26% | 19% | 33% |
| | South west and IMD 1 | 12% | 11% | 14% |
| | South west and IMD 2 | 11% | 9% | 13% |
| | South west and IMD 3 | 13% | 12% | 15% |
| | South west and IMD 4 | 14% | 12% | 16% |
| | South west and IMD 5 | 13% | 11% | 14% |
| | South central and IMD 1 | 12% | 11% | 13% |
| | South central and IMD 2 | 13% | 12% | 15% |
| | South central and IMD 3 | 14% | 12% | 16% |
| | South central and IMD 4 | 16% | 14% | 18% |
| | South central and IMD 5 | 15% | 10% | 20% |
| | South east coast and IMD 1 | 11% | 10% | 13% |
| | South east coast and IMD 2 | 12% | 11% | 14% |
| | South east coast and IMD 3 | 12% | 10% | 14% |
| | South east coast and IMD 4 | 11% | 8% | 14% |
| | South east coast and IMD 5 | 14% | 10% | 18% |
| OAC prescribing | Londond and IMD 1 | 68% | 65% | 71% |
| | Londond and IMD 2 | 69% | 67% | 72% |
| | Londond and IMD 3 | 67% | 65% | 69% |
| | Londond and IMD 4 | 65% | 61% | 68% |
| | Londond and IMD 5 | 63% | 61% | 66% |
| | North east and IMD 1 | 67% | 58% | 77% |
| | North east and IMD 2 | 74% | 68% | 80% |
| | North east and IMD 3 | 71% | 68% | 74% |
| | North east and IMD 4 | 67% | 65% | 70% |
| | North east and IMD 5 | 70% | 68% | 72% |
| | North west and IMD 1 | 69% | 63% | 74% |
| | North west and IMD2 | 68% | 66% | 70% |
| | North west and IMD 3 | 68% | 65% | 70% |
| | North west and IMD 4 | 68% | 65% | 70% |
| | North west and IMD 5 | 65% | 60% | 70% |
| | Yorkshire and the Humber and IMD 1 | 67% | 63% | 71% |
| | Yorkshire and the Humber and IMD 2 | 65% | 58% | 73% |
| | Yorkshire and the Humber and IMD 3 | 68% | 64% | 71% |
| | Yorkshire and the Humber and IMD 4 | 70% | 66% | 73% |
| | Yorkshire and the Humber and IMD 5 | 67% | 64% | 69% |
| | East midlands and IMD 1 | 72% | 69% | 75% |
| | East midlands and IMD 2 | 77% | 72% | 81% |
| | East midlands and IMD 3 | 65% | 57% | 72% |
| | East midlands and IMD 4 | 64% | 53% | 75% |
| | East midlands and IMD 5 | 64% | 59% | 69% |
| | West midlands and IMD 1 | 72% | 70% | 74% |

| | | | | |
|---|---|---|---|---|
| | West midlands and IMD 2 | 71% | 69% | 74% |
| | West midlands and IMD 3 | 71% | 70% | 73% |
| | West midlands and IMD 4 | 70% | 68% | 71% |
| | West midlands and IMD 5 | 71% | 69% | 73% |
| | East of England and IMD 1 | 71% | 68% | 73% |
| | East of England and IMD 2 | 66% | 60% | 72% |
| | East of England and IMD 3 | 65% | 62% | 68% |
| | East of England and IMD 4 | 64% | 58% | 70% |
| | East of England and IMD 5 | 58% | 49% | 66% |
| | South west and IMD 1 | 74% | 72% | 76% |
| | South west and IMD 2 | 75% | 71% | 78% |
| | South west and IMD 3 | 72% | 71% | 74% |
| | South west and IMD 4 | 70% | 67% | 73% |
| | South west and IMD 5 | 71% | 69% | 74% |
| | South central and IMD 1 | 73% | 71% | 74% |
| | South central and IMD 2 | 70% | 67% | 73% |
| | South central and IMD 3 | 70% | 67% | 73% |
| | South central and IMD 4 | 65% | 63% | 68% |
| | South central and IMD 5 | 69% | 63% | 76% |
| | South east coast and IMD 1 | 71% | 68% | 74% |
| | South east coast and IMD 2 | 72% | 68% | 76% |
| | South east coast and IMD 3 | 73% | 68% | 77% |
| | South east coast and IMD 4 | 73% | 69% | 77% |
| | South east coast and IMD 5 | 74% | 67% | 80% |
