# Supplementary material for: Incidence of nonvalvular atrial fibrillation and oral anticoagulant prescribing in England, 2009 to 2019: A cohort study
Source: PLoS Med. 2022 Jun 7;19(6):e1004003. doi: 10.1371/journal.pmed.1004003 (PMC9173622; doi:10.1371/journal.pmed.1004003)
Supplement: S14 Table — (PDF) [file pmed.1004003.s022.pdf]

S14 Table: Results of the unadjusted univariate analysis evaluating factors associated prescribing of OAC or aspirin- only vs no treatment (reference group) in patients recommended to take OAC

	OAC		Aspirin only	
	Unadjusted RRR 95%CI	P value	Unadjusted RRR 95%CI	P value
**Region**				
**London**	Ref			
**North east**	1.35 (1.25; 1.46)	<0.001	1.29 (1.16; 1.42)	<0.001
**North west**	1.04 (0.99; 1.10)	0.122	0.93 (0.86; 1.00)	0.036
**Yorkshire and the Humber**	1.07 (0.99; 1.16)	0.089	0.96 (0.87; 1.07)	0.493
**East midlands**	1.32 (1.19; 1.46)	<0.001	1.24 (1.09; 1.42)	0.001
**West midlands**	1.27 (1.21; 1.34)	<0.001	0.94 (0.88; 1.01)	0.113
**East of England**	1.12 (1.04; 1.20)	0.001	1.15 (1.05; 1.26)	0.002
**South west**	1.39 (1.32; 1.47)	<0.001	1.08 (1.00; 1.16)	0.047
**South central**	1.21 (1.14; 1.29)	<0.001	1.00 (0.92; 1.08)	0.969
**South east coast**	1.27 (1.20; 1.35)	<0.001	0.89 (0.81; 0.96)	0.005
**Practice level IMD**				
**1 (least deprived)**	Ref			
**2**	0.99 (0.95; 1.03)	0.633	1.02 (0.96; 1.09)	0.483
**3**	1.01 (0.96; 1.05)	0.766	1.08 (0.96; 1.09)	0.016
**4**	0.90 (0.86; 0.94)	<0.001	1.12 (9.96; 0.09)	<0.001
**5 (most deprived)**	0.92 (0.88; 0.96)	<0.001	1.10 (9.96; 0.09)	0.001
**Patient level IMD**				
**1 (least deprived)**	Ref			
**2**	0.95 (0.91; 0.99)	0.008	1.09 (1.03; 1.15)	0.004
**3**	0.92 (0.88; 0.96)	<0.001	1.13 (1.07; 1.20)	<0.001
**4**	0.87 (0.84; 0.91)	<0.001	1.14 (1.07; 1.21)	<0.001
**5 (most deprived)**	0.80 (0.77; 0.84)	<0.001	1.14 (1.08; 1.21)	<0.001
**Ethnicity**				
**White**	Ref			
**Black**	0.70 (0.61; 0.80)	<0.001	0.85 (0.70; 1.02)	0.084
**Asian**	0.99 (0.88; 1.11)	0.847	1.08 (0.92; 1.26)	0.345
**Other**	0.80 (0.69; 0.93)	0.004	0.80 (0.65; 0.98)	0.033
**Sex**				
**Male**	Ref			
**Female**	0.77 (0.75; 0.79)	<0.001	0.96 (0.93; 1.00)	0.038
**Baseline age and BMI**				
**18-40**	Ref			
**41-54**	3.04 (1.85; 4.98)	<0.001	5.12 (1.76; 14.83)	0.003
**55-64**	4.53 (2.80; 7.33)	<0.001	6.02 (2.10; 17.24)	0.001
**65-74**	4.53 (2.82; 7.29)	<0.001	4.97 (1.74; 14.14)	0.003

**75-84**	3.59 (2.23; 5.76)	<0.001	5.50 (1.93; 15.64)	0.001
**≥85**	1.41 (0.88; 2.27)	0.153	6.04 (2.12; 17.17)	0.001
**BMI**	1.00 (1.00; 1.00)	0.845	1.00 (1.00; 1.00)	0.645
**Disease state and disability**				
**Heart failure**	0.94 (0.90; 0.98)	0.002	1.17 (1.11; 1.24)	<0.001
**Cerebrovascular disease/TIA**	0.88 (0.85; 0.91)	<0.001	0.94 (0.90; 0.98)	0.006
**Hypertension**	1.31 (1.27; 1.35)	<0.001	1.02 (0.98; 1.07)	0.245
**Diabetes**	1.11 (1.08; 1.15)	<0.001	0.98 (0.94; 1.02)	0.284
**Rhuematological disease**	0.87 (0.82; 0.91)	<0.001	0.88 (0.82; 0.94)	<0.001
**Peptic ulcer**	0.74 (0.70; 0.78)	<0.001	0.84 (0.78; 0.91)	<0.001
**HIV/AIDS**	0.44 (0.20; 0.96)	0.039	0.51 (0.16; 1.64)	0.259
**Aneamia**	0.60 (0.58; 0.62)	<0.001	0.84 (0.81; 0.88)	<0.001
**Dementia**	0.24 (0.22; 0.25)	<0.001	0.94 (0.88; 1.00)	0.050
**Malignancy**	0.69 (0.67; 0.71)	<0.001	0.79 (0.76; 0.83)	<0.001
**History of bleeding**	0.80 (0.77; 0.83)	<0.001	0.90 (0.85; 0.94)	<0.001
**Chronic kidney disease**	0.80 (0.78; 0.83)	<0.001	1.12 (1.08; 1.16)	<0.001
**Peripheral vascular disease**	1.02 (0.97; 1.08)	0.421	1.37 (1.28; 1.47)	<0.001
**Ischaemic heart disease**	1.36 (1.32; 1.40)	<0.001	1.73 (1.67; 1.80)	<0.001
**Myocardial infarction**	1.38 (1.32; 1.45)	<0.001	2.10 (1.99; 2.22)	<0.001
**Liver disease**	0.57 (0.50; 0.64)	<0.001	0.61 (0.51; 0.74)	<0.001
**Respiratory disease**	0.97 (0.94; 1.00)	0.022	0.90 (0.86; 0.93)	<0.001
**Parkinsonism**	0.74 (0.69; 0.79)	<0.001	0.94 (0.85; 1.03)	0.161
**Osteoporosis**	0.66 (0.64; 0.69)	<0.001	0.86 (0.82; 0.91)	<0.001
**Arthritis**	0.99 (0.96; 1.02)	0.528	0.97 (0.93; 1.00)	0.076
**Skin ulcer**	0.65 (0.61; 0.68)	<0.001	1.01 (0.94; 1.08)	0.857
**History of falls**	0.53 (0.51; 0.54)	<0.001	0.92 (0.88; 0.96)	0.000
**Dizziness**	0.97 (0.94; 1.00)	0.032	0.99 (0.95; 1.03)	0.659
**Fragility fractures**	0.66 (0.64; 0.69)	<0.001	0.77 (0.72; 0.82)	<0.001
**Mobility problems**	0.59 (0.56; 0.61)	<0.001	1.04 (0.98; 1.10)	0.176
**Cognitive impairment**	0.34 (0.33; 0.36)	<0.001	0.84 (0.80; 0.90)	<0.001
**Activity limitation**	0.73 (0.68; 0.79)	<0.001	0.89 (0.81; 0.97)	0.013
**Visual impairment**	0.77 (0.74; 0.79)	<0.001	1.04 (1.01; 1.08)	0.019
**Require care**	0.45 (0.43; 0.48)	<0.001	0.97 (0.90; 1.04)	0.365
**Socially vulnerable**	0.60 (0.57; 0.62)	<0.001	0.85 (0.81; 0.89)	<0.001
**Housebound**	0.48 (0.47; 0.50)	<0.001	1.05 (1.01; 1.09)	0.013
**Baseline drug-use**				
**Polypharmacy**	0.94 (0.92; 0.97)	<0.001	0.88 (0.85; 0.92)	<0.001
**Antibiotics**	0.78 (0.74; 0.83)	<0.001	0.90 (0.84; 0.97)	0.008
**Antiepileptic's**	0.84 (0.71; 0.99)	0.041	1.54 (1.26; 1.89)	<0.001

**Calcium channel blockers**	1.51 (1.35; 1.68)	<0.001	1.75 (1.53; 2.01)	<0.001
**Corticosteroids**	0.80 (0.77; 0.84)	<0.001	0.82 (0.77; 0.87)	<0.001
**Antiplatelets**	0.85 (0.82; 0.88)	<0.001	0.58 (0.54; 0.61)	<0.001
**SSRI/SNRI**	0.73 (0.69; 0.77)	<0.001	1.03 (0.97; 1.10)	0.295
**Statins**	1.87 (1.81; 1.92)	<0.001	1.46 (1.41; 1.51)	<0.001
**Trizoles**	0.62 (0.51; 0.76)	<0.001	0.69 (0.53; 0.91)	0.009
**PPI**	0.97 (0.94; 1.00)	0.022	1.02 (0.98; 1.06)	0.329
**NSAIDs**	1.38 (1.30; 1.46)	<0.001	1.29 (1.20; 1.39)	<0.001
**Smoking status**				
**Non-smoker/Ex-smoker**	Ref			
**Current smoker**	0.90 (0.87; 0.93)	<0.001	0.93 (0.86; 0.98)	0.002
**Alcohol consumption status**				
**Non-drinker**	Ref			
**Light drinker**	1.31 (1.24; 1.38)	<0.001	1.06 (0.99; 1.14)	0.108
**Former drinker**	1.08 (0.96; 1.22)	0.191	1.23 (1.06; 1.43)	0.006
**Moderate drinker**	1.40 (1.35; 1.46)	<0.001	0.97 (0.92; 1.02)	0.285
**Heavy drinker**	1.28 (1.21; 1.36)	<0.001	0.78 (0.72; 0.85)	<0.001
