# Supplementary material for: Incidence of nonvalvular atrial fibrillation and oral anticoagulant prescribing in England, 2009 to 2019: A cohort study
Source: PLoS Med. 2022 Jun 7;19(6):e1004003. doi: 10.1371/journal.pmed.1004003 (PMC9173622; doi:10.1371/journal.pmed.1004003)
Supplement: S15 Table — (PDF) [file pmed.1004003.s023.pdf]

S15 Table: Sensitivity analysis using multivariable binomial logistic regression to investigate the association between patient characteristics and prescribing of oral anticoagulants

	Unadjusted OR 95%CI	P value	Adjusted OR* 95%CI	P value
**Region**				
**London**	Ref			
**North east**	1.20 (1.13; 1.28)	<0.001	1.26 (1.17; 1.35)	<0.001
**North west**	1.08 (1.03; 1.13)	0.001	1.07 (1.01; 1.13)	0.012
**Yorkshire and the Humber**	1.09 (1.02; 1.16)	0.010	1.05 (0.97; 1.13)	0.231
**East midlands**	1.19 (1.10; 1.29)	<0.001	1.10 (1.00; 1.21)	0.050
**West midlands**	1.31 (1.25; 1.36)	<0.001	1.29 (1.22; 1.36)	<0.001
**East of England**	1.05 (0.99; 1.11)	0.081	0.96 (0.90; 1.02)	0.201
**South west**	1.35 (1.29; 1.41)	<0.001	1.37 (1.29; 1.44)	<0.001
**South central**	1.21 (1.16; 1.27)	<0.001	1.13 (1.07; 1.20)	<0.001
**South east coast**	1.34 (1.27; 1.41)	<0.001	1.29 (1.22; 1.38)	<0.001
**Practice level IMD**				
**1 (least deprived)**	Ref			
**2**	0.99 (0.94; 1.03)	0.506	0.98 (0.95; 1.02)	0.273
**3**	0.98 (0.94; 1.02)	0.311	0.98 (0.94; 1.01)	0.162
**4**	0.90 (0.86; 0.94)	<0.001	0.86 (0.83; 0.89)	<0.001
**5 (most deprived)**	0.95 (0.90; 0.99)	0.027	0.88 (0.85; 0.91)	<0.001
**Practice size (per 1000)**	0.997 (0.995; 0.998)	<0.001	0.999 (0.997; 1.001)	0.218
**Patient level IMD**				
**1 (least deprived)**	Ref			
**2**	0.91 (0.89; 0.95)	<0.001	0.93 (0.90; 0.97)	0.001
**3**	0.87 (0.84; 0.90)	<0.001	0.89 (0.86; 0.93)	<0.001
**4**	0.83 (0.80; 0.86)	<0.001	0.90 (0.86; 0.94)	<0.001
**5 (most deprived)**	0.76 (0.73; 0.79)	<0.001	0.82 (0.78; 0.86)	<0.001
**Ethnicity**				
**White**	Ref			
**Black**	0.75 (0.67; 0.84)	<0.001	0.78 (0.68; 0.90)	0.001
**Asian**	0.96 (0.87; 1.05)	0.341	0.89 (0.78; 1.00)	0.044
**Other**	0.88 (0.78; 0.99)	0.049	0.79 (0.68; 0.91)	0.001
**Sex**				
**Male**	Ref			
**Female**	0.79 (0.77; 0.80)	<0.001	1.04 (1.01; 1.07)	0.004
**Baseline age and BMI**				
**18-40**	Ref			
**41-54**	2.03 (1.27; 3.24)	0.003	1.54 (0.87; 2.73)	0.139
**55-64**	2.82 (1.78; 4.46)	<0.001	2.11 (1.19; 3.70)	0.010

**65-74**	3.06 (1.94; 4.83)	<0.001	2.23 (1.27; 3.90)	0.005
**75-84**	2.32 (1.47; 3.67)	<0.001	1.97 (1.23; 3.44)	0.018
**≥85**	0.88 (0.56; 1.38)	0.574	0.93 (0.53; 1.63)	0.809
**BMI**	1.069 (1.067; 1.072)	<0.001	1.044 (1.041; 1.047)	<0.001
**Disease state and disability**				
**Heart failure**	0.87 (0.84; 0.90)	<0.001	1.01 (0.98; 1.05)	0.479
**Cerebrovascular disease/TIA**	0.91 (0.88; 0.93)	<0.001	0.96 (0.93; 0.99)	0.021
**Hypertension**	1.29 (1.26; 1.33)	<0.001	1.19 (1.15; 1.22)	0.000
**Diabetes**	1.12 (1.09; 1.15)	<0.001	1.00 (0.97; 1.03)	0.878
**Rhuematological disease**	0.91 (0.88; 0.95)	<0.001	1.02 (0.97; 1.07)	0.397
**Peptic ulcer**	0.80 (0.76; 0.83)	<0.001	0.88 (0.84; 0.93)	<0.001
**HIV/AIDS**	0.56 (0.28; 1.13)	0.108	0.55 (0.24; 1.25)	0.154
**Aneamia**	0.64 (0.63; 0.66)	<0.001	0.78 (0.76; 0.81)	<0.001
**Dementia**	0.24 (0.23; 0.26)	<0.001	0.45 (0.41; 0.50)	<0.001
**Malignancy**	0.76 (0.74; 0.78)	<0.001	0.81 (0.78; 0.83)	<0.001
**History of bleeding**	0.84 (0.81; 0.86)	<0.001	0.94 (0.90; 0.97)	<0.001
**Chronic kidney disease**	0.76 (0.74; 0.78)	<0.001	0.93 (0.90; 0.96)	<0.001
**Peripheral vascular disease**	0.88 (0.85; 0.92)	<0.001	0.92 (0.87; 0.96)	<0.001
**Ischaemic heart disease**	1.05 (1.03; 1.08)	<0.001	1.00 (0.97; 1.04)	0.786
**Myocardial infarction**	0.95 (0.92; 0.98)	<0.001	0.86 (0.82; 0.90)	<0.001
**Liver disease**	0.68 (0.61; 0.76)	<0.001	0.63 (0.56; 0.71)	<0.001
**Respiratory disease**	1.01 (0.99; 1.04)	0.208	1.06 (1.03; 1.09)	<0.001
**Parkinsonism**	0.76 (0.72; 0.81)	<0.001	0.90 (0.84; 0.97)	0.003
**Osteoporosis**	0.70 (0.68; 0.73)	<0.001	0.98 (0.94; 1.02)	0.309
**Arthritis**	1.01 (0.98; 1.03)	0.602	1.15 (1.12; 1.18)	<0.001
**Skin ulcer**	0.65 (0.62; 0.67)	<0.001	0.84 (0.79; 0.88)	<0.001
**History of falls**	0.55 (0.53; 0.56)	<0.001	0.83 (0.80; 0.86)	<0.001
**Dizziness**	0.97 (0.95; 0.99)	0.020	1.08 (1.05; 1.12)	<0.001
**Fragility fractures**	0.74 (0.72; 0.77)	<0.001	0.98 (0.93; 1.03)	0.413
**Mobility problems**	0.58 (0.56; 0.60)	<0.001	0.82 (0.78; 0.85)	<0.001
**Cognitive impairment**	0.37 (0.35; 0.38)	<0.001	1.23 (1.12; 1.2)	<0.001
**Activity limitation**	0.77 (0.73; 0.82)	<0.001	1.24 (1.15; 1.34)	<0.001
**Visual impairment**	0.75 (0.73; 0.77)	<0.001	1.03 (0.99; 1.07)	0.213
**Require care**	0.46 (0.44; 0.48)	<0.001	1.24 (1.14; 1.36)	<0.001
**Socially vulnerable**	0.64 (0.62; 0.66)	<0.001	1.07 (1.01; 1.12)	0.012
**Housebound**	0.47 (0.46; 0.49)	<0.001	1.33 (1.27; 1.39)	<0.001
**Baseline drug-use**				
**Polypharmacy**	0.99 (0.97; 1.01)	0.509	1.02 (0.90; 1.05)	<0.001
**Antibiotics**	0.82 (0.78; 0.86)	<0.001	0.91 (0.86; 0.96)	0.001
**Antiepileptic's**	0.67 (0.60; 0.77)	<0.001	0.73 (0.63; 0.85)	<0.001

**Calcium channel blockers**	1.14 (1.04; 1.23)	0.002	1.05 (0.96; 1.15)	0.265
**Corticosteroids**	0.87 (0.84; 0.90)	<0.001	0.87 (0.83; 0.91)	<0.001
**Antiplatelets**	1.05 (1.02; 1.09)	0.003	1.06 (1.01; 1.11)	0.009
**SSRI/SNRI**	0.72 (0.69; 0.75)	<0.001	0.84 (0.80; 0.88)	<0.001
**Statins**	1.58 (1.54; 1.61)	<0.001	1.43 (1.38; 1.47)	<0.001
**Trizoles**	0.72 (0.61; 0.85)	<0.001	0.74 (0.61; 0.89)	0.002
**PPI**	0.96 (0.94; 0.98)	<0.001	1.03 (0.99; 1.06)	0.056
**NSAIDs**	1.22 (1.17; 1.28)	<0.001	1.01 (0.96; 1.07)	0.640
**Smoking status**				
**Non-smoker/Ex-smoker**	Ref			
**Current smoker**	0.92 (0.90; 0.95)	<0.001	0.80 (0.78; 0.82)	<0.001
**Alcohol consumption status**				
**Non-drinker**	Ref			
**Light drinker**	1.27 (1.22; 1.33)	<0.001	1.11 (1.06; 1.17)	<0.001
**Former drinker**	0.98 (0.89; 1.08)	0.696	0.95 (0.86; 1.05)	0.310
**Moderate drinker**	1.41 (1.37; 1.47)	<0.001	1.13 (1.09; 1.17)	<0.001
**Heavy drinker**	1.42 (1.36; 1.49)	<0.001	1.00 (0.95; 1.06)	0.959

* Adjusted for age, sex, comorbidities, socioeconomic status, baseline treatment, frailty and bleeding risk factors

Abbreviations: BMI, body mass index; CCB, calcium channel blocker; HIV, human immunodeficiency virus; IMD, index of multiple deprivation; NOAC, non-vitamin K antagonist; NSAIDs, non-steroidal anti-inflammatory drugs; PPI, proton pump inhibitors; SSRI/SNRI, selective serotonin reuptake inhibitors/selective norepinephrine reuptake inhibitors; TIA, transient ischaemic attack.
